# Structure and transcription of integrated HPV DNA in vulvar carcinomas

**DOI:** 10.1038/s41525-024-00418-8

**Published:** 2024-06-19

**Authors:** Anne Van Arsdale, Lauren Turker, Yoke-Chen Chang, Joshua Gould, Bryan Harmon, Elaine C. Maggi, Olga Meshcheryakova, Maxwell P. Brown, Dana Luong, Koenraad Van Doorslaer, Mark H. Einstein, Dennis Y. S. Kuo, Deyou Zheng, Brian J. Haas, Jack Lenz, Cristina Montagna

**Affiliations:** 1 Department of Obstetrics Gynecology and Women’s Health, Albert Einstein College of Medicine, Bronx, NY 10461 USA; 2 Department of Genetics, Albert Einstein College of Medicine, Bronx, NY 10461 USA; 3 Rutgers Cancer Institute of New Jersey, 195 Little Albany St., New Brunswick, NJ 08901 USA; 4 Broad Institute, Cambridge, MA 02142 USA; 5 Department of Pathology, Albert Einstein College of Medicine, Bronx, NY 10461 USA; 6 School of Animal and Comparative Biomedical Sciences, College of Agriculture and Life Sciences BIO5 Institute, University of Arizona, Tucson, AZ 85721 USA; 7 Department of Obstetrics, Gynecology, and Women’s Health, Rutgers New Jersey Medical School, Newark, NJ 07102 USA; 8 Present Address: Lankenau Medical Center, Wynnewood, PA 19096 USA; 9 Present Address: Cellarity, Cambridge, MA 02140 USA; 10 Present Address: Twist Biosciences, South San Francisco, CA 94080 USA; 11 Present Address: Verve Therapeutics, Boston, MA 02215 USA

**Keywords:** Cancer, Gynaecological cancer

## Abstract

HPV infections are associated with a fraction of vulvar cancers. Through hybridization capture and DNA sequencing, HPV DNA was detected in five of thirteen vulvar cancers. HPV16 DNA was integrated into human DNA in three of the five. The insertions were in introns of human *NCKAP1*, *C5orf67*, and *LRP1B*. Integrations in *NCKAP1* and *C5orf67* were flanked by short direct repeats in the human DNA, consistent with HPV DNA insertions at sites of abortive, staggered, endonucleolytic incisions. The insertion in *C5orf67* was present as a 36 kbp, human-HPV-hetero-catemeric DNA as either an extrachromosomal circle or a tandem repeat within the human genome. The human circularization/repeat junction was defined at single nucleotide resolution. The integrated viral DNA segments all retained an intact upstream regulatory region and the adjacent viral E6 and E7 oncogenes. RNA sequencing revealed that the only HPV genes consistently transcribed from the integrated viral DNAs were E7 and E6*I. The other two HPV DNA+ tumors had coinfections, but no evidence for integration. HPV-positive and HPV-negative vulvar cancers exhibited contrasting human, global gene expression patterns partially overlapping with previously observed differences between HPV-positive and HPV-negative cervical and oropharyngeal cancers. A substantial fraction of the differentially expressed genes involved immune system function. Thus, transcription and HPV DNA integration in vulvar cancers resemble those in other HPV-positive cancers. This study emphasizes the power of hybridization capture coupled with DNA and RNA sequencing to identify a broad spectrum of HPV types, determine human genome integration status of viral DNAs, and elucidate their structures.

## Introduction

Vulvar cancer is relatively uncommon comprising ~5% of gynecologic malignancies, with human papillomavirus (HPV) infections underlying a fraction of those cases. Currently, the annual incidence of vulvar cancer in the United States is about 2.6 cases per 100,000 women, with a death rate of around 0.6 per 100,000 women and a 5 year survival rate of about 70%^1^. In 2020, the Centers for Disease Control and Prevention (CDC) and The Surveillance, Epidemiology, and End Results (SEER) reported 6120 new cases of vulvar cancer and 1350 deaths from vulvar cancer^1^. When the disease is localized to the vulva, the 5-year overall survival is about 85%, but this number starkly decreases to 50% when the disease has spread to inguinal lymph nodes^2^.

Multiple different histologic types of vulvar carcinoma exist, with 90% being squamous cell carcinomas (SCCs). Two distinct pathways lead to SCC of the vulva^3^. One is associated with HPV infection and leads to either warty or basaloid squamous cell carcinoma of the vulva. The other arises in the background of lichen sclerosis, a chronic immunological skin condition of the external genitalia which develops into keratinizing squamous cell vulvar carcinoma. Based on the available literature,15–80% of SCC of the vulva may be secondary to HPV infections^4–7^. This wide range of HPV association with SCC among different studies may be due to the rarity of vulvar SCC and HPV infection being assessed only in research settings on a relatively modest number of cases. Differences among populations studied, and utilization of HPV detection procedures that have different HPV type specificities may also contribute to the wide range of HPV frequency reported. Regardless, recent studies indicated that HPV-negative vulvar SCC have worse clinical outcomes as compared to HPV-positive counterparts^8,9^.

HPV is a double-stranded DNA virus with a circular genome of roughly 8 kbp. To date, well over 200 HPV types have been identified and fully sequenced^10^. The viral life cycle and how it causes disease have been most thoroughly studied in cervical dysplasia and carcinomas, where initial infections are established in the basal layer of the epithelia. As epithelial cells differentiate, viral early genes are sequentially expressed, replication of viral DNA episomes ensues, viral late genes are ultimately expressed, and new virions are assembled. While genitourinary tract HPV infections are widespread in humans, the vast majority are transient in nature, presumably as a consequence of the immune responses, with only a small fraction of infected individuals ever developing an invasive cancer^11,12^. In vulvar cancer, the relative contributions of high-risk HPV types 16 and type 18 in HPV-positive tumors is estimated at roughly 70%, which is similar to cancers of the cervix^13^.

An important advance in understanding cervical carcinogenesis was the discovery that HPV DNA is integrated into the human genome in most HPV-induced tumors^12^. Viral DNA insertions are assumed to be rare events presumably occurring as a consequence of aberrant cellular double-stranded DNA break repair pathways^14–20^. The role of viral integration to tumorigenesis may be multifactorial including stable, constitutive expression of the viral oncoproteins E6 and E7, induction of human genome instability, and altered structure and/or expression of human cancer genes as a consequence of integrated viral DNA. The extent of HPV DNA integration in HPV-dependent vulvar carcinomas, and whether it resembles the patterns observed in cervical cancers and other HPV-associated tumors, particularly head and neck squamous cell carcinomas (HNSCCs), remained unknown. Hence, our objective was to assess HPV DNA integration, genome structure, and transcription in a cohort of vulvar carcinomas using a custom, hybridization capture (HC) method capable of detecting multiple HPV types, followed by Illumina massively parallel high-throughput sequencing (HC + SEQ)^21^. To further enhance our analysis, we employed long-range nanopore DNA sequencing and Illumina RNA-seq to examine the tumor transcriptomes.

## Results

### PCR assay for the HPV L1 gene detected HPV infection in a subset of vulvar squamous cell carcinomas (VSCCs)

To assess the presence of HPV DNA in Vulvar Squamous Cell Carcinomas (VSCCs), genomic DNA was extracted from 13 vulvar tumors, all of which were VSCCs (Table 1). The initial screening involved PCR amplification using primers (GP5+/GP6+) that target a conserved region within the viral L1 gene, shared by multiple mucosotropic HPV types^22^. The overall median age of the cohort was 67.07 years [range: 43–85] and included various self-reported ethnicities. Six of the 13 tumors (46%) had recurrent disease at the time of biopsy, and three samples (23%) were from women that received neoadjuvant radiotherapy and chemotherapy prior to tumor biopsy. DNAs were extracted from 12 primary vulvar tumor sites, and 1 metastatic site biopsy (Tumor 2). HPV DNA was detected in tumors 2, 4, 5, and 10 (31% of the samples) (Supplementary Fig. 1), indicating the presence of HPV DNA in the tumors, but not whether the viral DNA was present in episomal or integrated form (Table 2).

**Table 1:** Clinical characteristics of patients and vulvar tumors included in the study

Sample	Age (years)	Ethnicity^a^	Histology	Disease status at sample acquisition	Prior therapy (chemotherapy and/or radiation)
Tumor 1	66	White	VVSC	Primary	No
Tumor 2	65	Black	VVSC	Primary	No
Tumor 3	74	Unk	VVSC	Primary	Yes
Tumor 4	45	White	VVSC	Primary	No
Tumor 5	43	Black	VVSC	Recurrence	Yes
Tumor 6	64	White	VVSC	Primary	No
Tumor 7	83	White	VVSC	Recurrence	Yes
Tumor 8	81	White	VVSC	Recurrence	No
Tumor 9	75	White	VVSC	Primary	No
Tumor 10	85	Black	VVSC	Recurrence	No
Tumor 11	61	Unk	VVSC	Primary	No
Tumor 12	77	Hispanic	VVSC	Recurrence	No
Tumor 13	53	Hispanic	VVSC	Recurrence	No^b^
	67.07 ± 3.8	White = 6 Black = 3 Hispanic = 2 Unk = 2	VVSC = 13	Primary = 7 Recurrence = 6	Yes = 3 No = 10

*Unk* unknown.

^a^self-reported.

^b^this patient was HIV positive.

**Table 2:** HPV status, HPV type, and integration sites detected in VVSC

Sample	HPV status by HC + SEQ	HPV Type(s)	Integration Site/Gene
Tumor 1	Negative	ND	NA
Tumor 2	Positive	16	Chr. 2/*NCKAP1*
Tumor 3	Negative	ND	NA
Tumor 4	Positive	16	Chr. 5/*C5orf67*
Tumor 5	Positive	16	Chr. 2/*LRP1B*
Tumor 6	Negative	ND	NA
Tumor 7	Negative	ND	NA
Tumor 8	Negative	ND	NA
Tumor 9	Negative	ND	NA
Tumor 10	Positive	6, 16	No integration
Tumor 11	Negative	ND	NA
Tumor 12	Negative	ND	NA
Tumor 13	Positive	53, 62	No integration

*ND* not detected, *NA* not applicable.

### Comprehensive HPV type identification and characterization of integrated HPV DNA using HC + SEQ

Since the breadth of effectiveness of GP5+/GP6+ primers with less common HPV types is uncertain^22^, and some HPV-induced cancers contain integrated, subgenomic HPV DNA segments lacking the GP5+/GP6+ target segment^12^, we performed HC + SEQ on all 13 samples to define the identity and structure of HPV DNA in each^21^. HPV DNA enrichment was accomplished through the utilization of a custom-designed capture probe set, consisting of oligonucleotide probes complementary to 143 different HPV types (HPV1 through HPV143^10^). This comprehensive probe set encompasses the high-risk types recognized by the World Health Organization (WHO) International Agency For Research on Cancer (IARC) as carcinogenic or probable carcinogenic (HPV-16, -18, -31, -33, -35, -39, -45, -51, -52, -56, -58, -59, -68, -69)^23^. Biotinylated DNA probes, each comprising overlapping, single-stranded sequences covering both DNA strands of the full ~8 kbp genomes of each HPV type, allowed capture along the entire viral genome of each sample if present. The paired-end sequencing reads obtained were aligned using STAR^24,25^ to a combined reference genome comprising the GRCh38/hg38 human genome assembly plus the sequences of all 143 HPV types included in the capture panel based on reference genomes in the Papillomavirus Episteme^10^.

**Fig. 1:**
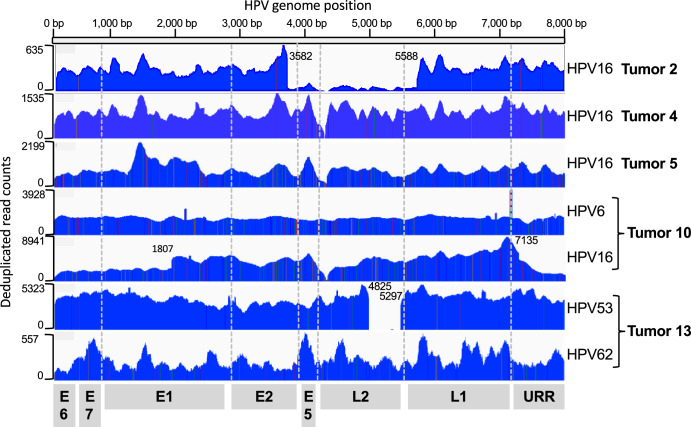
Coverage of HPV genomes and typing based on viral DNA hybridization capture plus Illumina short read sequencing. The horizontal scale depicts the linearized HPV genome, starting from E6 (positions 1 and 7906 in HPV16). The corresponding locations of viral open reading frames are displayed at the bottom. The plot depicts the deduplicated DNA sequence read counts for each sample, with the Y-axis scales on the left for each tumor. The identified HPV type(s) are indicated on the right side of the plot. The boundaries of underrepresented segments in Tumor 2 (3582–5588), Tumor 10 HPV16 component (URR-E6-E7 segment 7135 to 1805), and Tumor 13 HPV53 component (4825 to 5297) are indicated.

HC + SEQ detected HPV DNA in five samples (39%) the four that tested positive by PCR using the GP5+/GP6+ primers and one additional tumor (Tumor 13). Average HPV genome coverage by HC + SEQ ranged from 139X to 8414X (Supplementary Table 1). Alignment of sequence reads across the viral genome (Fig. 1) allowed unambiguous identification of the HPV types in each of the five tumors, illustrating an inherent strength of the HC + SEQ approach. Tumors 2, 4 and 5 yielded DNA exclusively from HPV16, the most prevalent of the high-risk HPVs in human cancers independent of tumor anatomical site. In tumor 2, HPV16 genome read coverage was sufficiently high to determine that the predominant HPV DNA comprised a segment of about three-fourths of the viral genome extending from a carboxyl terminal-encoding portion of L1, through the URR, E6, E7, E1, and into an amino terminal-encoding portion of E2, with the segment from reference genome positions 3582 to 5588 absent. In addition, coverage of full-length HPV16 DNA was also detected in this tumor, although at much lower levels, suggesting that only a small fraction of cells in the tumor contained the full-length HPV16 genome. Tumor 4 contained a near full-length sequence of HPV16 DNA, missing a 16 bp segment from reference genome positions 4162 to 4178 in the E5 gene. Tumor 5 yielded reads mapping to the complete HPV16 genome.

In contrast, Tumors 10 and 13 both contained two HPV types, with substantial DNA sequence read count coverage for each type, indicating that both patients had incurred co-infections (Fig. 1). Tumor 10 contained DNA covering the complete genomes of both HPV6, considered low risk for cervical cancer, and high-risk HPV16. Likewise, Tumor 13 contained two HPV types, HPV53 and HPV62, both relatively uncommon HPV types with neither currently categorized as group 1 carcinogenic or group 2 probable carcinogenic^23^. However, it is worth noting that HPV53 has been previously associated with cancer cases^26^. The HPV53 DNA comprised a subgenomic segment with loss of viral DNA sequence from positions 4825 to 5297 in the L2 gene, while the entire HPV62 genome was present. The number of read counts for HPV53 was about 16-fold higher than for HPV62 (Supplementary Table 1), suggesting that the former is likely more abundant, and that it was more likely to have had a carcinogenic role than HPV62. These data do not differentiate whether DNA from the two HPV types were within the same cells, or if each type existed in distinct, separate cells in the tumors. Nonetheless, the detection of abundant DNA of two relatively uncommon HPV types in a tumor that was positive by HC + SEQ but not by GP5+/GP6 + PCR illustrated the breadth of sensitivity of the contemporary HC + SEQ approach to detect HPV in tumors. In summary, HPV DNA was detected in five of the tumors, and in all these cases, the identified DNAs retained the upstream regulatory region (URR), as well as the open reading frames (ORFs) of E6 and E7 of the cognate HPV types.

### HPV DNA is integrated into the human genome in some vulvar cancers

**Fig. 2:**
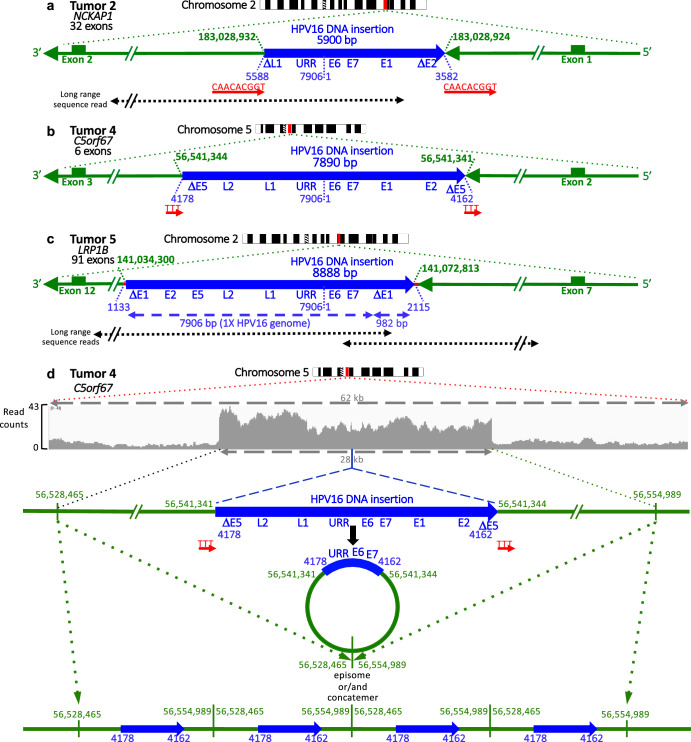
Structures of integrated HPV16 DNAs in Tumors 2, 4, and 5 based on HC + SEQ aligned at the URR-E6-E7 segment. For each tumor, the approximate position of the HPV DNA insertion (red bar) is shown on the cognate chromosome ideogram. The structure of each integrated DNA is represented as a thick blue arrow, with viral DNA segment length and the approximate positions of viral open reading frames (ORFs) and the upstream regulatory region (URR) also shown in blue. Flanking the viral DNA junctions, human genome segments are depicted as thinner green arrows, including the adjacent flanking exons shown as rectangles. Arrows indicate the 5’ to 3’ orientation of the plus strands of both viral and cellular genes, with the HPV16 DNAs integrated into the opposite transcriptional orientation compared to the human genes in each of the three tumors. The positions of the insertion junctions in the human and HPV reference genome (hg38 and PAVE, respectively) are indicated in green or blue, respectively. For Tumors 2 and 4, short, direct repeat, human DNA sequences immediately flanking the insertions are shown in red. In Tumor 5, segments of microhomology between the viral and human DNA at the junctions are depicted as a short red line. Below the genomes for Tumors 2 and 5, positions of long-range DNA sequence reads are represented by dotted black arrows. HPV and human DNAs were drawn at different scales. **a** Genetic diagram illustrating the structure of the HPV16 DNA segment integrated in intron 1 of the *NCKAP1* gene in Tumor 2, with the 9 bp, direct repeat sequence (5′-CAACACGGT-3′) immediately flanking both sides of the viral insertion in red. **b** Genetic diagram of the HPV16 segment in intron 2 of the *C5orf67* gene, where a 3 bp direct repeat sequence (5′-TTT-3′) is shown immediately flanking the viral insertion on both sides in red. **c** Genetic diagram of HPV16 DNA in Tumor 5 inserted between exons 7 and 12 in the *LRBP1* gene. The HPV16 genome was noted to be present in tandem with at least one full genome and an additional 982 bp, as determined by HC + SEQ and long-range MinION nanopore sequencing reads spanning this region. This integration event coincided with a 38 kbp deletion within the *LRBP1* gene. **d** Long-range MinION nanopore sequencing of Tumor 4, specifically showing the sequence read coverage for a 62 kbp segment from the second intron of C5orf67 on chromosome 5. The plot depicts the over-representation of a 28 kb segment containing the HPV16 DNA segment. Below the read count plot, a linear genetic diagram represents the inserted HPV16 DNA and the 28 kb stretch of high read counts. Circular episome and DNA concatemer structures are depicted below, each consistent with the sequencing results.

To determine whether HPV DNA was integrated into the human genome in the five HPV-positive vulvar tumors, the DNA sequence reads were computationally examined for split reads containing a junction between HPV and human DNAs using STAR^24,25^. Junctions were detected in Tumors 2, 4 and 5 (Fig. 2, junction sequences shown in Supplementary Fig. 2). In Tumor 2, HPV16 was integrated into the first intron of the *Nck-associated protein 1* gene (*NCKAP1)* gene on chromosome 2, with the viral genome oriented in the opposite transcriptional direction as *NCKAP1* (Fig. 2a). The HPV-human DNA junctions were at HPV positions 3582 and 5588, the precise base pairs at the ends of a high coverage segment in Fig. 1 and were joined to intron 1 of *NCKAP1*. Thus HC + SEQ showed that the predominant form of HPV16 in this tumor was an integrated, subgenomic viral DNA segment between those positions. Moreover, a 9 bp duplicated sequence (5′-CAACACGGT-3′) immediately flanked both sides of the viral insertion (Fig. 2a, Supplementary Fig. 2). In Tumor 4, HPV16 was integrated into chromosome 5 in the second intron and in the opposite transcriptional orientation of *Chromosome 5 Open Reading Frame 67* (*C5orf67)* (Fig. 2b), a gene of unknown molecular function. The viral DNA ends at the two junctions again occurred precisely at the ends of the deletion determined by HC + SEQ (16 bp, positions 4162 to 4178 in the E5 gene, Fig. 1). The insertion was flanked by a direct repeat of 3 bp (5′-TTT-3′) from the human genome (Fig. 2b, Supplementary Fig. 2). In Tumor 5, HPV16 DNA was integrated into the *LDL receptor-related protein 1B* (*LRP1B*) gene on chromosome 2 (Fig. 2C). The viral DNA was again in the opposite transcriptional orientation as the human gene, with the junctions in introns 11 and intron 8 of *LRP1B*, leading to the inference that integration was accompanied by a 38 kbp deletion between those introns including exons 9 through 11 (Fig. 2c). Each of the junctions had a short sequence identity (microhomology) between the cognate ends of the human and HPV16 genomes (1 bp or 8 bp, Supplementary Fig. 2), suggesting joining of viral and human DNAs by microhomology-mediated end joining^17^. Such microhomologies or insertions occur frequently at HPV DNA insertion junctions in cervical tumors^27^. Since the entire HPV16 genome was covered by sequence reads, we were able to infer that the inserted HPV16 DNA comprised a tandem structure longer than one 7906 bp viral genome (Fig. 2c). All HPV-human DNA junctions were confirmed by PCR on tumor DNAs (Supplementary Fig. 2).

To analyze the structures and junctions further, long-range DNA sequencing was also performed on genomic DNA from each of the three tumors with integrated HPV16 DNA. While prone to short sequence misreads and indels, this technique is effective for elucidating larger scale genomic structures and rearrangements. One long sequence read was obtained confirming the insertion in one allele of *NCKAP1* in Tumor 4 (Fig. 2a). Two reads were obtained for HPV16 in *LRP1B* of Tumor 5, which confirmed the structure of the inserted HPV16 DNA with the additional 982 bp (Fig. 2c).

For the HPV16 DNA insertion in *C5orf67* in Tumor 4, over 100 long-range sequencing reads were obtained covering the viral genome and nearby human sequences (Fig. 2d, Supplementary Fig. 3). Of these, 59 of them covered HPV16 sequences, some including one or both of the two junctions identified by Illumina sequencing, as well as some reads that covered the entire HPV16 DNA insertion plus flanking human DNA on both sides, thus confirming the overall structure and both junctions. Moreover, a 28 kbp segment of the human genome with the HPV16 DNA insertion site near the center had on average about 4- to 5-fold higher coverage than the flanking human DNA (Fig. 2d). This indicated that a segment comprising about 36 kbp of human plus HPV16 DNA (~26 and ~8 kbp, respectively) was amplified relative to nearby sequences on chromosome 5.

Since HPV DNA is sometimes present in episomal (extra-chromosomal, circular) form linked to human DNA in tumors^20,28,29^, we analyzed the long-range sequence reads at the two boundaries of the amplified, chromosome 5 segment to determine if they were joined (Supplementary Fig. 3). We identified 55 long-range reads in which the reference human genome (hg38) positions 56,528,445 and 56,554,989 were precisely continuous (Fig. 2d, Supplementary Fig. 3). This junction was consistent with either a 36 kbp episome or a concatemer of the identical 36 kbp sequence integrated in the human genome (Fig. 2d). The formation of such an episome could be explained by a multistep process. First the subgenomic segment of HPV16 integrated into one allele of *C5orf67*. After integration, the two sites in human DNA flanking either side of the viral DNA insertion rearranged and joined together to form a circle (Fig. 2d, Supplementary Fig. 3). Presumably, the HPV-human hetero-chimeric episome then attained an average 4- to 5-fold level of amplification by DNA replication processes, possibly involving HPV-specific replication mechanisms^20,28,29^. However, the sequencing data do not exclude the possibility that the 36 kbp hetero-catemeric segment is oligomerized as tandem, 36 kbp, direct repeats in a larger episome, nor the possibility that the 36 kbp hetero-catemers might be present as tandem repeats that are integrated with the human genome (Fig. 2d), or even that both episomes and integrated tandem repeats might present in the tumor. Hetero-catemers may occur frequently in HPV-induced tumors^20,28–30^. While it can be argued that episomal hetero-catemers are not integrated into the human genome per se, the HPV segments in them are each joined by two junctions with the human genome just like an integration event in a human chromosome, and arguably derived from such events. The long-range sequencing additionally showed that the normal, “empty” allele of *C5orf67* without the HPV16 DNA insertion was also present in the tumor. This analysis emphasizes the strength of the HC + SEQ and long-range sequencing approaches to elucidate the detailed, large-scale structures of integrated HPV DNAs including human-HPV16 DNA hetero-catemeric structures.

To summarize, Tumors 2, 4, and 5 exhibited the presence of integrated HPV16 DNAs. In Tumors 2 and 4, subgenomic segments of HPV 16 DNA were identified, while in Tumor 5, a tandem array comprising at least one full-length HPV16 genome was observed. The insertion in Tumor 4 was a hetero-concatemeric, 36 kbp episome and/or an integrated tandem repeat that was chimeric with human DNA. Two of the three inserted viral DNAs were flanked by short direct repeats. The viral DNAs all included an intact segment with the URR regulatory sequences upstream of the E6 and E7 genes as appropriate for transcription from the viral early promoter, emphasizing the importance of these viral genome components for tumorigenesis. In contrast, the late genes were situated upstream of the URR and HPV transcription start sites, and thus improperly positioned to be transcribed from the viral URR or late (in E7) promoters. Such an overall structure is characteristic of HPV DNA integrations in other HPV induced tumors^21,31^, and presumably is important for expression of the viral E6 and E7 oncogenes.

While co-infections were detected in both Tumors 10 and 13, no human-viral DNA junction sequence reads were detected. Viral DNA presence was confirmed by PCR in both tumors using primers designed for E6 or E7 components of the specific virus (Supplementary Fig. 4). In Tumor 10, there was full coverage for both HPV6 and HPV16. Oddly, reads were detected crossing an intraviral DNA junction between nucleotides 1807 (within E1) and 7135 (within L1) of HPV16 (Fig. 1). The segment extending from L1 through the URR, E6, E7 and into E1 had about 40% as many reads as rest of viral genome, suggesting that, in addition to full-length HPV16, an unusual, unintegrated, subgenomic segment from 1807 through 7135 was somehow present in at least a fraction of the cells in the tumor. Tumor 13 showed the full genome of HPV62, while the HPV53 genome had the intraviral genome with a deletion from nucleotides 4825 to 5297 (Fig. 1). The presence of deleted forms of HPV in the tumors at various levels is consistent with the ideas that the viral DNA is unstable in tumors, and the absence of junctions between any of the HPVs in Tumors 10 and 13 with human DNA might suggest that these viral DNAs were episomal rather than integrated into the human genome, or perhaps that the sampled portion of the tumor lacked integrated DNA.

### In vulvar tumors with integrated HPV DNA, viral RNA transcripts are limited to the early genes and predominantly encode full-length E7 and the E6*I spliced isoform open reading frames

**Fig. 3:**
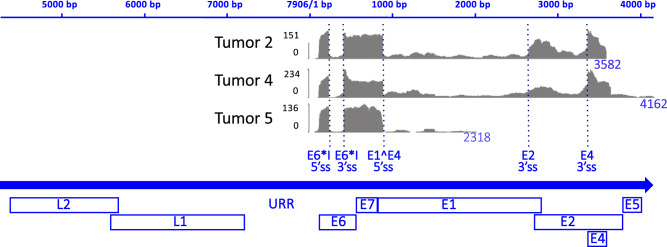
RNAseq analysis of HPV transcripts in three vulvar tumors harboring integrated HPV16 DNA. The plots depict read counts aligned to the HPV16 genome using IGV, with the Y-axis representing the scale of read counts for each plot, and read counts displayed as counts per million reads. At the bottom, the full-length HPV16 genome is shown, indicating the positions of the viral open reading frames (ORFs) represented as boxes. The large blue arrow indicates the 5’ to 3’ transcriptional orientation of all the viral ORFs. The standard numbering scale for the HPV16 genome is shown at the top. The genome is linearized between the early (E) and late (L) ORFs to position the URR-E6-E7 segment centrally as is characteristic of integrated HPV genomes in tumors (see Fig. 2) including the URR immediately upstream of the transcribed E6 and E7 ORFs. The viral nucleotide immediately preceding the junction with human DNA is numbered at the end of each plot. HPV RNA 5′ and 3′ splice sites (5′ss and 3′ss) are labeled and indicated by the vertical dashed lines. These include the E6*I splice sites, responsible for a frame-shifting deletion of much of the E6 coding segment, and the 5′ss (E1^E4 5’ss) just downstream of the E1 start codon that typically splices to the 3’ss for the E4 ORF, and also can join to 3’ splice sites in the human genome downstream of inserted HPV DNAs. No transcripts from the viral L regions were detected in any of the tumors.

Based on RNA-sequencing (RNA-seq) analyses of bulk tumor tissue, viral transcripts were found to encompass only a fraction of the HPV transcriptome. RNA-seq reads were aligned to their respective HPV type reference genomes from PAVE^10^ using STAR v2.7.9a^25^. Coverage patterns (Fig. 3) in each of these tumors reflected mRNA transcripts derived from the early promoter, encompassing E6, E7, and the very beginning of E1, with viral genome coverage extending precisely to the positions where viral DNA was joined to human DNA. There was extensive use of viral splice junctions. Notably, almost all transcripts from the E6 gene had the E6*I intron excised in all three tumors (Fig. 3). This small intron spans 182 nucleotides from positions 227 to 408 within the E6 ORF. Excision of it results in mRNAs encoding a frameshift at the splice junction that results in a C-terminally truncated form of E6 protein. The tumors all contained levels of full-length E6 open reading frame (ORF)-containing mRNAs that were far lower than those for the E6*I and E7 ORFs. There was also extensive use of the 5’ splice site (5’ss) in E1 in all three tumors, and 3’ splice sites (3’ss) for E4 in Tumors 2 and 4. For Tumor 5, the only viral transcripts detected downstream of the E1 5’ss were a low level of transcripts from the E1 region, with no transcripts from E2, E4, and E5 identified, as expected from the integrated DNA structure. Likewise, no transcripts from the disrupted E5 region were detected in Tumor 2. Tumor 4 did contain an intact E5 ORF in the inserted HPV16 DNA (Fig. 2). However, part of the 3’ untranslated region was truncated by the insertion junction at position 4136 situated 35 bp downstream of the E5 ORF stop codon and upstream of the canonical AATAAA polyadenylation signal, and thus, predictably, only few E5 transcripts were detected in that tumor. The quality of RNAs obtained from HPV DNA-containing Tumors 10 and 13 was too low to yield RNA-seq data.

**Fig. 4:**
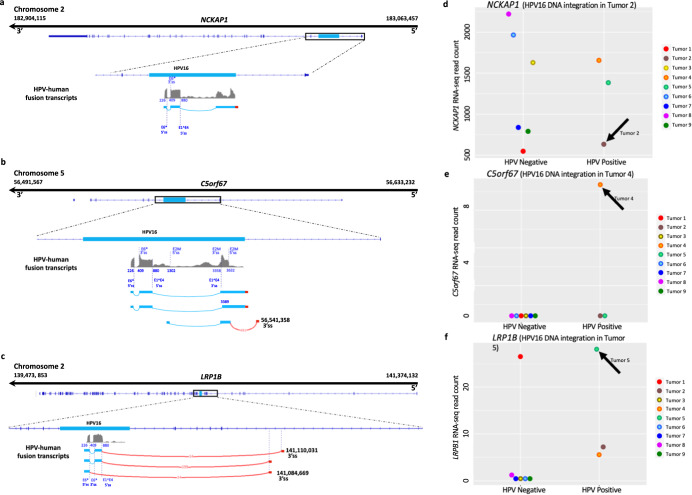
Transcription of human genes with HPV16 DNA insertions determined from RNAseq data. **a**, **b**, **c** Positions of human RNA sequences derived from the antisense strand of the cognate human genes (brown) fused with HPV16 RNA sequences (blue) for each tumor. The top line in each displays the chromosomal position and 5′ to 3′ transcriptional orientation of the human gene. Below that, IGV plots show the exon-intron structure of human gene, with the HPV16 DNA insert displayed in a lighter shade of blue. The boxed regions within each gene highlight the segments containing HPV16 DNA, with spliced fusion transcripts shown below the DNA for detailed visualization of viral genetic detail. The scales of the HPV16 DNA are larger than the flanking human DNA segments to allow transcriptional features to be presented. The brown arcs represent the introns excised from HPV16 5’ splice sites (5’ss) to presumably cryptic 3’ splice sites (3′ss) of the human genes. The numbers within the arcs indicate the RNAseq read counts for each splice site junction. The majority of the transcripts derived from HPV16 exhibited splicing at the major E6* splice junction known as E6*I (blue arcs), with the positions of the junctions at nucleotides 226 and 409 shown in blue. Most splicing from HPV sequences to human gene antisense sequences involved the 5’ss at position 880 at the beginning of the viral E1 gene open reading frame as shown. For Tumor 5 (**c**), a smaller number of spliced RNAs alternatively involved the 5’ss at position 226 within the E6 gene, which joined a 3′ss of *LRP1B*. In Tumor 4, numerous transcripts were spliced from HPV16 sequences originating at the viral late promoter and entailed the 5’ss at position 1302 labeled E2M. **d**, **e** and **f** show the total numbers of RNAseq reads derived from the sense strands of the indicated human genes, aggregated for each gene. The total deduplicated read counts (counts per million reads) from each human gene are plotted separately for the HPV-positive and the HPV-negative vulvar cancers. Specifically, for tumors 1–9. Each panel utilizes a distinct Y-axis read count scale for the corresponding sample. The black arrows indicate samples in which HPV16 DNA was integrated into the specific human gene.

Transcription from each of the three integrated HPV DNAs extended from viral sequences into the human sequences (Fig. 4a–c). In all three instances, the transcribed human segment derived from the antisense strand of the intron of the human gene in which the viral DNA was inserted as the template, with the length of the human portion of the transcripts varying among the three insertions. In some instances, the transcripts comprising the sequences immediately downstream of the inserted viral DNA were sufficiently stable to be detected. In others, the human sequences were joined by presumably cryptic 3’ss’s to the upstream viral RNA by a splice junction, principally the E1^E4 5’ss (that splice site is indicated in Fig. 4a–c). The human portions of such transcripts were antisense to intronic sequences of the human genes in which the viral DNAs were integrated. Levels of spliced mRNAs (the positive sense transcripts) for each of the three human genes were assessed in the RNA-seq dataset (Fig. 4d–f). Transcripts were quantified and normalized for total read counts using Salmon^32^ prior to differential gene expression (DGE) analysis. All three human genes were expressed at only relatively low levels with *NCKAP1* the highest of the three, suggesting that the HPV URR enhancer had no substantive effects on expression of these human genes.

### Differential expression analysis of global human gene transcription between HPV-positive and negative VSCCs

To investigate whether the global transcriptome of HPV-positive vulva cancers differed from that of HPV-negative ones, human genome-wide transcriptomic analysis was performed on tumors with an RNA integrity number (RIN) above 6. The normalized sequencing read counts of nine tumors, three HPV positive (Tumors 2, 4, 5) and six HPV negative (Tumors 1, 3, 6, 7, 8, 9), were subjected to DGE analysis. Due to the small sample size, and to prevent one sample from dominating the differential results, we required at least three samples in any comparative group to have ≥10 normalized counts for any individual gene. Following prefiltering of genes with no counts, we found 16,532 genes out of 37,951 total normalized genes to fit these criteria. First to compare the HPV-positive and negative samples, principal component analysis (PCA) was performed on the regularized, logarithm-transformed counts of RNA-seq reads^33^. PC1 and PC2 accounted for 46.5% of the variability. HPV status was substantially represented in the first principal component (Supplementary Fig. 5A-C). Additional biological covariates assessed included whether the tumor was primary or recurrent disease, and if the patient received prior chemotherapy or radiation therapy. These covariates were represented primarily in PC5, and both were subsequently included in the linear model for DGE analysis. Hierarchical clustering of the 50 most variable genes showed that Tumors 2 and 4, two primary HPV-positive tumors, clustered together (Supplementary Fig. 5D). Curiously, over half of the 50 most variably expressed genes identified are involved in adaptive immune system function including human leukocyte antigen (HLA) genes and immunoglobulin genes.

To analyze differential gene expression patterns of HPV positive versus negative tumors further, we set a false discovery rate (FDR) of <0.1 and found 402 genes (2.4%) to be upregulated and 327 (2%) to be downregulated in HPV-positive tumors compared to HPV-negative tumors (Supplementary Fig. 5E and Supplementary Table 2). The top 10 differentially expressed genes by adjusted p-value were *MSH5*, *STAG3*, *RNF212*, *SYCP2*, *MBOAT7*, *TBC1D3*, *PORCN*, *DHX16*, *ZFR2*, and *DES* (Supplementary Fig. 6). Interestingly, four of these genes (*STAG3*, *RNF212*, *SYCP2*, *ZFR2*) were previously observed to be differentially expressed in other types of HPV-induced cancers^34–37^, indicating similarities among these virally-induced tumors. GO categorization and over-representation indicates that in addition to the significant enrichment of cell surface proteins essential for the adaptive immune system, HPV-positive vulvar tumors were associated with the activation of programs of ECM remodeling as reported for other HPV associated tumors (Supplementary Fig. 7)^38–40^.

**Fig. 5:**
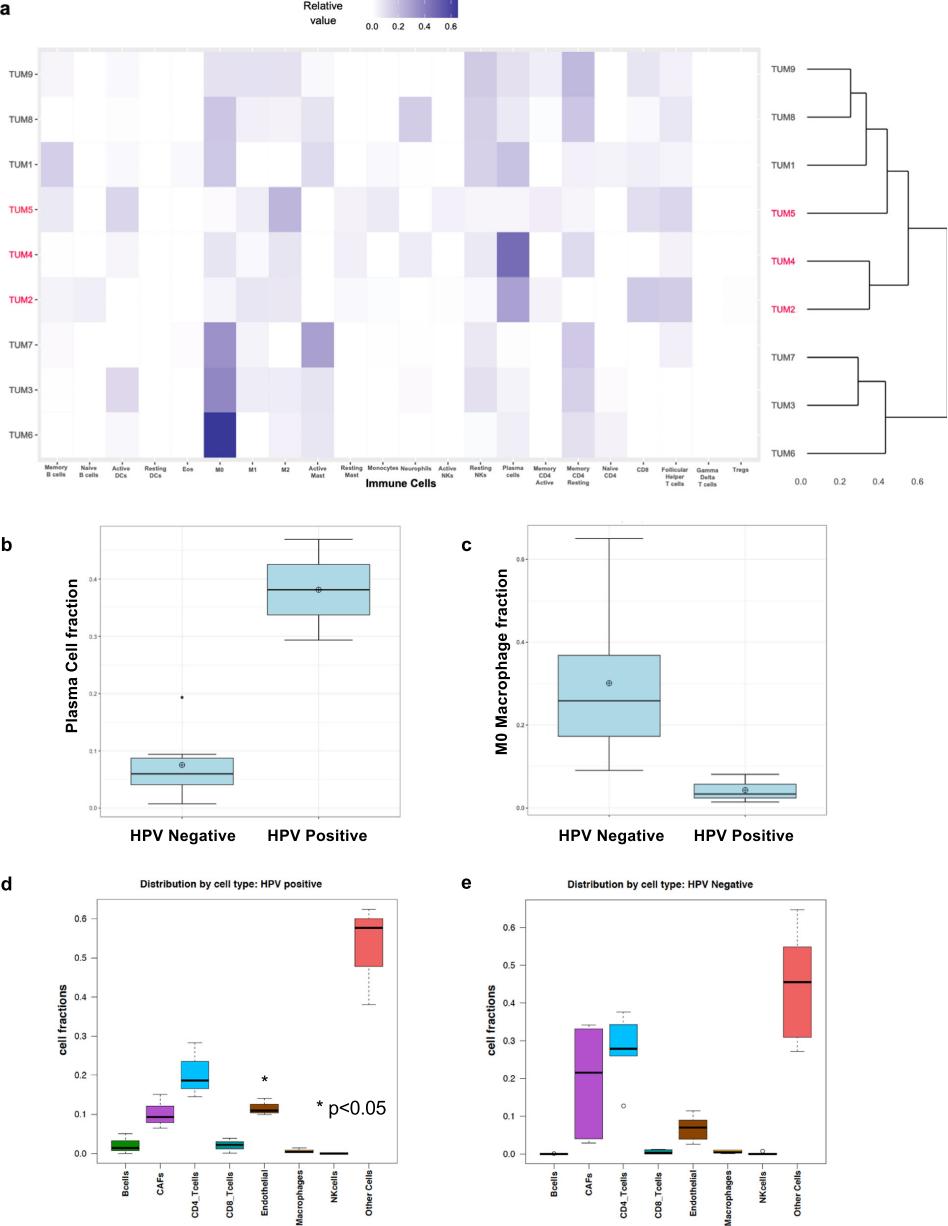
Evaluation of tumor infiltrating immune cells using RNAseq expression data. **a** The relative frequencies of 22 immune cell gene expression profiles across nine tumor samples were assessed using CIBERSORTx. HPV-positive tumors, identified through hybridization capture analysis, are labeled in red, while HPV negative tumors are labeled in black. On top of the figure a color-coded key to relative values. Tumors 2 and 4, both HPV-positive, exhibited relatively higher proportions of plasma cells in the tumor microenvironment. In addition, all three HPV-positive tumors displayed lower relative levels of M0 macrophages compared to HPV-negative tumors. EPIC was employed to further examine the tumor microenvironment and provide a condensed representation of immune cell populations in the HPV-positive and HPV-negative samples. Differences in plasma cells (**b**) and M0 macrophages (**c**) were observed. The tumor microenvironment was further evaluated using EPIC to provide a condensed set of immune cell populations in addition to cancer-associated fibroblasts and endothelial cells between HPV-positive (**d**) and HPV-negative tumors (**e**). Differences in (**b**) cells as a population whole did not meet statistical significance. However endothelial cells were noted to be more prevalent in HPV positive tumor’s TME compared to HPV-negative tumors (*p* = 0.04).

Because several markers pointing to adaptive immune response emerged as the most variable genes overrepresented in the HPV positive samples, we applied the cell subtype deconvolution algorithm CIBERSORTx^41^ to estimate the relative proportions of immune cell subtypes in the nine vulvar carcinoma samples based on bulk tumor gene expression profiling. Despite the inherent variability attributed to the small sample size, our analysis revealed intriguing findings in relation to the immune subtypes. Notably, hierarchical clustering analysis demonstrated distinct immune gene signatures between HPV-positive and HPV-negative tumors. This observation implies that, despite variations in immune subtype abundances, HPV-positive and HPV-negative tumors possess discernable functional properties that characterize them, thereby providing valuable insights into the underlying biological mechanisms involved (Fig. 5a). In HPV-positive Tumors 2 and 4, there was a relative enrichment of plasma cells, albeit not at statistical significance (Wilcoxon *p* = 0.06) (Fig. 5b). Tumor 5, which was also HPV-positive, did not show this enrichment, however this sample was taken from a disease recurrence site, and in addition, the patient had received both prior chemotherapy and radiation therapy, unlike Tumors 2 and 4 which were primary disease tissue biopsies from treatment naïve individuals. An additional pattern noted was that several HPV-negative tumors (Tumors 1, 3, 6, 7, 8) exhibited relative enrichment of non-activated M0 macrophages (Wilcoxon *p* = 0.02) (Fig. 5c). EPIC^42^ was also used to estimate tumor infiltrating cells in the HPV-positive versus negative tumors including stromal cells and endothelial cells in addition to condensed sets of immune cells (Fig. 5d, e). While the HPV-positive and HPV-negative vulvar cancers yielded similar infiltrating cell profiles, some differences were noted. The endothelial cell fraction was significantly higher in the HPV-positive compared to the HPV-negative tumors (*p* = 0.04). Furthermore, it was observed that the HPV-positive group of Tumors 2, 4 and 5 exhibited high overall levels of B cells. Although this did not reach statistical significance, perhaps it reflected the elevated plasma cell subsets observed in Tumors 2 and 4 with CIBERSORTx (Fig. 5a). Although statistical significance was not achieved, this finding is concordant with the observations obtained using CIBERSORTx. This finding may provide insight into the potential relationship between elevated B cell levels and the presence of plasma cell subsets in these HPV-positive tumors.

### Comparison of HPV-positive and HPV-negative vulvar cancers by whole genome sequencing (WGS)

Molecular genetic information about vulvar cancers is limited. In addition to the insights provided here about HPV-positive tumors, it was of particular interest to examine HPV-negative vulvar cancers and compare them to HPV-positive tumors. Therefore, we undertook whole genome, short-range, Illumina sequencing on all 13 tumor samples in this study. WGS analysis of the HPV positive (*n* = 5) and negative (*n* = 8) found on average similar frequencies of single nucleotide variants (SNVs), insertion-deletions (InDels), copy number variants (CNVs), and structural variants (SVs) per tumor in the positive and negative tumors.

The mean numbers of total SNVs, stop-loss SNVs, and missense SNVs were slightly higher in the HPV-positive tumors than the negative ones (nominal *p* < 0.05, t-test), but the means for stop-gain SNVs were not significantly different (*p* = 0.15). The numbers of affected genes with a stop gain or stop loss SNV mutation in all HPV-positive and HPV-negative tumors were 2893 and 2210, respectively. However, the SNV frequencies became similar when individual affected genes in at least 75% of the tumors were considered, with 88 affected genes in at least four of the five HPV positives and 83 in six of the eight HPV negatives. TP53 had a stop gain mutation in 1 HPV-positive tumor (Tumor 10) and 2 HPV-negative tumors (Tumor 8 and Tumor 9). One interesting finding was that every one of the 13 tumors had at least one single nucleotide, amino acid substitution mutation, independently of HPV status.

No statistically significant difference in InDel frequency between the two groups of tumors was detected (*p* = 0.06 for total InDels, t-test). For InDels that were mostly likely to cause functional disruptions (frameshifting deletions or insertions), the numbers of affected genes in all tumors were 47 and 29, in the HPV-positive and HPV-negative tumors, respectively. Again, however, the numbers became similar between the two groups when only individual genes affected in at least 75% of the tumors were considered, 88 in four of the five HPV-positive and 83 in six of the eight HPV-negative tumors.

Examination of SVs showed that 1347 and 779 genes were associated with SVs (duplication, deletion, or inversion) in at least four of the HPV-positive and six of the HPV-negative tumors, respectively. Some noticeable genes with SVs were EGFR (three tumors in HPV positive and one tumor in HPV negative group), CCND1 (three tumors in each group), and PTEN (one in each group).

Similarly, 47 and 45 genes were linked to CNVs (gain or loss) in at least four of the HPV-positive and six of the HPV-negative tumors, respectively. Some known tumor related genes were found in the CNV analysis, including *PIK3CA* in one HPV-positive (Tumor 2) and two HPV-negative tumors (Tumor 1 and Tumor 9), *TP53* in one HPV-positive (Tumor 5) and one HPV-negative tumor (Tumor 1).

In summary, the WGS analyses did not reveal any noteworthy differences between the HPV-positive and HPV-negative vulvar cancers in this study.

### Detection of novel amplified DNA in tumor 4

Analysis of the long-range sequencing and WGS data revealed additional rearrangements involving the DNAs present in the hetero-catemer of Tumor 4. Specifically, two structures with parts of the hetero-catemer deleted were detected as was a structure with just the HPV16 sequences lacking the sequences from nucleotides 4162 to 4178, as is the case in the integrated viral DNA in this tumor. These structures were also confirmed by alignment of WGS reads to the human and HPV16 genomes, and further verified using Amplicon Architect^43^. In addition to the HPV-human DNA hetero-catemer identified by long-range sequencing (Fig. 2d), these approaches also confirmed three structures present as potential episomes was obtained (Fig. 7), with two containing only human DNA surrounding the integration site of HPV16 DNA in the *C5orf67* gene of this tumor, and the third contained only HPV16 DNA. These observations suggest that HPV DNAs along with human sequences surrounding the integration sites may be subject to structural rearrangements at least in some tumors.

### Visualization of multiple, HPV16 DNA, integration site structures in single cells using DNA fluorescent in situ hybridization (FISH) analysis

To discern whether the HPV-human DNA hetero-catemer in Tumor 4 was present as an episome or as chromosomally inserted, tandem repeats (Fig. 2d), we examined the long-range DNA sequencing reads for direct evidence of the latter. If the hetero-catemeric DNA was integrated into chromosomal DNA, then an individual long-range sequence read might extend from the HPV sequences and past the human-human DNA junction between nucleotides 56,554,989 and 56,528,465 (Fig. 2d). No reads were identified that fit that criterion, and thus that approach was inconclusive.

Therefore, we devised a customized, single cell, multi-color FISH analysis using cryo-sections of Tumor 4 to distinguish episomal and chromosomally integrated HPV16 DNA (Fig. 8). This analysis detected variable HPV16 and chromosomal DNA content among the tumor cells. Two cells exhibiting key features from this analysis are shown. Fluorescent hybridization probes from HPV16 and from a BAC encompassing the integration site on chromosome 5 (Fig. 8a, b) were hybridized along with probes for chromosomes 3 and 7 (Fig. 8b). The probes were all validated by hybridization to metaphase and interphase nuclei of a cultured HNSCC line UM-SCC-47 containing HPV16 DNA integrated into a locus on chromosome 3 (Fig. 8c). One Tumor 4 cell is shown in Fig. 8d–h, and quantitatively analyzed in Fig. 8i–k. The second cell is presented in Fig. 8l–s. Hybridization signals were quantified using the BioView scanning system as described in the Methods section.

The first cell was chosen as an example containing: 1) episomal HPV16 DNA, 2) chromosomally integrated hetero-catemeric DNA, and 3) episomal human DNA from the integration locus on chromosome 5. Panel 8D indicated that this specific cell was tetraploid. Panels 8E to 8G depict two copies of chromosome 5 with integrations of HPV16 DNA and three without it. Those with integrations (Fig. 8h panels1 and 2) were further amplified as shown by a brighter signal, with one distinctly exhibiting at least 3 visible tandem repeats (Fig. 8h panel 2). Notably, in addition, numerous small area, low intensity signals were also observed (Fig. 8i–k). Many of these gave signals from only HPV16 DNA or only the *C5orf67* locus on chromosome 5. These signals were precisely consistent with amplified episomal forms of either HPV16 DNA or the *C5orf67* locus (Fig. 7). Moreover, the HPV16 DNA signals were more numerous than the chromosome 5 signals (Fig. 8i). We propose that these signals derived from the amplified human-DNA-only and HPV-DNA-only episomes shown in Fig. 7, and that the FISH analysis detected these structures.

The second cell, which is depicted in Fig. 8l–s, highlights a quantitively distinct pattern of chromosomally-associated and episomal forms. This diploid cell exhibited only a single locus of HPV16 DNA integrated into the *C5orf67* locus of chromosome 5 (Fig. 8o, p). There was a large number of human-DNA-only signals of variable intensity that may have arisen from episomes or tandem forms of the *C5orf67* locus, and these lacked HPV16 DNA. Moreover, they were clustered to some extent within the nuclei of the cell. We emphasize that all the types of structures shown in the two examples were representative of types present in multiple, though not all, of the tumor cells.

In summary, we present evidence based on single cell FISH analysis of the likely presence of chromosomally inserted, HPV-human DNA hetero-catemers. We also unexpectedly detected the presence of amplified, human-only, presumably episomal DNAs from the integration locus on chromosome 5 in both cells examined, and even evidence for episomal HPV16 DNA in one of the two cells. Therefore, based on just these two cells, there was clear evidence of a multiple structural forms of HPV16 DNA and its chromosome 5 integration site in this tumor consistent with the amplified structures detected by WGS.

## Discussion

Using cutting-edge sequencing methodologies to analyze both human genome integration of HPV DNA in a fraction of vulvar tumors plus viral and human gene transcription revealed detailed new insights about vulvar cancers.

The detection of HPV DNA in ~40% (5 out of 13) of VSCC’s aligns with the reported range of HPV positivity observed in other studies investigating vulvar cancer^3^. Furthermore, the exclusive association of HPV with VSCC and not lichen sclerosis is consistent with previous reports^3^. The presence of HPV16 DNA in four out of five HPV-positive tumors is consistent with the predominance of this high-risk HPV type in vulvar cancers and other HPV-induced malignancies^13^. Additionally, despite the predominance of HPV16, the broad specificity and sensitivity of the HC + SEQ approach enabled the detection of DNA from less common HPV types, specifically HPV53 and HPV62 in Tumor 13, thus showing the strength of this unbiased approach for assessing HPV prevalence accurately. Co-infections were detected in two of the five HPV-positive tumors (40%). This is similar to a study that utilized GP5/GP6 primers for the detection of HPV and found that 6/16 HPV-positive vulvar tumors examined contained DNA of multiple HPV types^44^. In both the co-infected tumors characterized here, a combination of one elevated-risk and one common cutaneous type was observed. Large population studies of cervical cancer suggested that co-infection with high-risk and non-high risk HPV types was associated with a reduced risk of future invasive disease compared with hrHPV alone^45^. The breadth of low-risk HPV type detection is only afforded by HC + SEQ using capture probes from a large number of HPV types, which, at this time, is applied only in research settings. Therefore, the true extent of co-infections and their association with clinical outcome remain largely unknown. In light of current research aimed at developing immune- or genetic-based therapies that target specific HPV types^46–49^, the broad type specificity of HC + SEQ may be advantageous for identifying the HPV types present in tumor cells of any HPV-induced cancer including VSCC.

To our knowledge, only one study has previously reported HPV integration in vulvar tumors^50^ and reported integration events in 8 out of 55 samples (3.8% of a cohort in which 8 samples were found HPV positive). We detected viral DNA integration into the human genome in three (60%) of the five HPV-positive samples (Fig. 2). The presence of specific viral DNA segments joined at specific sites in the human genome and their detection by multiple junction sequence reads indicated clonal expansion of the cell in which the integration event originally occurred. The absence of integrated DNA in two of the tumors (10 and 13) is unlikely to be attributed to a failure in detecting HPV-human DNA junctions by HC + SEQ, considering the comparable high number of viral DNA reads in these two samples compared to tumors 2, 4, and 5. Furthermore, RNA transcripts of E7 genes were detected in both tumors 10 and 13 by reverse transcriptase PCR (data not shown), suggesting a role for the encoded oncogenic viral proteins. This observation aligns with the findings of ref. ^50^ who also reported lack of integration in one of the HPV-positive vulva tumors. These collective findings highlight the need for further studies to better comprehend the implications of the absence of HPV integration at advanced stages of tumorigenesis in a fraction of HPV-positive vulva tumor samples.

**Fig. 6:**
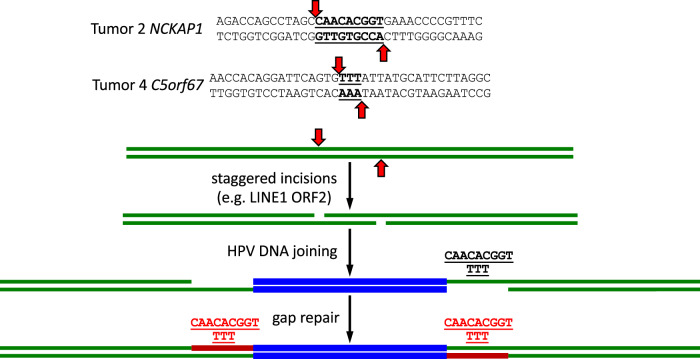
Proposed mechanism for the insertion of HPV16 DNA segments following a staggered incision on double-stranded DNA such as those by LINE1 element ORF2 protein. Double-stranded, human genome sequences for the empty, pre-integration alleles in Tumors 4 and 5 are shown at the top. Block vertical arrows show positions of incisions on each strand. Successive steps of staggered cleavage in human genomic DNA (green), viral DNA joining (blue), and gap repair (brown) are shown below. This process results in duplication of the human sequences between the incisions into direct repeats that immediately flank the inserted viral DNA. The viral DNA is shown as blunt-ended, but the mechanism can also apply with overhanging ends. The T:A base pair two nucleotides to the right of the TTT in the Tumor 5 C5orf67 sequence is an A:T base pair in the reference human genome (hg38).

Curiously, the viral DNA insertions in Tumors 2 and 4 were flanked by short direct repeats with no deletions or insertions of the human genome at the integration sites (Fig. 2). HPV DNA insertions usually occur in conjunction with deletion of human sequences between the human-virus DNA junction pair, and with microhomology or small DNA insertions at the junctions with no flanking repeats^21^, similar to the observations made for Tumor 5 in this study (Fig. 2). In contrast, the HPV DNA insertions in Tumors 2 and 4 were consistent with abortive, staggered, endonucleolytic incisions on the two strands of human DNA followed by joining of linear viral DNA and repair of the resulting single strand gaps that created the short flanking duplications without deletion of any human sequences (Fig. 6). While such staggered cuts theoretically could be due to various DNA-breaking mechanisms, short flanking direct repeats are canonical at the sites of retrotransposable element and retrovirus DNA insertions. Therefore, we speculate that the structures observed in Tumors 2 and 4 might reflect an HPV DNA integration mechanism involving human LINE1 element ORF2 protein in these vulvar tumors, which cleaves target site DNA at staggered positions on the two strands separated, unlike retrovirus endonucleases, by varying numbers of base pairs^51,52^. LINE1 elements are widely and frequently activated in human cancers^53^. It was previously noted that HPV E7 and E6 expression may induce LINE1 element expression, and that viral deregulation of *APOBEC3* may protect virally infected cells against detrimental effects of LINE1 activation^54^.

The structures of the integrated HPV16 genomes in vulvar tumors 2, 4, and 5 (Fig. 2) were similar to those reported for cervical cancers and HNSCCs^21,55,56^. Two were subgenomic segments of viral DNA, and the third was a tandem repeat. Most significantly, the inserted HPV DNAs contained intact URR-E6-E7 segments, consistent with the importance of these viral genes and their transcriptional regulatory sequences in tumorigenesis^57^.

**Fig. 7:**
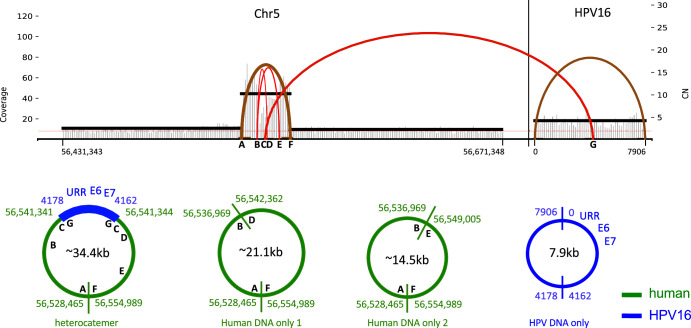
Multiple DNAs detected by nanopore DNA sequencing and whole genome sequencing (WGS). Top: Output from Amplicon Architect to verify the structures detected by long-range nanopore DNA sequencing. Read counts from WGS are plotted on a segment of chromosome 5 on the left and the HPV16 genome on the right. “Coverage” on the Y-axis indicates read counts, and “CN” on the Y-axis indicates estimated copy number. Each arc above the plots shows a junction between two non-contiguous positions in the human genome or between the human and HPV16 genomes. The letters A–G represent segments in the human or HPV genomes surrounding the positions of junctions between two non-contiguous positions as detected by nanopore and WGS. Note that C and G each correspond to the two distinct HPV-human DNA junctions separated by only very short distances in the human or HPV16 genomes as depicted in Fig. 2d (human 56,541,341 to HPV16 4178 and human 56,541,344 to HPV16 4162), and in the circle on the left of the lower row in this figure. Bottom: The four circles in the lower row indicate the amplified elements as episomes that were detected by DNA sequencing including Amplicon Architect analysis of WGS data and alignments of sequence data with the human and HPV16 genomes. Junctions are indicated by juxtaposed pairs of letters A through G, specifically A–F, B–D, and B–E involving human to human DNA junctions, and the C-G junctions depicting the two human to HPV junctions. These were the only human-HPV DNA junctions detected. The circle on the left is labeled as “hetero-catemer” and is precisely identical to that determined from long-range sequencing data as illustrated in Fig. 2d. The two structures in the center (labeled “human DNA only 1” and “human DNA only 2”) contain only human sequences including the A–F junction of non-contiguous sequences and only part of the sequences in the hetero-catemer and included no HPV sequences. The structure on the right comprises HPV DNA only. The size of each sequence in the bottom row is shown in the center of each circle.

**Fig. 8:**
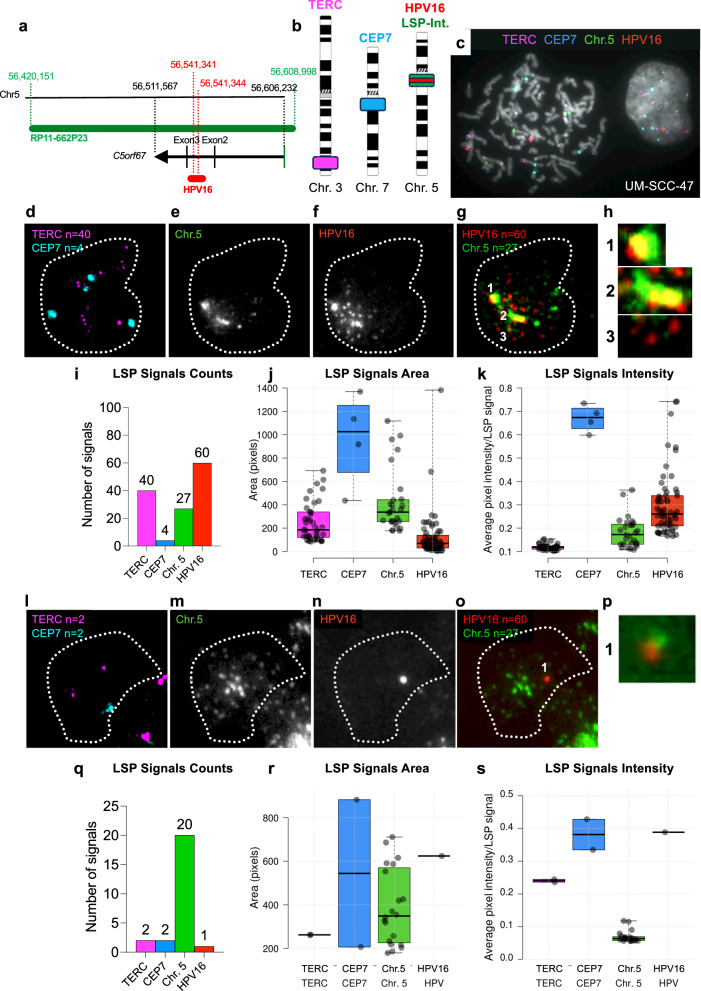
Visualization of HPV16 DNA and human chromosomes by custom DNA FISH in vulva Tumor 4. **a** Schematic representation of HPV16 DNA inserted in human chromosome 5 (hg38) and custom DNA FISH probes designed to visualize viral insertion in single nuclei. The black line at the top represents the hg38 human reference genome. Green denotes the relative mapping of BAC clone RP11-662P23 to the human reference genome. Red indicates the position of HPV16 DNA in the *C5orf67* locus relative to the human genome and to BAC clone RP11-662P23. The black line at the bottom shows the direction of transcription of *C5orf67* and the position of the HPV16 DNA relative to the *C5orf67*, 20 kb, second intron between exons 2 and 3 within RP11-662P23. Relative sizes of the human genome, BAC clone RP11-662P23 and HPV16 are drawn to scale. **b** Chromosomal positions of locus-specific probes (LSP’s) to visualize the *TERC* locus (magenta) on chromosome 3, the centromere of chromosome 7 (CEP7 in aqua), and the chromosome 5 insertion site (green) of HPV16 DNA (red) used for Tumor 4. **c** Validation of FISH probes using the UM-SCC-47, an HNSCC cell line with HPV16 DNA inserted into human chromosome 3 about 20 Mb from the *TERC* gene. A metaphase chromosome spread is shown on the left, and an interphase nucleus is depicted on the right. Gray scale depicts an inverted DAPI. Pseudo-colors of LSP signals correspond to the respective loci as indicated in (**b**). A representative nucleus from Tumor 4 is delineated by the dotted white line. Signals for the indicated probes as detected by the different fluorophores are shown as follows: **d** LSP depicting chromosome copy number for *TERC* and CEP7; **e** LSP depicting copies of the chromosome 5, HPV16 DNA integration site at *C5orf67* mapped by HC + SEQ; **f** LSP depicting HPV16 DNA; **g** Color-coded, merged image of the chromosome 5 insertion locus and HPV16 DNA, with yellow indicating signal overlap. **h** Zoomed-in images enlarged from (**g**) of integrated HPV16 DNA in panels 1 and 2, and non-integrated HPV16 DNA in panel 3. **i** Bar graph depicting the number of LSP signals identified in the nucleus shown in (**d–g**). Box plots depicting the area (**j**) and intensity (**k**) of each LSP signal identified in the Tumor 4 nucleus in (**d–g**). Center lines show medians, box limits indicate the 25th and 75th percentiles, whiskers extend to minimum and maximum values, and data points are plotted as circles. LSP quantifications were performed with a custom pipeline using the open-source image analysis software CellProfiler. A second nucleus of Tumor 4 is depicted similarly to (**d–k**) above. **l** LSP depicting copies of *TERC* and CEP7; **m** LSP depicting copies of the chromosome 5 HPV16 insertion site; **n** LSP depicting copies of HPV16 DNA; **o** Color-coded merged image of the chromosome 5 insertion locus and HPV16 DNA. **p** Zoomed-in image of integrated HPV DNA marked by the number 1 in (**o**). **q** Bar graph depicting the number of LSP signals identified in the nucleus shown in (**l–o**). Box plots depicting the area (**r**) and intensity (**s**) of each LSP signal identified in the nucleus in (**l**–**o**). Center lines show medians, box limits indicate the 25th and 75th percentiles, whiskers extend to minimum and maximum values, and data points are plotted as circles.

The combination of long-range DNA sequencing combined with HC + SEQ analysis allowed recognition that the HPV DNA insertion in Tumor 4 existed at least predominantly as extra-chromosomal, HPV-human circular episome or as an integrated, tandemly repeated hetero-catemer (Fig. 2d). Sequence alignments permitted determination of the circularization point in the human genome with single base pair precision (Fig. 2d, Supplementary Figure 3). HPV DNA has previously been suggested to exist in such chimeric episomal form in some tumors^20,28,29^. Moreover, we detected additional structures derived from DNA in the vicinity of the HPV16 integration site in Tumor 4 that contained only human or HPV DNA sequences (Figs. 7 and 8). These unexpected structures were detected by combined application of HC + SEQ, long-range nanopore sequencing, and FISH analysis of the primary tumor.

While not necessarily integrated into human chromosomes per se, hetero-catemer episomes are derived from virus-human joining events comparable to chromosomal insertions of the viral DNA. Moreover, even long-range sequencing may not distinguish extrachromosomal episomes from chromosomally-integrated tandem repeats (Fig. 2d), and both forms may even coexist in tumors. How such extra-chromosomal DNAs are maintained and segregated into daughter tumor cells during mitosis are important questions. Likewise, the role of such episomes in etiology and/or maintenance of these tumors represent significant scientific inquiries. Clearly, the RNA-seq analysis showed the typical tumor pattern of E6*I and E7 viral transcription in Tumor 4 (Fig. 3), presumably off the episomal or tandemly repeated templates thus allowing the encoded viral proteins to contribute to tumorigenesis. The human sequences in the 38 kbp episome derived from the second intron of *C5orf67* (Fig. 2). Only a limited portion of those human sequences from the antisense strand of *C5orf67* fused to HPV16 were observed in the RNA-seq data (Fig. 4), and it is unknown whether they could play any role in tumorigenesis. Nonetheless, application of the combined sequencing approaches used here allows elucidation of precise structures of such HPV episomes/integrated tandem repeats and should be useful for understanding the roles of such elements in tumorigenesis.

In Tumors 2 and 4, with integrated subgenomic HPV fragments, the viral structural proteins encoded by the L1 and L2 genes were situated upstream of the viral promoters and transcription start sites in the URR-E6-E7 segment. In addition, each of the viral genomes was inserted in the transcribed portion of a human gene with the viral genomes in the opposite transcriptional orientation as the human gene (Fig. 2). Two out of the three human genes identified in this study have previously been linked to human cancers, which is a common phenomenon observed for genes located at or in close proximity to HPV DNA insertion sites in cervical cancer and HNSCC^58,59^. Tumor 5 contained an integration within the *LRP1B* gene, with few *LRP1B* transcripts detected. Low-density lipoprotein receptor-related protein 1B (*LRP1B*) is a putative tumor suppressor and a member of the LDL receptor family, a class of proteins that play a role in clearance of extracellular ligand-bound lipids. Silencing and down-regulation of *LRP1B* are found in various cancers, including renal cell carcinoma, and only low levels of *LRP1B* transcripts were observed here. Down-regulation of this gene also imparts resistance to chemotherapy in serous carcinomas, and integrations in it have been noted in HPV-positive cervical cancer samples previously^60–62^. Tumor 2 had integration within the NCK-associated protein 1 gene (*NAP1/NCKAP1*). *NCKAP1* is part of the WASF regulatory complex that includes *CYF1P1, ABI2, HSPC300/BRICK1* and *WASF1/SCAR* that is recruited to the cell membrane by cytokines and growth factors and impacts the actin cytoskeleton formation^63^. It is required for motility and adhesion of cells and has been associated with invasion and metastasis in a number of tumors including breast cancer and non-small cell lung cancer (NSCLC)^64,65^, but we only observed low levels of *NCKAP1* transcripts. The low levels are reminiscent of data from hepatocellular carcinoma patients suggested that *NCKAP1* may function as a tumor suppressor through regulation of the cell cycle^66^. The *C5orf67* gene in which HPV16 DNA was integrated in Tumor 4 encodes a long non-coding RNA (lncRNA) class of unknown function. Overall, the HPV DNA structures in these VSSCs were similar to those in other HPV-induced tumors.

Transcription of the HPV16 genomes involved typical viral early transcripts including considerable splicing using standard viral junctions, often including splicing to human sequences to generate 3′ portions of the transcripts. In tumor 5, most RNAs detected encompassed the E6, E7, and the 5′ end of the E1 ORF, along with some splicing into the human sequences downstream from the E1^E4 5′ss of at the beginning of the E1 ORF (Figs. 3 and 4). Tumors 2 and 4 showed a similar pattern plus extensive splicing from the E1 5′ss to the 3′ss just upstream of the E4 ORF (Fig. 3), suggesting that this ORF might be translated in these two tumors. The protein encoded by the E1^E4 fusion ORF has diverse functions including binding to the cytokeratin network and DEAD-box proteins^67^. As expected, given the structures of the integrated DNAs where E5 was not present downstream of the URR-E6-E7 segment or only partially present (Fig. 4), no or few E5 transcripts were detected in any of the tumors. Investigations of E5 have led to it being considered an oncoprotein that can contribute to the viral life cycle via growth factor signaling and immune avoidance^68–70^. However, at least based on this small sample of VSCCs with integrated HPV DNA, it seems unlikely that E5 has a substantial role in maintaining these tumors, although it might have a role early in the tumorigenic process before clones of cells with integrated HPV DNA and E5 gene disruptions emerge. The late region genes were situated upstream of the URR-E6-E7 segments in the integrated viral genomes in Tumors 4 and 5, and they were disrupted in Tumor 2. Neither late gene was transcribed in the three VSCCs with integrated HPV16 DNA, consistent with them having no role in maintaining these tumors, nor being possible targets of immune responses in them.

One notable aspect of HPV16 transcription in the vulvar VSCCs was the extensive splicing in the E6 ORF which yielded in-frame internal truncations within the E6 ORF encoding mainly the E6*I form of E6 (Fig. 3). E6* splicing occurs widely in hrHPVs mRNAs^71,72^. The E6*I spliced RNA form was by far the predominant E6-E7 segment transcript observed in all three tumors. Excision of the 182 nucleotide E6*I intron results in the mRNA encoding a frame-shifted E6 ORF (i.e., E6*I) that encodes a truncated E6 isoform with just two amino acid codons downstream of the splice junction followed by a stop codon. In addition to encoding the E6*I truncated form of E6, the E6* RNA splicing reduces the levels of full-length E6 ORF encoding mRNAs, and shortens the 5′ untranslated region for E7-encoding mRNAs^73^. E6* transcripts also predominate in cervical cancers and in high grade, cervical intraepithelial neoplasia 3 compared to low grade lesions^74,75^. Investigations of the potential role of E6* splicing in tumorigenesis have led to variable findings^75–80^ and warrant further investigation. In summary, the remarkable prevalence of E6*I splicing observed in these vulvar cancers suggests a substantial abundance of mRNA transcripts encoding E6*I- and E7, far greatly predominating those of full-length E6. This finding underscores the potential significance of the proteins encoded by the former transcripts and raises questions regarding the requisite levels of full-length E6 protein in these tumors.

Interestingly, despite the small number of samples analyzed here, the differentially expressed gene (DEG) analysis found similarities to results reported for HPV-induced tumors at other anatomical sites, particularly the elevated expression of the *STAG3*, *RNF212*, *SYCP2*, *ZFR2* genes, raising the possibility of common underlying mechanisms. Zhang et al. ^34^ conducted a DEG analysis of The Cancer Genome Atlas (TCGA) cohort, comparing HPV-positive and HPV-negative cervical cancers, and identified 166 genes with differential expression. Among them, eight were significantly associated with prognosis including *RNF212* and *ZFR2*. Notably, in both the cervical cancer cohort investigated in^34^ and our vulvar cohort, these two genes exhibited elevated expression in HPV-positive samples. Additionally, higher expression of these two genes was associated with improved survival in the TCGA cervical cohort^81^. *RNF212* is a RING finger protein that may function as a ubiquitin ligase^82^. *ZFR2* is a zinc finger protein that binds to single stranded and double stranded DNA^83^. This gene is also overexpressed in HPV-positive head and neck cancers where its expression is positively associated with outcome^35^. Two additional genes among the top DEGs identified in this study have previously been reported as upregulated in HPV-positive tumors in HNSCCs^36,37^. Remarkably, we observed a similar pattern of overexpression of these genes in our cohort of HPV-positive vulvar carcinoma. *STAG3* encodes for the cohesion subunit SA-3 protein and is a member of the cohesion complex that regulates chromosome segregation during mitosis and DNA repair. Altered expression of cohesin genes has been found to contribute to cancer by causing aneuploidy and genomic instability^84^. *SYCP2* encodes a protein that is a major component of the synaptonemal complex that binds DNA at scaffold attachment regions. It is hypothesized that aberrant expression of this gene in HPV-positive cancers contributes to genomic instability and oncogenesis^37^.

Relative enrichments in plasma cells in the primary HPV-positive tumor samples (Fig. 5a, b), and in total B-lymphocytes (Fig. 5d, e) were noted relative to HPV-negative samples. Tumors frequently harbor B cells and plasma cells; however, the antigen specificity of these B cells within the tumor microenvironment has remained largely unknown. Recently, ref. ^85^ investigated HPV-positive and HPV-negative HNSCC and made a significant discovery by identifying the presence of HPV-specific, antibody-secreting B cells within HPV-positive tumors. The consequences of B cells in the tumor microenvironment are unclear. Nonetheless, the recent findings such as those reported here and by ref. ^85^ emphasize that further research is needed to address whether these B-lymphocytes play a functional role in the immune response against the tumor or if their presence reflects a generalized, heightened immune response, or whether these B-lymphocytes can serve as potential biomarkers for patient prognosis.

Finally, the WGS and FISH analyses provide additional insights about these tumors. No substantive differences were detected between HPV-positive and HPV-negative vulvar cancers in the WGS analysis performed. All the tumors had at least one sequence mutation in the *TP53* gene. The FISH analysis of Tumor 4 showed unexpected complexity of HPV16 DNA and its insertion locus within single cells. It detected likely chromosome insertions of human-HPV hetero-catemeric, tandem repeats as well as likely episomal DNAs consisting of only human DNA from the HPV16 integration locus, plus evidence of HPV16-DNA-only episomes, both of which matched amplified structures predicted from the WGS analysis of Tumor 4. These results emphasize the unique power of FISH microscopy to detect single cell-to-cell variability directly in HPV tumors. In terms of function of the unexpected human-only and HPV-only episomes, it is plausible to speculate that these extra-chromosomal DNAs, perhaps in concert with chromosomally inserted tandem repeats, might form so-called “hubs”^86^ in tumor cells where enhancers and oncogene promoters (HPV16 URR-E6-E7) on separate DNA molecules interact to contribute to tumorigenesis. It is interesting to note that the segment of chromosome 5 DNA on the human plasmids in Fig. 7 contains an enhancer from the *C5orf67* gene.

## Methods

### Sample collection and nucleic acid preparation

Tumors from women diagnosed with invasive vulvar carcinoma were collected as part of an ongoing tissue collection protocol of the Department of Obstetrics & Gynecology and Women’s Health at the Albert Einstein College of Medicine (*n* = 4) or obtained from The Cooperative Human Tissue Network (CHTN) (*n* = 9). The inclusion criteria for this study comprised women who had a confirmed diagnosis of vulvar cancer. No samples were excluded from the analysis. The study included a total of 13 samples, with a mean age of 67.07 ± 13.8. The demographic distribution of the participants was as follows: six were White, three were Black, two were Hispanic, and two were of unknown ethnicity (Table 1). The experimental procedures were approved by the Internal Review Board of the Albert Einstein College of Medicine (IRB#:2018-9256 and #2007-433), with written informed consent obtained in accordance with the IRB approval. The human studies conducted in this research were performed in full compliance with all relevant ethical regulations, including adherence to the principles outlined in the Declaration of Helsinki. From all samples, 10 µm sections of the frozen tissue were cut for H&E confirmation of the presence of viable tumor tissue with squamous cell histology comprising 75% or more of the epithelial cells in the sections as reviewed by a board-certified gynecologic pathologist (B.H.). DNA and RNA were extracted from sections of the frozen tissue using the QIAamp DNA Mini Kit and RNeasy Mini Kit (Qiagen), respectively, and stored at −80 °C until use. DNA and RNA concentrations were quantified using the Qubit Fluorometric Quantification method (Thermo Fisher Scientific) and their integrity assessed using the Bioanalyzer capillary electrophoresis system (Agilent). Samples were blinded for further analysis when possible.

### HPV DNA hybridization capture, short read Illumina sequencing and data analysis

Targeted enrichment of HPV DNA was performed using custom hybridization capture probes homologous to the full‐length genomes of 143 different HPV types as we previously described^21^. Briefly, 1 µg of tumor genomic DNA was mechanically fragmented to 200 bp (Covaris, Woburn, MA), and Illumina adaptors were ligated at each end using the KAPA Hyper Prep kit per manufacturer instructions. Libraries were then hybridized to the custom HPV biotinylated oligonucleotide probes specific for each of the 143 HPV types for 72 h using the Roche target enrichment protocol (Roche Nimblegen SeqCap EZ System, Basel, Switzerland) following manufacturer instructions. After library purification, the DNA was sequenced on one Illumina HiSeq 2500 lane (Illumina, San Diego, CA) using the paired end, 2 × 150 bp sequencing mode. The sequencing reads were next adaptor‐cleaned, and those that passed QC were de‐duplicated, and paired-end reads were aligned to a custom human (GRCh38/hg38) plus HPV reference genome containing 143 alpha genus HPV types from the Papillomavirus Episteme^10^ using the STAR aligner^25^. Average sequencing coverage of the sample-typed HPV genome after de-duplication was calculated based on the mapped, on-target, 150 bp reads. Junction fragments were computationally identified using CTAT-Virus Integration Finder developed by our group that specifies a chimeric read analysis to define integration sites^24^. Tumors 1–9 were multiplexed and sequenced on the same Illumina HiSeq lane with a prior HPV70 positive cervical tumor at our institution serving as a positive control^21^. Reads aligning to the HPV70 were identified in some of these vulva tumors. Cross-contamination is a recognized possible phenomenon when utilizing capture and high throughput sequencing possibly due to a combination of factors that include barcode mismatch during PCR amplification of the multiplexed sample^87,88^, misidentification of clusters on the sequencing flow cell^89^, and index switching created during sequencing which can result in cross-contamination upwards of 10%^90^.

### Long sequencing reads using the Oxford Nanopore Technologies (ONT) MinION

Tumor-derived gDNA was purified and concentrated using the genomic DNA Clean and Concentrator-10 (gDCC-10) kit following manufacturer instructions (Zymo Research); the concentration was assessed by Qubit and the DNA quality was evaluated by Nanodrop (Thermo Fisher Scientific). The purified gDNA was then sheared by g-TUBE (Covaris, Woburn, MA) to 10 kb. Genomic libraries were prepared using the Oxford Nanopore 1D ligation library prep kit SQK-LSK109 following manufacturer instructions. The prepared tumor DNA libraries were loaded onto an R9.4 flow cell and sequenced using a MinION Mk1b device (ONT) using the standard 48-h scripts. Post-sequencing base calling and FASTQ extraction was performed using Guppy v2.0 (ONT), whereby raw voltage channel data is translated into canonical nucleotides. All libraries and quality filtered pass reads (*Q* ≥ 7) were used for the subsequent analysis. Library-specific adaptors were trimmed and possible internal adaptors split using Porechop^91^ with default parameters for adaptor identification (90% identity), end trimming (75% identity), and internal splitting (85% identity). Sequence reads were aligned to our custom combined reference genome using Ngmlr^92^, a structural variant caller that uses a structural variant aware k-mer search to approximate alignments followed by a banded Smith-Waterman final alignment, along with a convex gap cost model to account for higher sequencing error frequencies associated with long reads. SVs were called using Sniffles^92^ with parameter adjustment for expected low coverage.

### Global transcriptomic profiling of tumor RNA and differential gene expression

For global transcriptomic analysis, about 2 μg of total RNA was isolated from six 10 μm tumor sections and subjected to oligoT magnetic bead enrichment. Libraries were prepared from nine samples that had RIN > 6.0 (Agilent 1000) following the NEB standard protocol as follows: cDNA was synthesized using random hexamer primers and M‐MuLV RT (RNaseH‐) followed by second strand synthesis with DNA polymerase I and RNaseH. The double-stranded cDNA was purified using AMPure XP beads (Beckman Coulter), end tail repaired, adaptor ligated, size‐selected, and PCR amplified before Illumina sequencing. A total of 150 bp insert cDNA libraries were sequenced to a depth of 30 million reads on one Illumina HiSeq 2500 lane (Illumina, Inc., San Diego, CA) using the paired-end 150 bp mode. Raw image data were transformed to sequenced reads by CASAVA and stored in FASTQ format. Raw reads were then filtered to remove adaptors, reads containing *N* > 10% (*N* representing undetermined bases), and reads of Qscore (quality value) of >50% of bases ≤5. Of ~144 million reads, 97.8% passed filtering with >96% having Phred scores > 30. Gene model annotation files were downloaded from the genome website browser (NCBI/UCSC/Ensembl) for (GRCh38/hg38) and in custom format from Papillomavirus Episteme^10^. Indexes of the custom reference genome were built, and paired‐end clean reads were aligned to the reference using STAR v2.7.9a^25^. Differential gene expression in HPV-positive samples compared to HPV-negative samples was conducted using the DESeq2 package^33^. The model was controlled for recurrence versus primary disease and administration of neoadjuvant chemotherapy and radiation therapy. Genes that were differentially expressed in HPV-positive samples with a false discovery rate (FDR) threshold of *p* < 0.1 were utilized for enrichment and pathway analysis. Gene ontology (GO) categorization and an over-representation test were performed. The top five GO biological process terms showed enrichment of gene related to extracellular structure, matrix organization and smooth muscle processes. Similarly, the top five most significant GO molecular function terms included extracellular constituents, integrin binding, metallopeptidase activity, and MHC class II binding and antigen binding (Supplementary Fig. 5). Kyoto Encyclopedia of Genes and Genomes (KEGG) pathway gene-set enrichment analysis was performed using the molecular signature database KEGG gene sets^93^ comparing the HPV positive tumors and HPV negative tumors, but no gene enrichment was observed at a FDR < 25%, likely a result of small sample size.

#### HC + SEQ, long-range, WGS, and RNA sequence data availability

HC + SEQ, long-range, and RNA sequence reads are available (PRJNA994918) at the Sequence Read Archive (SRA) of the National Library of Medicine, National Institute of Health.

### Profiling tumour-infiltrating cell subtypes

The gene expression matrix of the RNAseq data from 9 vulvar samples was uploaded into CIBERSORTx^41^ and EPIC^42^ web servers to generate the gene expression profile (GEP) using the default settings on both platforms. For the CIBERSORTx platform, this GEP was used to perform deconvolution and immune cell composition profiling with LM22, a validated gene signature matrix that includes T cell types, naïve and memory B cells, plasma cells, NK cells and myeloid subsets as described. For the EPIC platform, the tumor infiltrating cells reference profile was used as described^42^.

### HPV detection by PCR

HPV detection by PCR was performed using the standard GP5+/GP6+ primers located within the L1 region of HPV genome (GP5 + 5′-TTTGTTACTGTGGTAGATACTAC-3’ and GP6 + 5′-GAAAAATAAACTGTAAATCATATTC-3′). Negative controls were no DNA template and DNA isolated from the HPV-negative cell lines. Positive controls were DNA from HPV DNA-positive cervical cancers. PCR products were resolved on agarose gels. The 3′ end of the GP5+ primer is remarkable for sequence conservation in 23 mucosotropic HPV types (HPV-6, -11, -13, -16, -18, -30, -31, -32, -33, -34, -35, -39, -40, -42, -45, -51, -52, -53, -56, -58, -61, -66), however, sensitivity of the detection of individual HPV types varies, with HPV16 having the highest detection sensitivity^22^. Confirmation of the presence of HPV6 and HPV16 in Tumor 10 was performed using primers located within the E6 and E7 regions of each type respectively. The HPV6 E6 primers were 5′-ATGCACTGACCACAGCAGAG-3′ and 5′-GCGGTTTGTGACACAGGTAG-3′ and the HPV6 E7 primers were 5′-ATGAGGTGGACGAAGTGGAC-3′ and 5′-CGCAGATGGGACACACTATG-3′. HPV16 E6 primers were 5′-TTGCTTTTCGGGATTTATGC-3′ and 5′-CAGGACACAGTGGCTTTTGA-3′, and HPV16 E7 primers were 5′- CAGCTCAGAGGAGGAGGATG-3′ and 5′-GCCCATTAACAGGTCTTCCA-3′. Confirmation of the presence of HPV53 and HPV62 in Tumor 13 was performed using primers located with the E7 region of each type. The HPV53 E7 primers were 5′- CAATGCCATGAGCAATTGAA-3′ and 5′- ACTGCACCAACGACTCACAC-3, and the HPV62 E7 primers were 5′- CTACAAGAGCGTCCCGATGT-3′ and 5′- TCCAGTGCATCCGTCAGTAG-3′. PCR reactions were performed using GoTaq Green (Promega) following manufacture instructions using 35 cycles of 95 °C for 30 s, 60 °C for 30 s and 72 °C for 30 s, and the products were resolved using agarose gels.

### Validation of HPV DNA integration junctions by PCR

To validate the integrated HPV DNA segment in samples Tumors 2, 4 and 5, PCR primers were designed to flank each side of the HPV genome—human genome junctions obtained from HC + MPS split reads. For Tumor 2, the Chr2:183,893,660-HPV16:5588 primers were 5′-AGTGGCTCACGCCTGTAATC-3′ and 5′-CTGGGACAGGAGGCAAGTAG-3′ producing a 166 bp amplicon. The Chr2:183,893,652-HPV16:3582 primers were 5′-CCCTGCCACACCACTAAGTT-3′ and 5′-CCCGAGTAGCTGGGATTACA-3′ producing a 140 bp amplicon. For Tumor 4 the Chr5:55837174- HPV16 E5:4162 primers were 5′CCTCTGCGTTTAGGTGTTTT-3′ and 5′-CTTCCAAAGTGCTGGGATTG-3′ producing a 212 bp amplicon. The Chr5:56541344- HPV16 E5:4178 primers were 5′-TTGCTGCAACAATCTTTGGT-3′ and 5′-TAAAGTTGGGTAGCCGATGC-3′ producing a 207 bp amplicon. For Tumor 5, the Chr2:141072580-HPV16:2313 primers were 5′-GATGATGGAGGTGATTGAAGC-3′ and 5′-GGCTGGTGAGAAAGTTGAGG-3′ producing a 246 bp amplicon. The Chr2:141791869-HPV16:1133 primers were 5′-CCCATTATTTCTGATCGCTGA-3′ and 5′-GTATTGCCATACCCGCTGTC-3′ producing a 211 bp amplicon. All PCR reactions were performed using GoTaq Green (Promega) following manufacturer instructions using 35 cycles of 95 °C for 30 s, 60 °C for 30 s and 72 °C for 30 s. The resultant PCR products were resolved using a 2% agarose gel.

### Whole genome sequencing

The five HPV-positive and the eight HPV-negative tumors were subjected to whole genome sequencing (WGS) analysis (Novogene). After quality controls, the filtered WGS reads were aligned to the human reference genome (hg38) using BWA (v0.7.17)^94^, with an 12× coverage (ranging from 10.3× for Tumor 8 to 15.5× for Tumor 5). The alignment data were used for detecting SNV and small insertions/deletions (InDels) using GATK4 (v4.3.0)^95^. SVs were called by DELLY^96^, and CNVs were called by Control-FREEC^97^. The detected variants were annotated by ANNOVAR^98^.

### Fluorescence in situ hybridization (FISH) on vulva Tumor#4 frozen tissue section

Fluorescence In Situ Hybridization (FISH) analysis was performed to visualize the integration of HPV in vulva Tumor 4, utilizing protocols described previously^99^. Tissue sections, ~5–7 µm in thickness, were obtained from vulva Tumor 4, preserved in Optimal Cutting Temperature compound, and were fixed in ice-cold methanol for 10 min. A 10-min incubation with 30 µl of pepsin solution (100 mg/ml in 0.01 N HCl) was used to facilitate the subsequent hybridization of custom DNA probes. To visualize genomic regions of interest we used Bacterial Artificial Chromosome (BAC) clones RP11-990E14 (hg38 -chr3:169,692,858-169,870,368 mapping to Telomerase RNA Component *TERC*), and RP11-662P23 (hg38 - chr5:56,420,151-56,608,998 mapping to the specific region of HPV16 integration on chromosome 5 in Tumor 4). In addition to human BAC clones we also used a plasmid containing the entire genomic sequence of HPV16 as reported in^100,101^. BAC DNA and HPV16 plasmid DNA was isolated and labeled by nick translation as we previously described^102,103^ using the following dUTPs from Dyomics (Jena, GE, USA): DY-495-dUTP (within the green spectrum to visualize RP11-662P23); DY-530-aadUTP (within the gold spectrum to visualize RP11-990E14); and DY-590-dUTP (within the red spectrum to visualize HPV16). All dyes were used at concentration of 1 mM diluted in water. To visualize copy number changes of chromosome 7 we used the commercial probe D7S522/CCP7 from Cytotest (Rockville, MD) labeled with CytoAqua and diluted 1:4 with the custom probes. Probes and slides were denatured simultaneously at 74 °C for 5 min and hybridized at 37 °C overnight using the ThermoBrite System (Abbott Molecular). Following hybridization, the slides were washed in 0.4× SSC/0.3% NP-40 at 74 °C for 2 min and then 4X SCC/0.1% Tween-20 at room temperature for 3 min. Slides were dehydrated with serial ethanol washing steps and mounted with 50 µl of VECTASHIELD antifade mounting media containing DAPI (Vector laboratories INC.) and covered with coverslip.

The same probes used on Tumor 4 were previously validated for specificity and sensitivity using interphase cells prepared form the UM-SCC47 cell line as described in^102^ that contains a known integration of HPV16 in the distal region of chromosome 3^15,20^.

### Image acquisition

FISH images were acquired with the BioView scanning system mounted on and Olympus BX63 microscope (BioView USA Inc., Billerica, MA) with a fine-focusing oil immersion lens (×60, NA 1.35 oil). Multiple focal planes (*n* = 11, 0.5 µm per plane) were acquired for each channel to ensure that signals on different focal planes were included. The resulting fluorescence emissions were collected using 415–475 nm (for DAPI), 558–587 nm (for spectrum gold), 465–497 nm (for spectrum aqua), 519–537 nm (for spectrum green), and 609–643 nm (for spectrum red) filters from Semrock Optical Filters (IDEX Health & Science, LLC; Rochester, NY).

### Data analysis of interphase cells

To identify and enumerate FISH locus-specific signals (LSP) in single cells, and to measure the size and intensity of each LSP, we constructed a customized pipeline using the open-source image analysis software CellProfiler (Version 4.2.6)^104^.

### Supplementary information

Supplementary Material

## Data Availability

Sequencing data generated in this study are available in the SRA of the National Library of Medicine, National Institutes of Health, under the accession number PRJNA994918. The dataset includes FASTQ files obtained from: targeted enrichment of HPV DNA obtained from custom hybridization capture probes homologous to the full-length genomes of 143 different HPV types; long-range sequencing performed using the Oxford Nanopore 1D ligation library prep kit SQK-LSK109 following manufacturer instructions; global transcriptomic pair-end sequencing; whole genome pair-end sequencing. These data can be accessed freely.

## Code availability

All data generated in this study have been processed using publicly accessible pipelines, as specified in their respective sections of the “Materials and Methods”.
