# Supplementary material for: Structure and transcription of integrated HPV DNA in vulvar carcinomas
Source: NPJ Genom Med. 2024 Jun 19;9:35. doi: 10.1038/s41525-024-00418-8 (PMC11187145; doi:10.1038/s41525-024-00418-8)
Supplement: Supplementary file 1 — Supplementary Material [file 41525_2024_418_MOESM1_ESM.pdf]

Supplementary data

Supplementary Figure 1: PCR screening assay to detect HPV DNA in 13 vulvar tumors.
Supplementary Figure 2: Sequences of the junctions between human and HPV16 DNA in Tumors 2, 4 and 5, and PCR validation of each.
Supplementary Figure 3: Integrated Genomics Viewer (IGV) display of long-range DNA sequence reads.
Supplementary Figure 4: PCR confirmation of HPV E6 and E7 DNA in Tumors 10 and 13 using primers specific for each HPV type.
Supplementary Figure 5: Principal component analysis (PCA) performed to assess transcriptional variability between HPV positive and HPV negative vulva tumors.
Supplementary Figure 6: Normalized read counts of the 10 most significant differentially expressed genes plotted by tumor HPV status.
Supplementary Figure 7: Gene Ontology (GO) enrichment analysis comparing HPV positive vulva tumors to HPV negative vulva tumors.
Supplementary Figure 8: Circos plots summarizing different types of genomic variability in HPV-positive vulvar cancers.
Supplementary Figure 9: Circos plots summarizing different types of genomic variability in HPV-negative vulvar cancers.

Supplementary Table 1: HC+SEQ sequencing metrics.
Supplementary Table 2: Differentially expressed genes between HPV+ and HPV- VVSC.
Supplementary Table 3: HPV DNA Integration Site Genome Coordinates with HC+NGS Sequence Junction Read Counts.

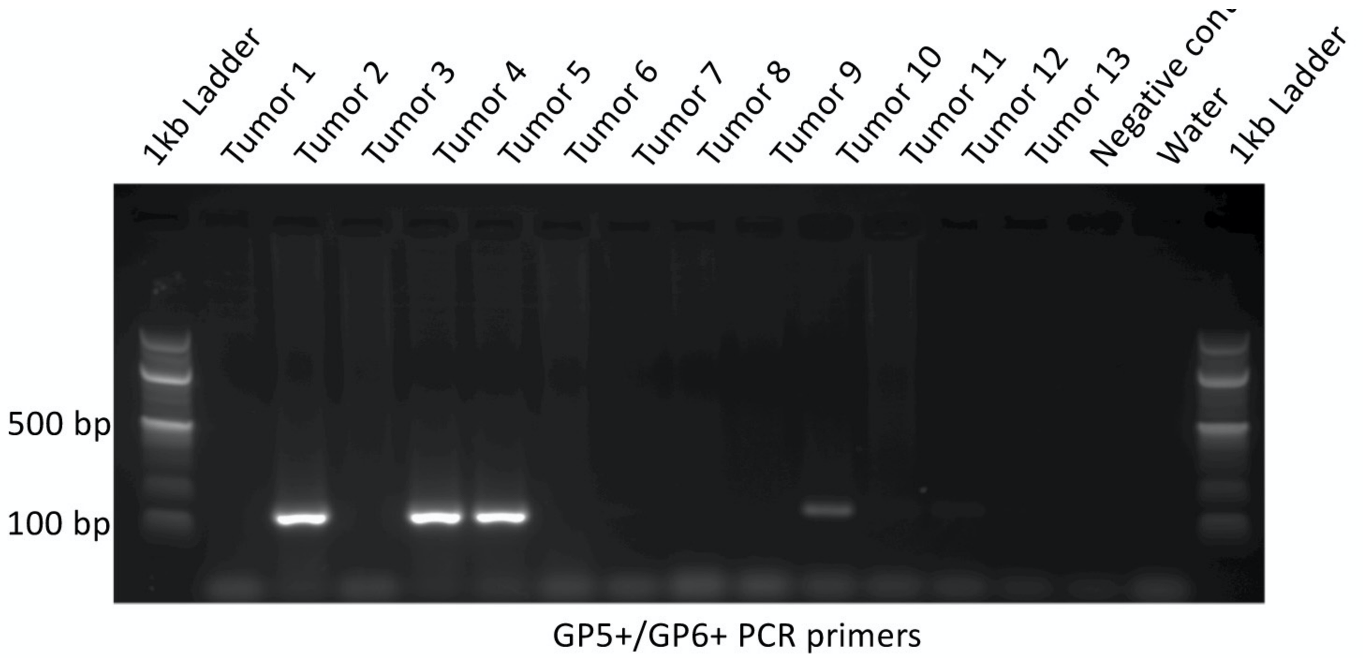

Supplementary Figure 1. **PCR screening assay to detect HPV DNA in 13 vulvar tumors.** The GP5+/GP6+ primer pair targeting a highly conserved region of L1 successfully identified the presence of HPV in Tumors 2, 4, 5, and 10. As a negative control, normal human DNA obtained from a healthy individual. Each row in the gel represents the PCR product obtained from each of the samples, with the first and last rows serving as reference for the 1kb ladder.
The agarose gel displayed shows samples that were processed simultaneously during the same experiment.

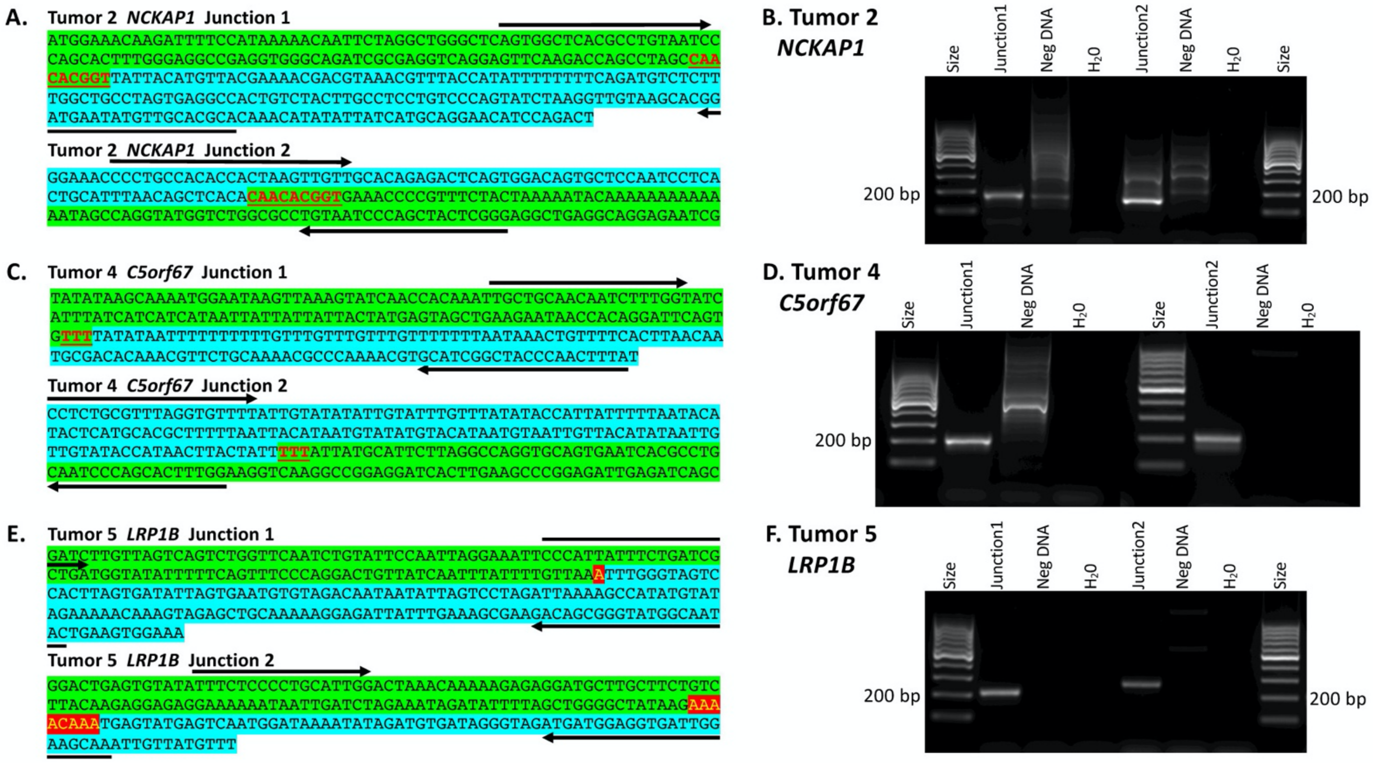

Supplementary Figure 2. Sequences of the junctions between human and HPV16 DNA in Tumors 2, 4 and 5, and PCR validation of each. Panels A, C, E) Sequences determined from aligned HC+SEQ reads are shown with human sequences highlighted in green and HPV sequences in blue for the indicated tumor and human gene. Both junctions are shown for each viral insert. Direct repeats immediately flanking each side of the inserted HPV16 DNA in Tumors 2 and 4 are shown in underlined, red text. The sequence at the HPV DNA insertion site in Tumor 4 is similar to the LINE1 consensus target sequence TTTAAAA [51], while that in Tumor 2 is not. Microhomology at each junction between the ends of the human and HPV DNAs in Tumor 5 is highlighted in red. Black arrows show the positions of the primers used for PCR across each junction. **Panels B, D, F**) Electrophoresis of PCR products to validate the indicated human-HPV16 DNA junctions. Positions of the 200 bp size marker are marked. NegDNA, negative control with normal human DNA. Each of the agarose gel depicted in panels B, D and F shows samples that were processed simultaneously during the same experiment.

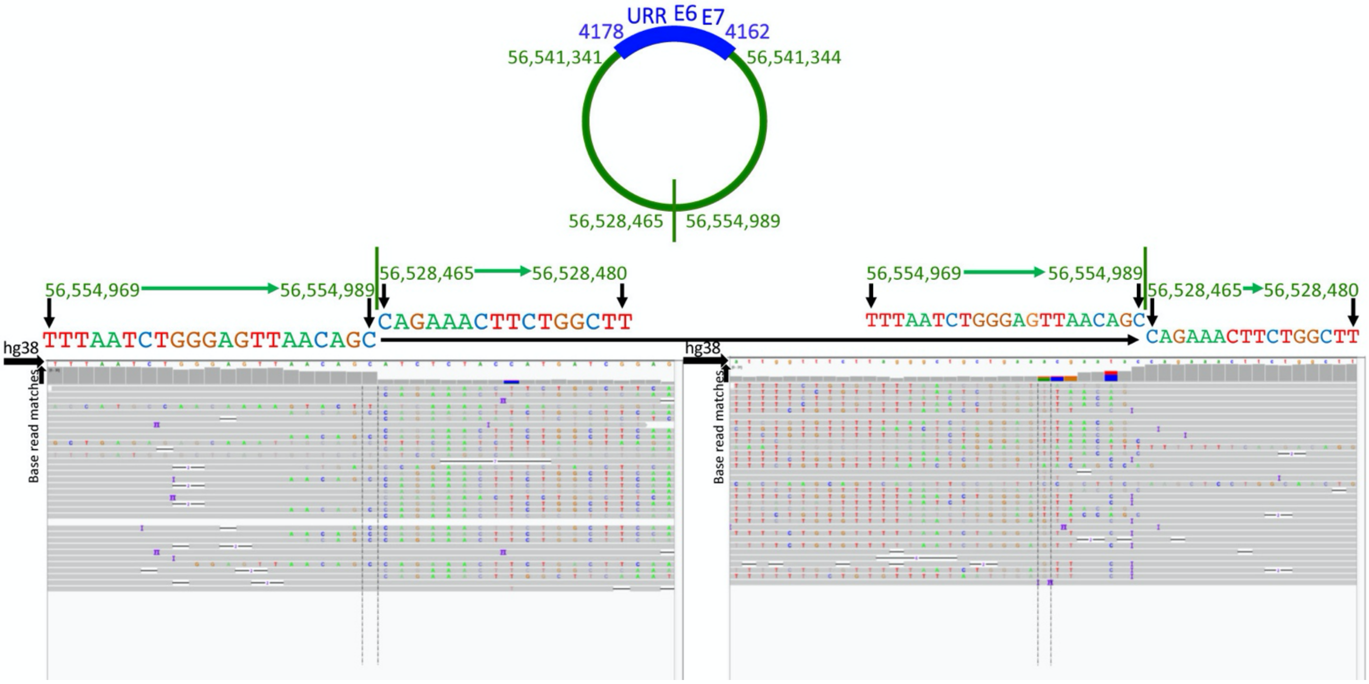

Supplementary Figure 3. **Integrated Genomics Viewer (IGV) display of long-range DNA sequence reads supporting the novel episome or concatemer junction in human genomic DNA.** Only the circular form from Figure 2D is displayed showing the positions of the novel junction below the circle. The figures at the bottom show individual nanopore reads aligned to the reference human genome (hg38), and a plot of the number of read counts. Sequences at the novel junction are enlarged above the IGV plots with reference genome positions indicated, and the position of the junction indicated by a vertical line.

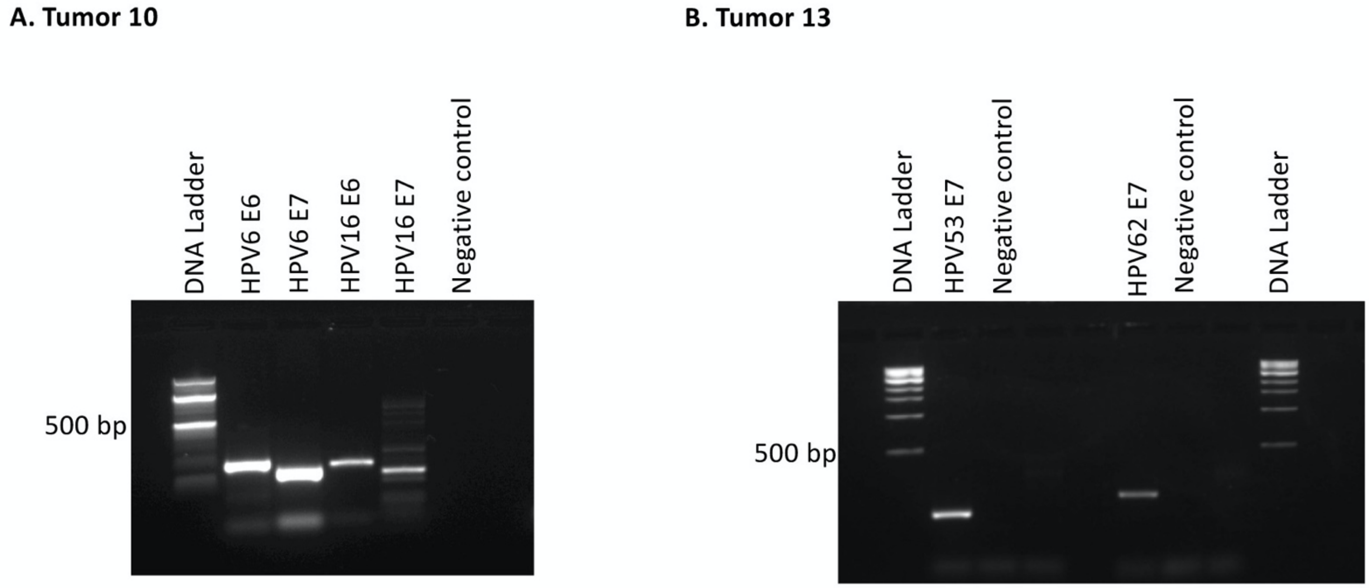

Supplementary Figure 4. PCR confirmation of HPV E6 and E7 DNA in Tumors 10 and 13 using primers specific for each HPV type. **A)** PCR confirming the presence of HPV6 and HPV16 E6 and E7 DNA in Tumor 10. **B)** PCR confirming the presence of HPV53 and HPV62 E7 DNA in Tumor 13. Negative controls were H_2O or whole blood DNA collected from a healthy donor. Each of the agarose gel displayed in panels A and B shows samples that were processed simultaneously during the same experiment.

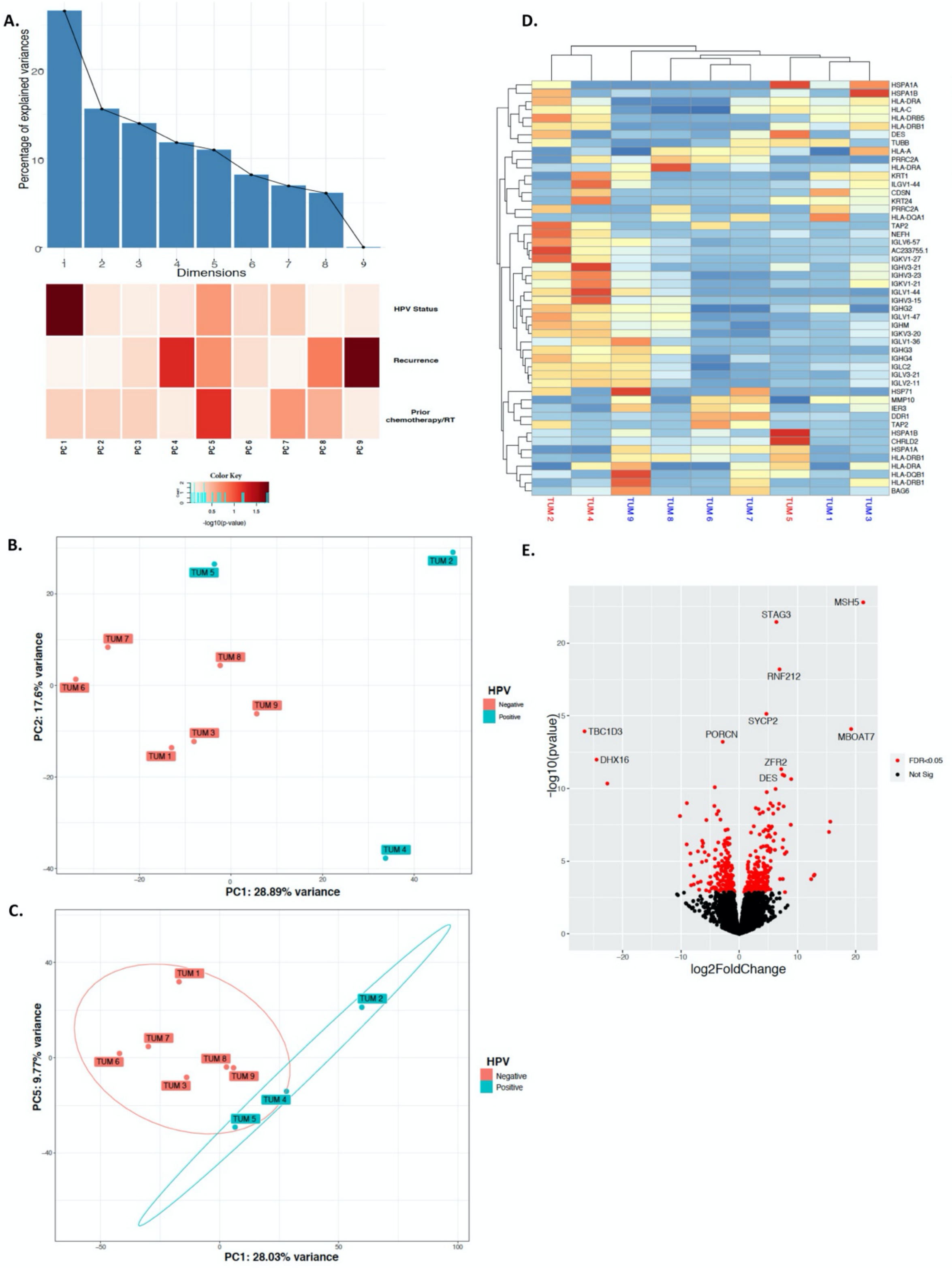

Supplementary Figure 5. **Principal component analysis (PCA) performed to assess transcriptional variability between HPV positive and HPV negative vulva tumors. A)** Scree plot of the amount of variance associated with each principal component (top) and heatmap (bottom) of the p-values of the association of the 9 major principal components (columns) and available clinical covariates (rows) including HPV positivity, whether tumor DNA was derived from a primary tumor or recurrence, and if the patient had received either chemotherapy and/or radiation therapy prior to tumor biopsy. Dark red signified a highly significant association between the PC and covariate. **B)** Plots of PC2 vs. PC1 and **C)** PC5 vs. PC1 for

HPV-positive (blue) and HPV-negative (red) tumors, showing that HPV infection status was significantly associated with PC1, and based on the heatmap in panel A, that PC5 was driven by the treatment covariate, specifically whether or not treatment with chemotherapy and/or radiation occurred prior to tumor biopsy. **D)** Unsupervised heatmap clustering of the regularized-logarithmic transformation values of the 50 genes with the highest variance across samples. Individual tumors are identified at the bottom with HPV-positive in red and HPV-negative in blue. Tumor 2 and Tumor 4 clustered together, however Tumor 5, also HPV positive, did not. **E)** Volcano plot of log fold change versus p-value after a differential gene expression analysis using DESeq2 comparing HPV positive to HPV negative tumors. Red points indicate genes that were differentially expressed with a FDR $p<0.05$. The 10 most significant genes are labeled.

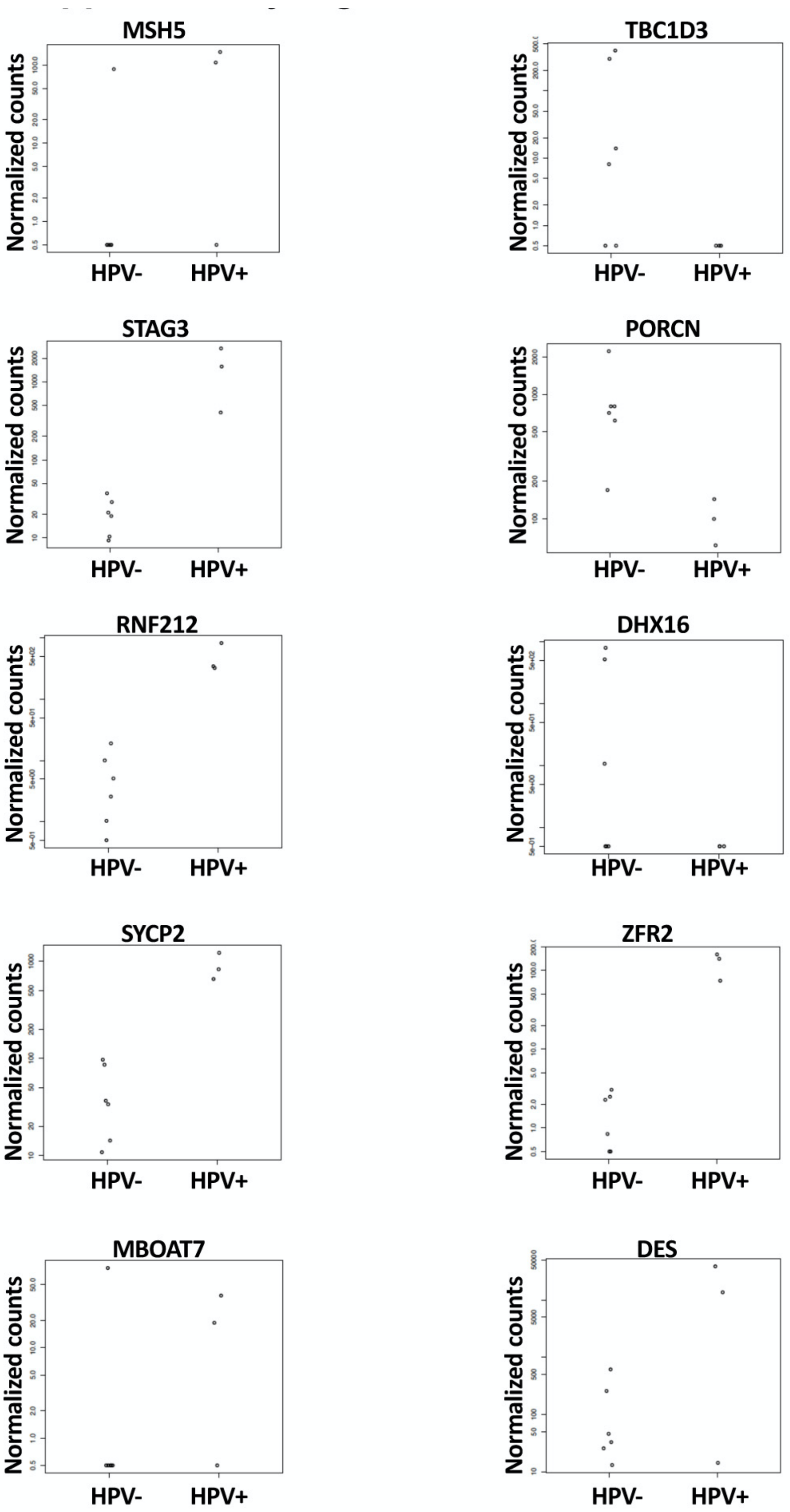

Supplementary Figure 6. Normalized read counts per million reads of the 10 most significant differentially expressed genes plotted by tumor HPV status.

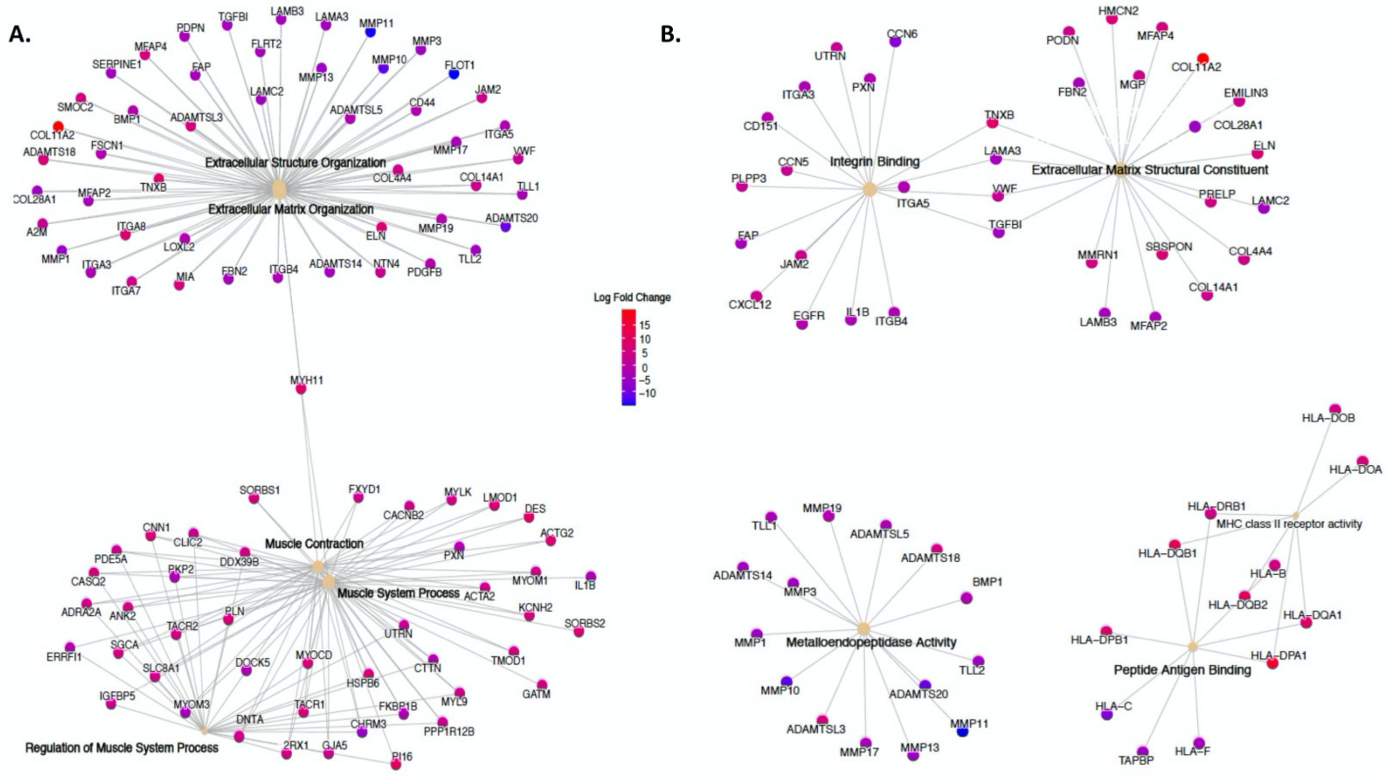

Supplementary Figure 7. **Gene Ontology (GO) enrichment analysis comparing HPV positive vulva tumors to HPV negative vulva tumors.** Differentially expressed genes are colored by log fold change with increased gene expression in HPV positive tumors colored in bright red and decreased gene expression in dark blue. **A)** The top 5 most significant biological process terms, which included extracellular matrix structure and organization and smooth muscle processes. **B)** The top 5 over-represented molecular function terms, which included integrin binding, extracellular matrix constituents, metalloendopeptidase activity, peptide antigen binding, and MHC Class II receptor activity.

Supplementary Figure 8A

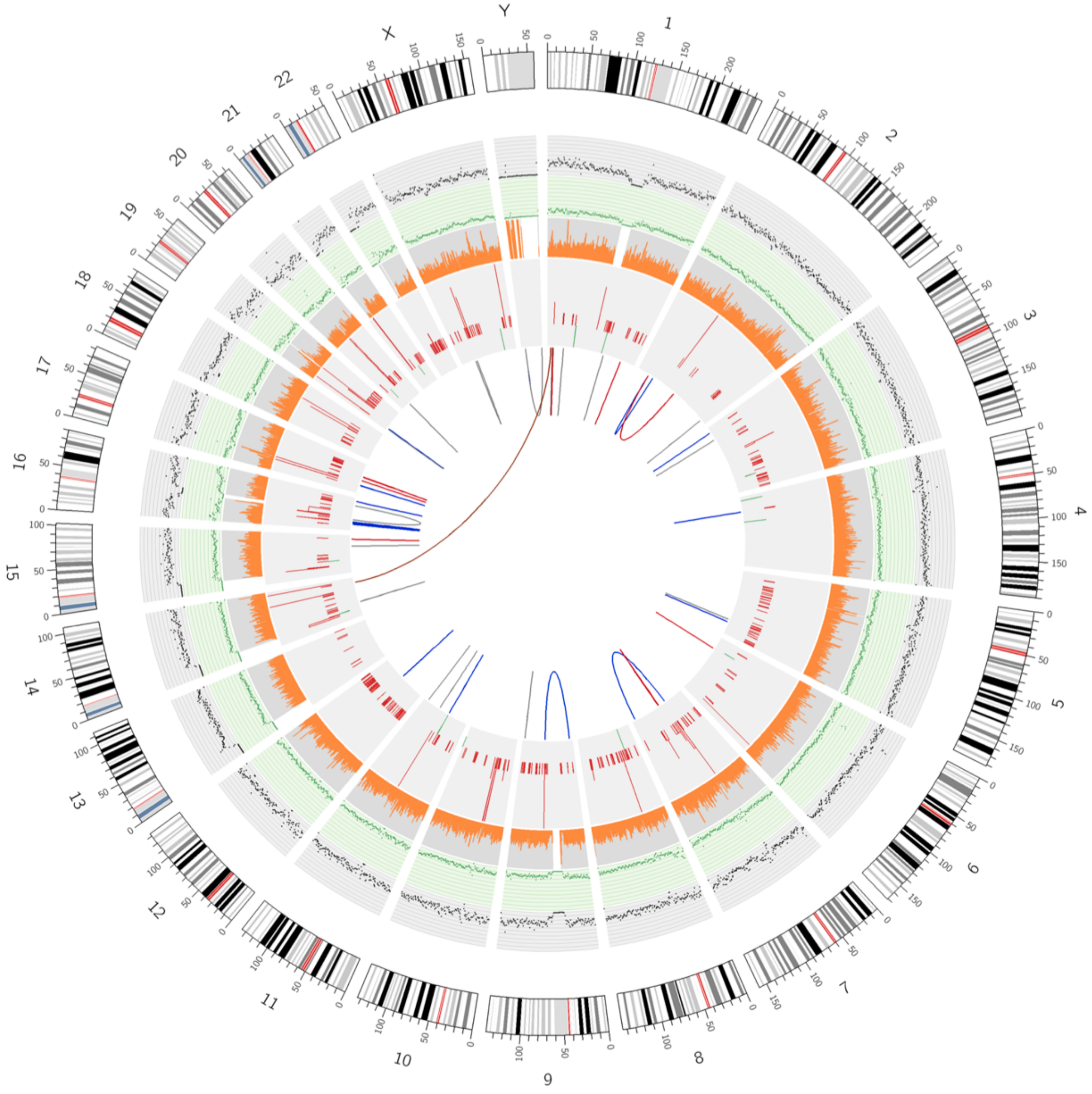

Tumor 2 (HPV+)

Supplementary Figure 8B

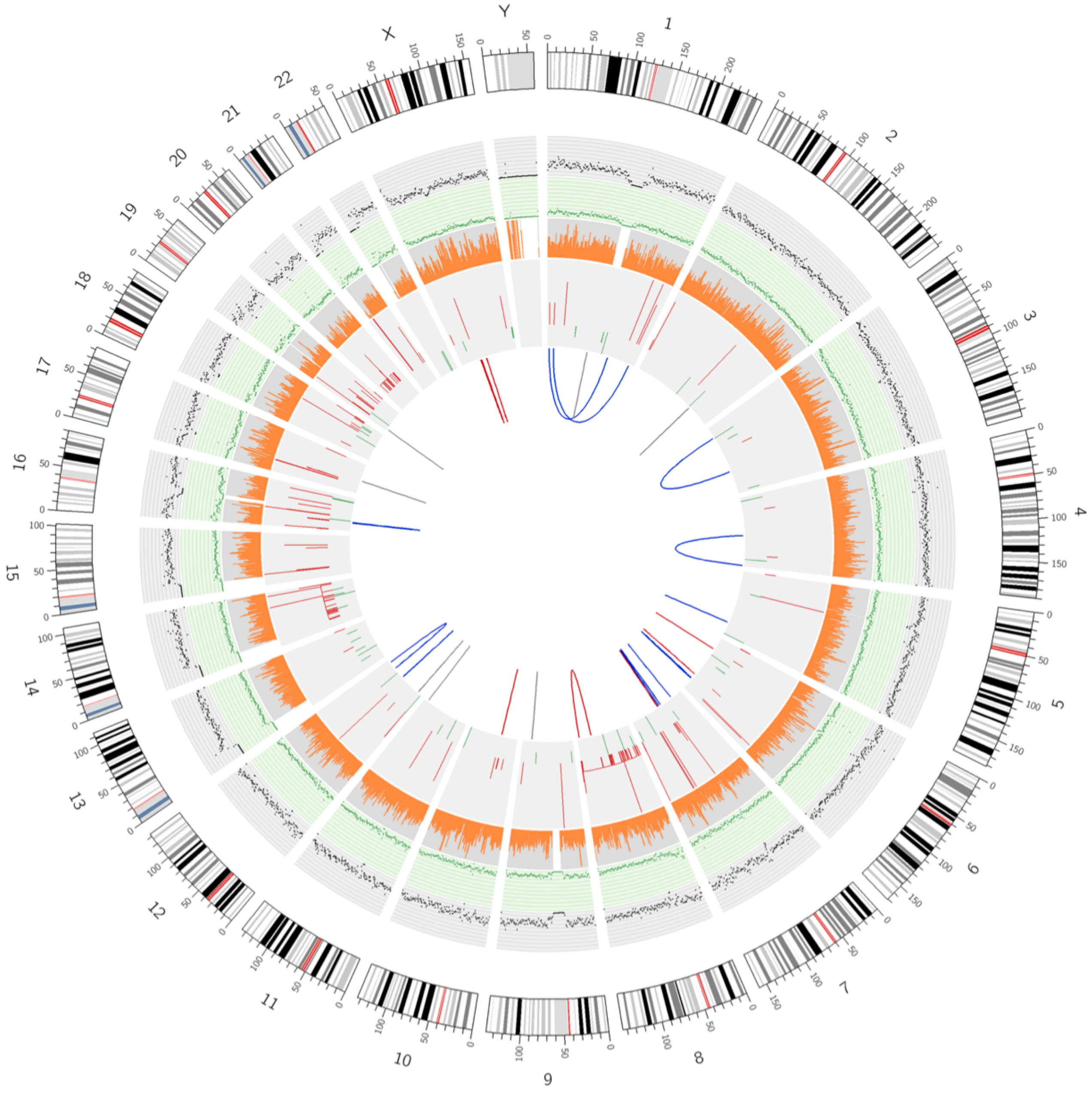

Tumor 4 (HPV+)

Supplementary Figure 8C

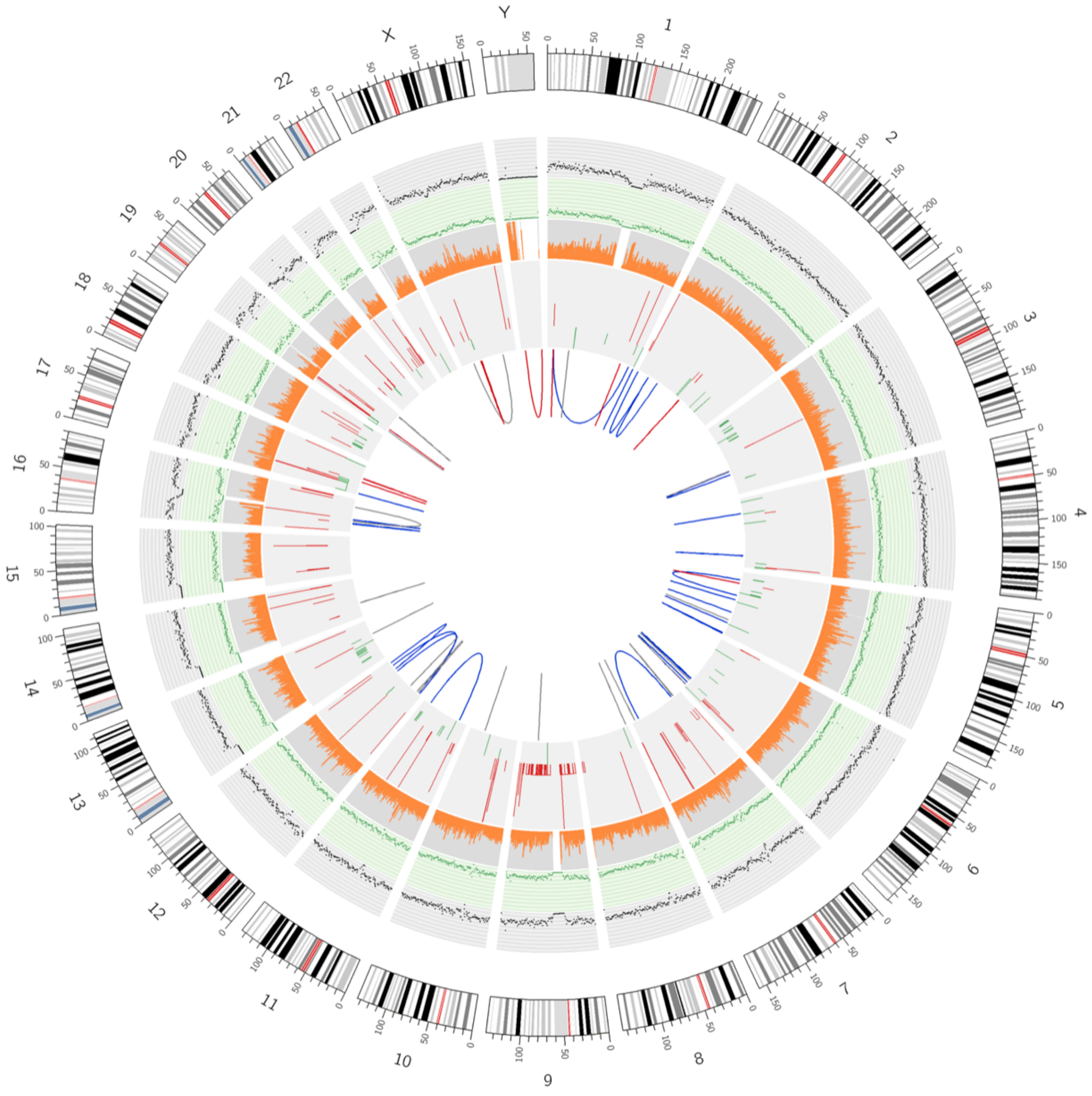

Tumor 5 (HPV+)

Supplementary Figure 8D

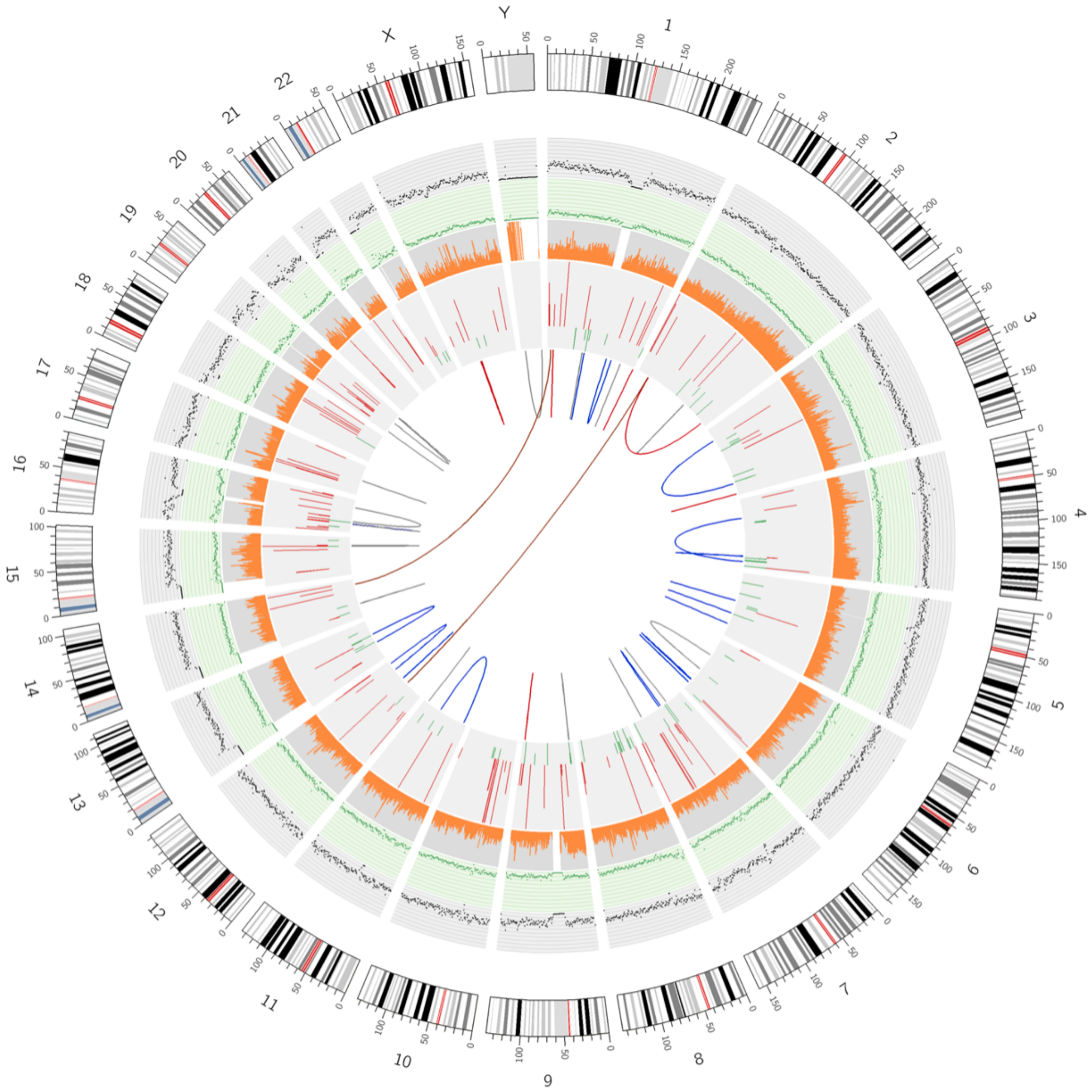

Tumor 10 (HPV+)

Supplementary Figure 8E

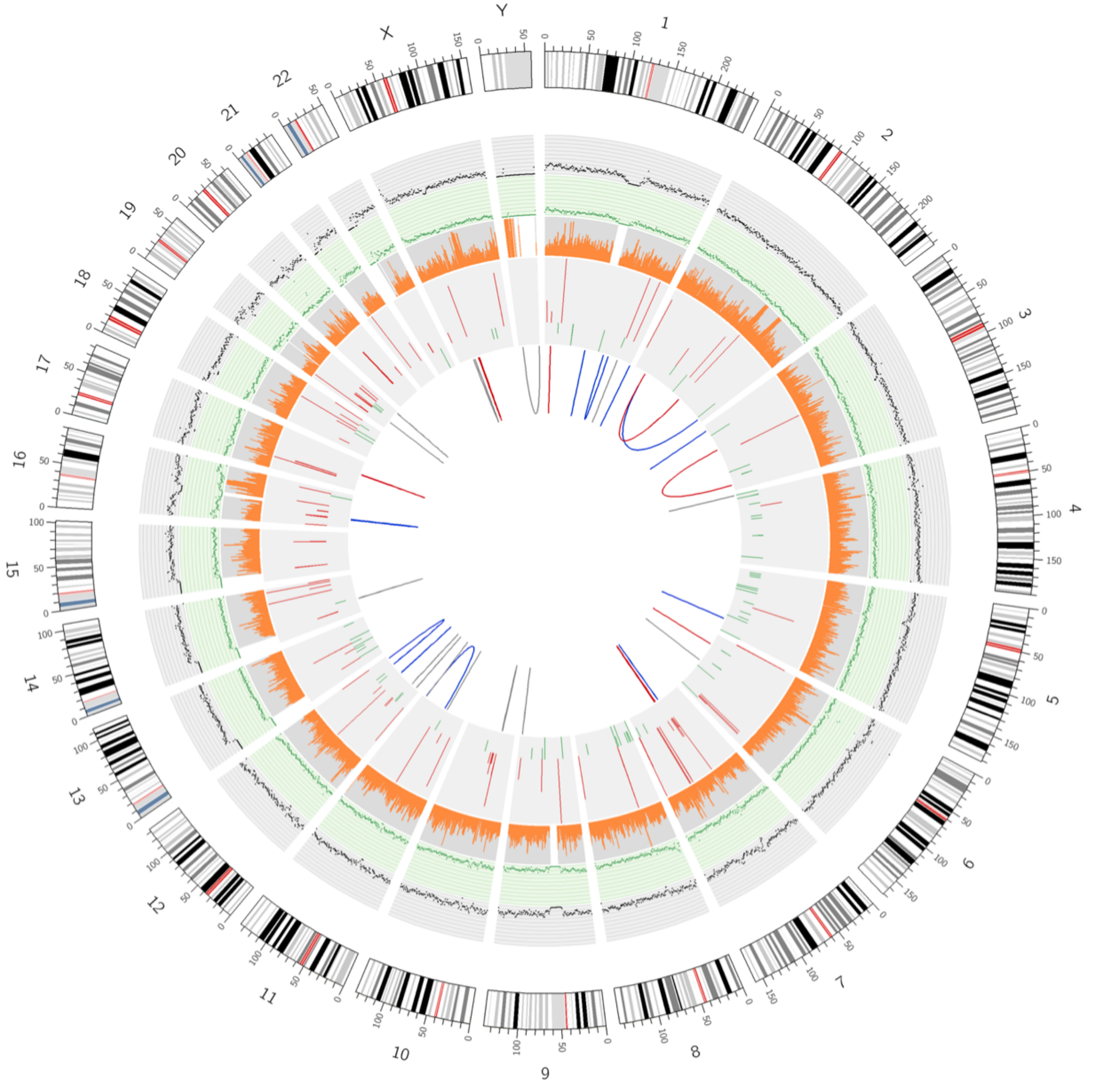

Tumor 13 (HPV+)

Supplementary Figure 8. Circos plots summarizing different types of genomic variability in HPV-positive vulvar cancers. Variation was determined versus human reference genome version hg38. The outer circles (the 1st ring) depict chromosomal positions. The 2nd rings represent InDel density in scatter style. A black dot is calculated as InDel number in a range of 1Mbp. The 3rd ring represents SNP density in scatter style. A green dot is calculated as SNP number in a range of 1Mbp. The 4th ring represents the proportion of homozygous SNP (orange) and heterozygous SNP (grey) in histogram style. A histogram is calculated from a 1Mbp region. The 5th ring represents the CNV inference. Red means gain, and green means loss. The most central ring represents the SV inference in exonic and splicing regions. BND (brown), INS (green), DEL (grey), DUP (red) and INV (blue).

Supplementary Figure 9A

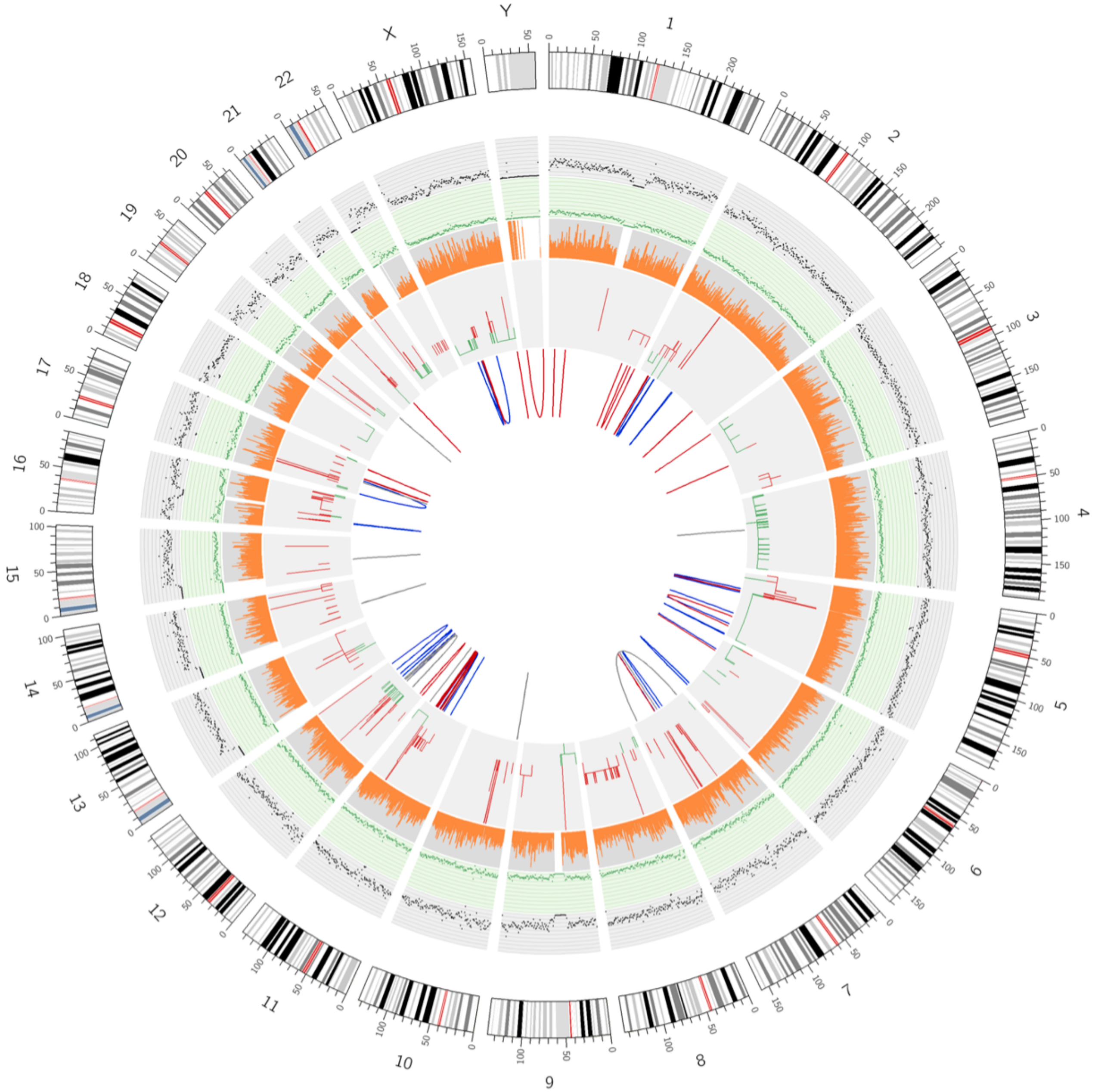

Tumor 1 (HPV-)

Supplementary Figure 9B

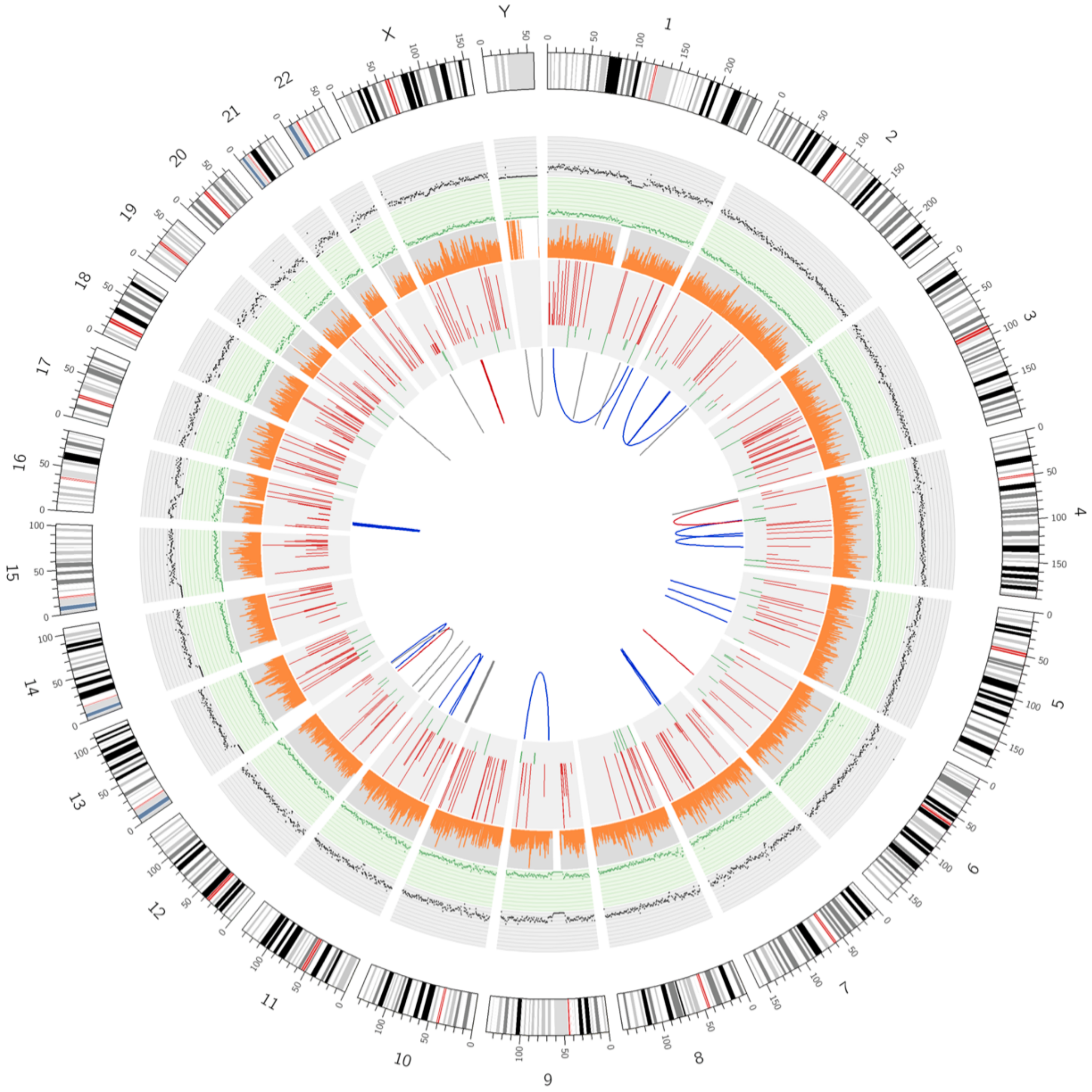

Tumor 3 (HPV-)

Supplementary Figure 9C

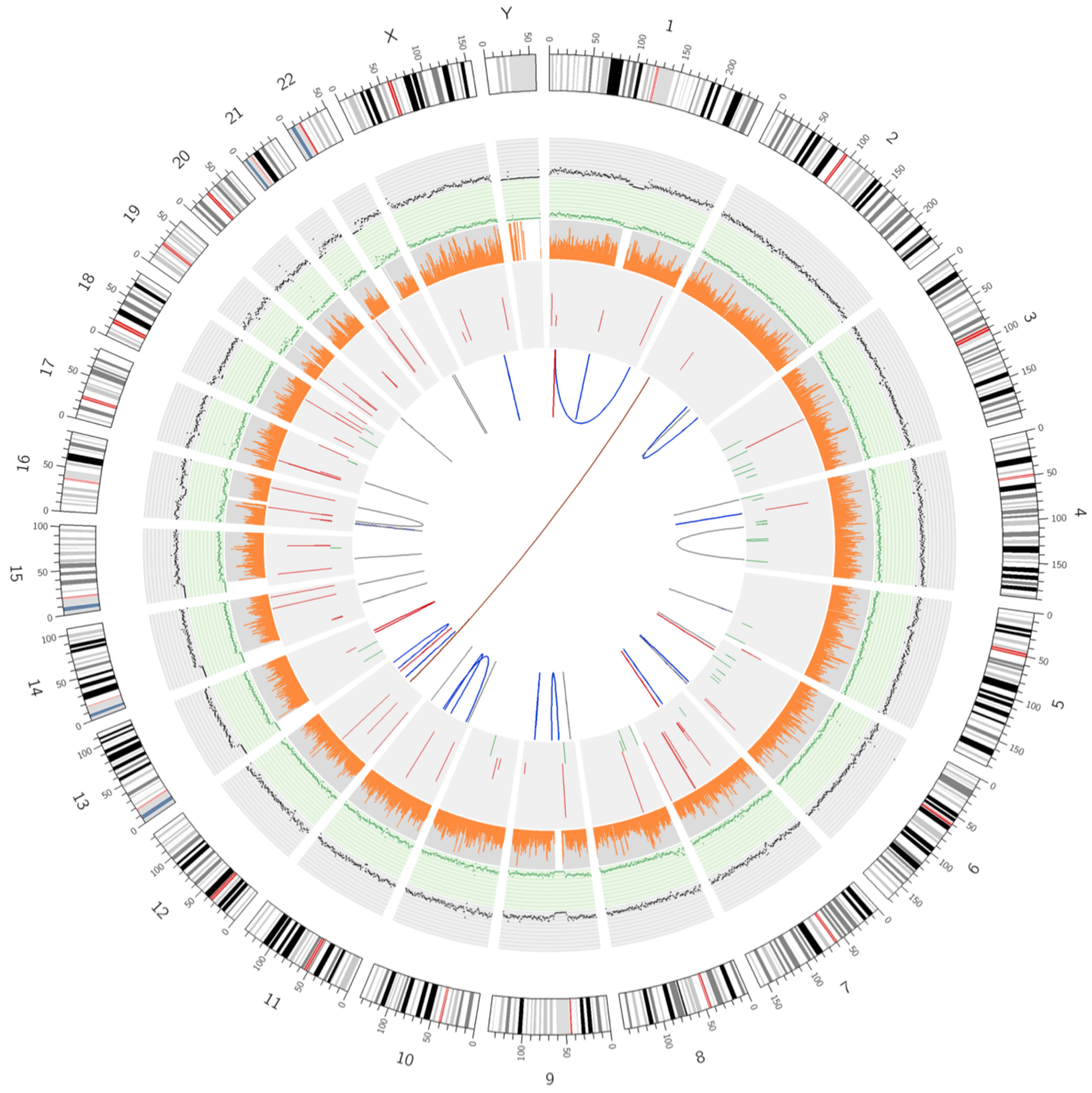

Tumor 6 (HPV-)

Supplementary Figure 9D

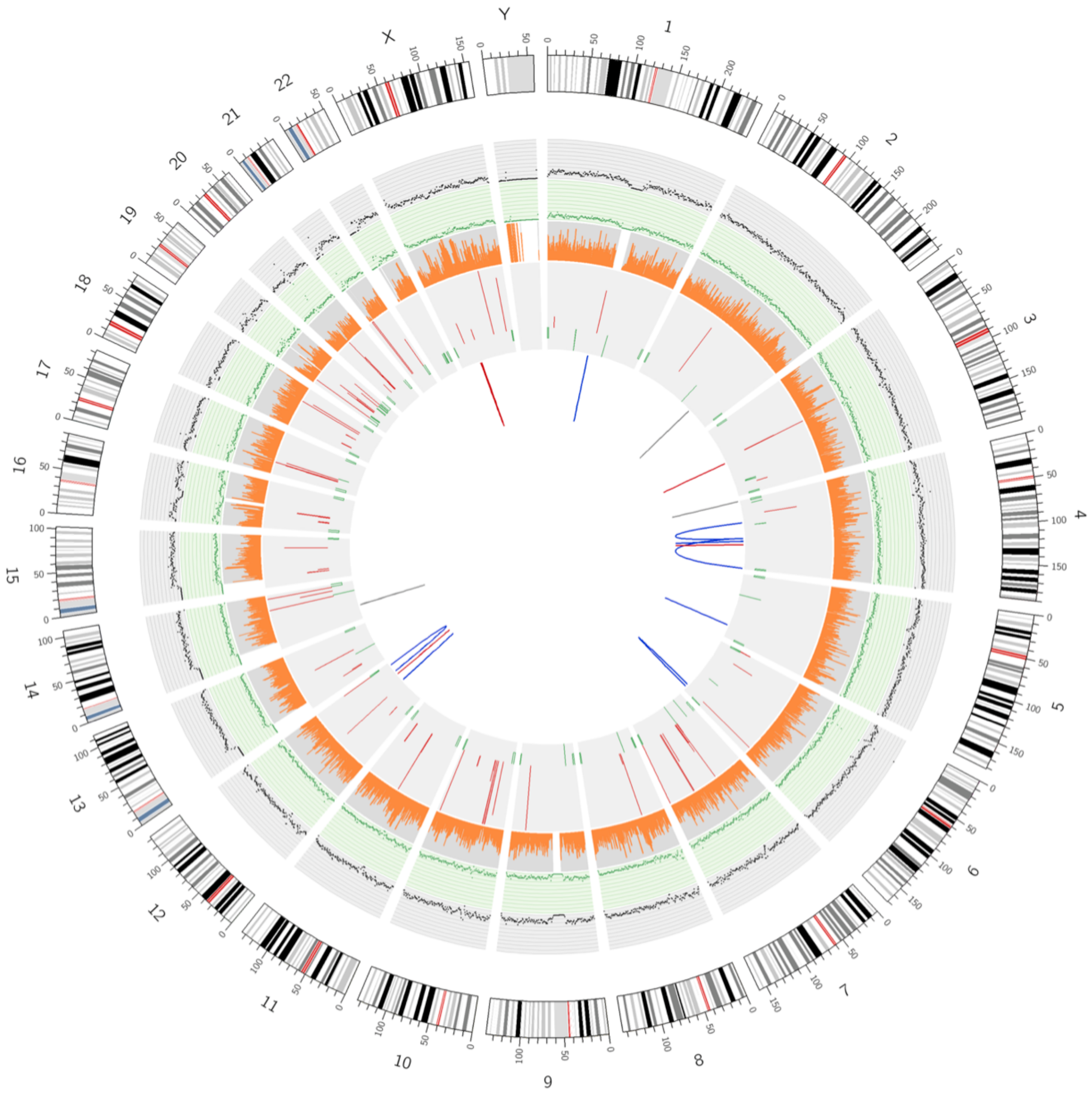

Tumor 7 (HPV-)

Supplementary Figure 9E

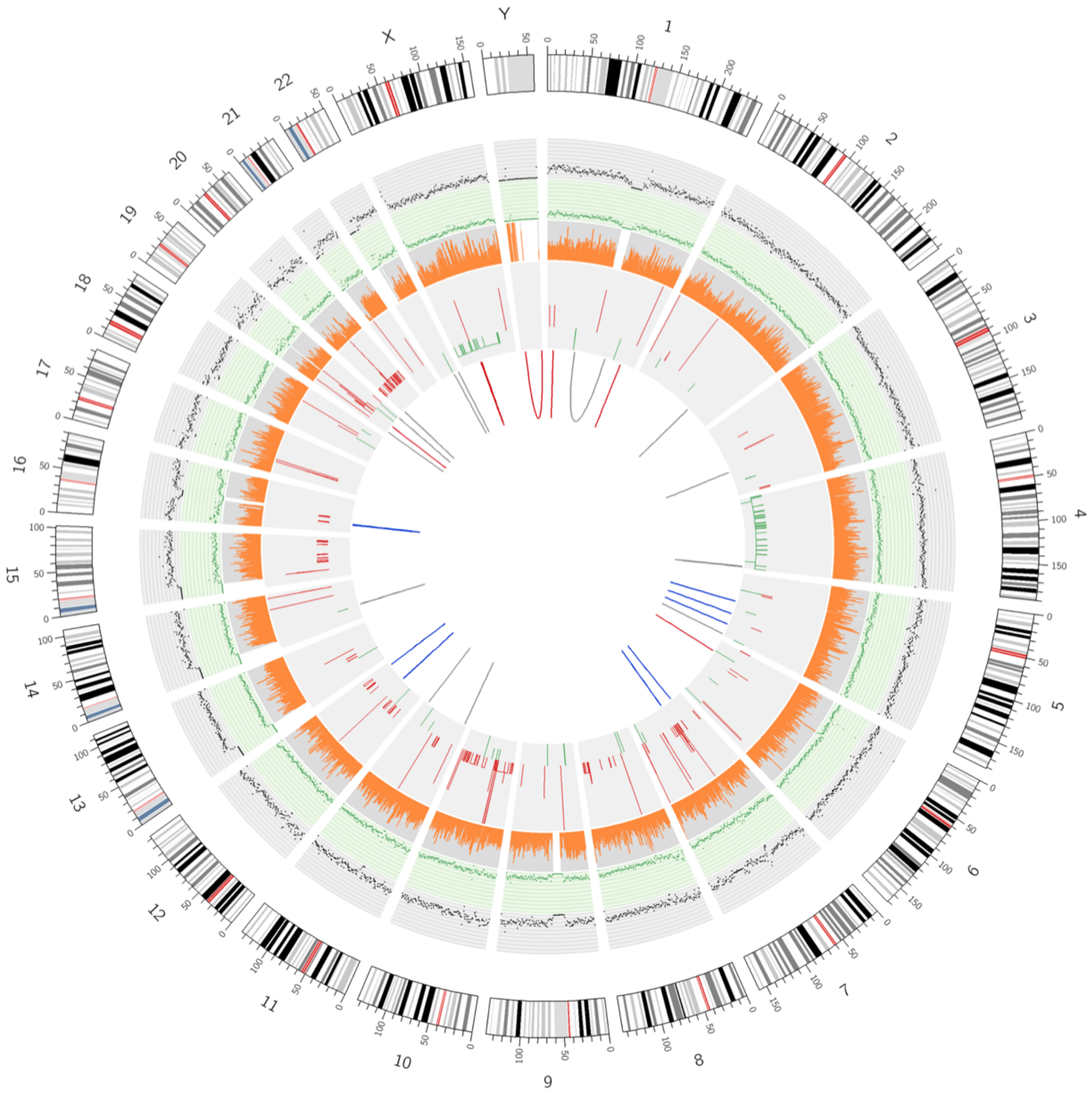

Tumor 8 (HPV-)

Supplementary Figure 9F

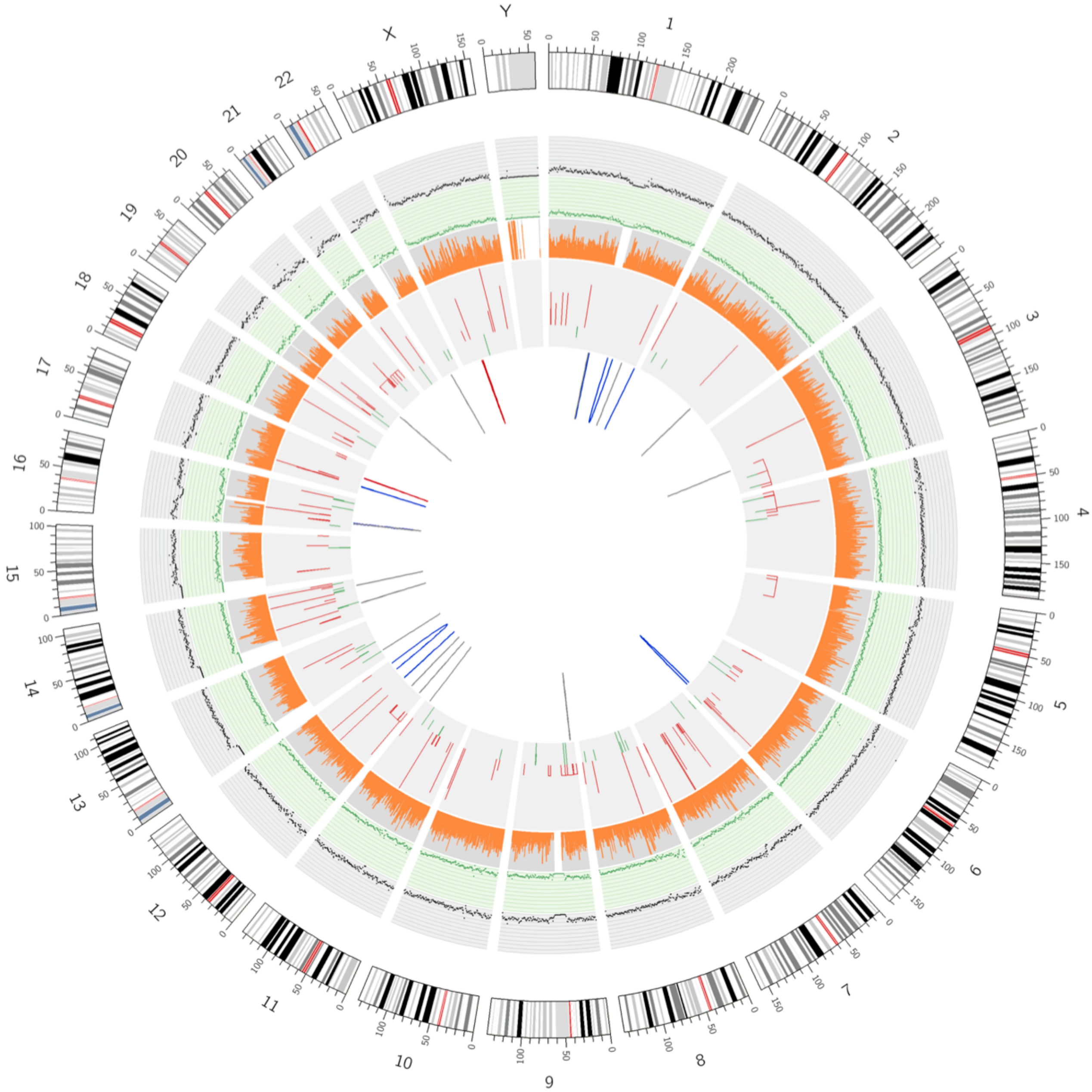

Tumor 9 (HPV-)

Supplementary Figure 9G

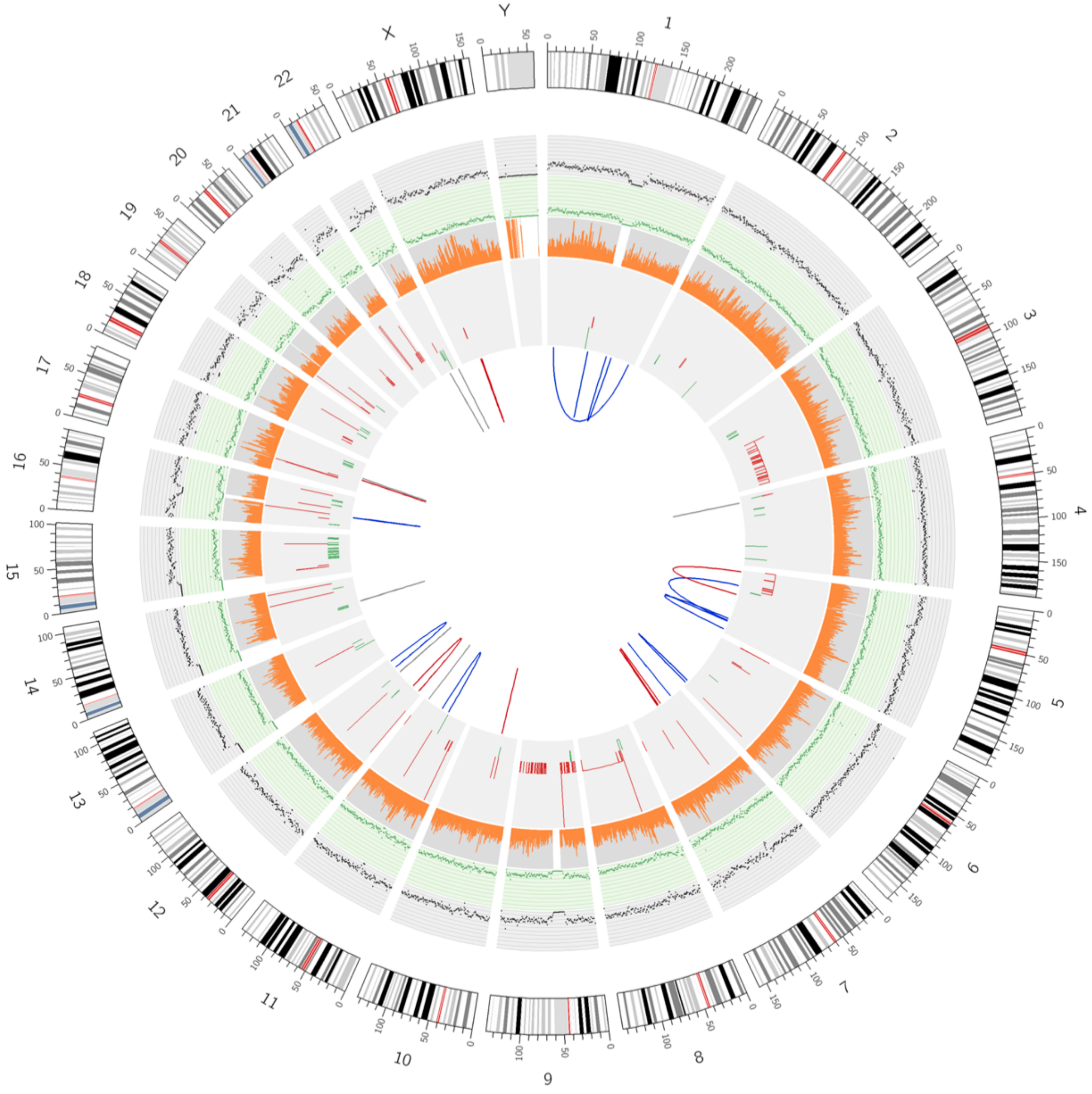

Tumor 11 (HPV-)

Supplementary Figure 9H

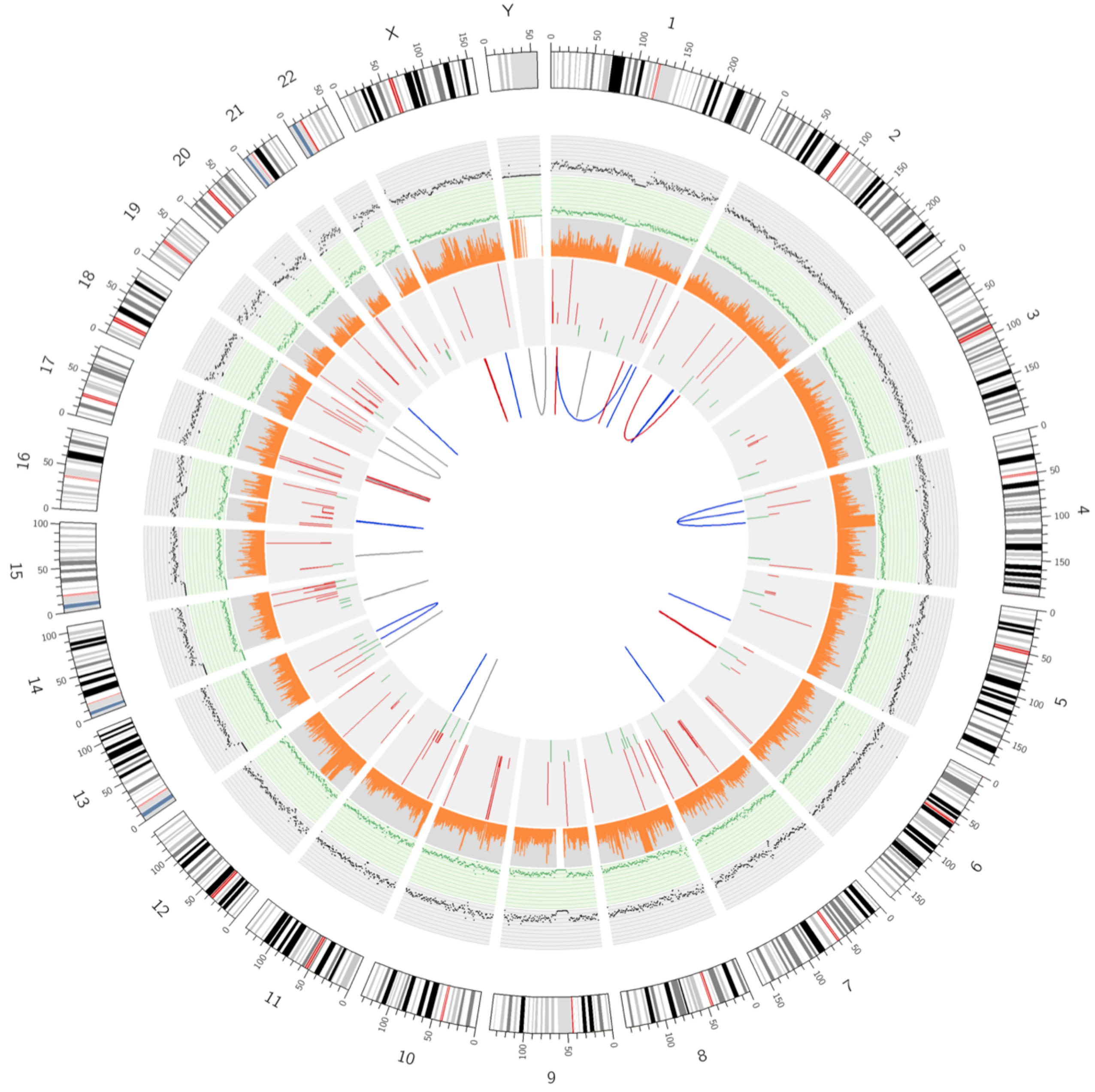

Tumor 12 (HPV-)

Supplementary Figure 9. Circos plots summarizing different types of genomic variability in HPV-negative vulvar cancers. Variation was determined versus human reference genome version hg38. The outer circles (the 1st ring) depict chromosomal positions. The 2nd rings represent InDel density in scatter style. A black dot is calculated as InDel number in a range of 1Mbp. The 3rd ring represents SNP density in scatter style. A green dot is calculated as SNP number in a range of 1Mbp. The 4th ring represents the proportion of homozygous SNP (orange) and heterozygous SNP (grey) in histogram style. A histogram is calculated from a 1Mbp region. The 5th ring represents the CNV inference. Red means gain, and green means loss. The most central ring represents the SV inference in exonic and splicing regions. BND (brown), INS (green), DEL (grey), DUP (red) and INV (blue).

Supplementary Table 1: HC+SEQ sequencing metrics

Hybridization capture and Illumina sequencing short-read metrics for individual tumor samples. Percent duplication ranged from 78.5-97.4%. The number of unique reads mapping to each sample and identified HPV type within a sample if positive is listed. Average HPV genome coverage by sample ranged from 139-8414X.

Sample	Total Paired Reads	Duplicate Paired Reads	Duplication (%)	Unique Paired Read Library	Paired reads mapping to typed HPV	Percent On Target (%)	Average HPV genome coverage
**TUM1**	43817090	42628736	97.3	1188354	N/A	N/A	N/A
**TUM2**	34430084	33364073	96.9	1066011	41107	4.05	1559X
**TUM3**	36935800	35711555	96.7	1224245	N/A	N/A	N/A
**TUM4**	41736950	40500501	97	1236449	114869	9.29	4359X
**TUM5**	46612544	45259680	97.1	1352864	85682	6.33	3251X
**TUM6**	51269454	49931701	97.4	1337753	N/A	N/A	N/A
**TUM7**	55052970	54046729	97.4	1459241	N/A	N/A	N/A
**TUM8**	43945609	42733271	97.2	1212338	N/A	N/A	N/A
**TUM9**	44705992	43482178	97.3	1223814	N/A	N/A	N/A
**TUM10**	27365615	21933347	80.2	5475266	Total: 371716	6.79	
					HPV6: 149980		5627X
					HPV16: 221736		8414X
**TUM11**	27916955	22266824	79.8	5700097	N/A	N/A	N/A
**TUM12**	19683451	15442671	78.5	4290332	N/A	N/A	N/A
**TUM13**	16817087	13238551	78.7	3617731	Total: 62211	1.72	
					HPV53:58453		2231X
					HPV62:3758		139X

N/A= not applicable

Supplementary Table 2: Differentially expressed genes between HPV+ and HPV- VVSC

Results of differential gene expression analysis in HPV positive (n=3) versus HPV negative (n=6) vulva tumors. Genes were retained if FDR p-value <0.1 given the sample size. Genes are identified by ENSEMBL ID, Gene Symbol and Entrez ID. Genes are ordered in decreasing fashion based on adjusted p-value.

Ensembl ID	baseMean	log2FoldChange	lfcSE	stat	pvalue	pvalue adjusted	Gene symbol	Entrez ID
**ENSG00000066923**	654.8179861	6.921994115	0.63713522	10.86424654	1.71E-27	2.78E-23	STAG3	10734
ENSG00000235222	36.73136994	21.18081345	2.206980586	9.597190651	8.22E-22	6.70E-18	MSH5	4439
ENSG00000178222	153.8939304	6.656416169	0.739493533	9.001317616	2.23E-19	1.21E-15	RNF212	285498
ENSG00000274611	80.45435117	-26.64053455	3.438411345	-7.74791957	9.34E-15	3.81E-11	TBC1D3	729873
ENSG00000102312	603.0406579	-2.713423836	0.358596133	-7.56679615	3.83E-14	1.25E-10	PORCN	64840
ENSG00000278519	14.86242334	19.24524354	2.560711408	7.515584723	5.67E-14	1.54E-10	MBOAT7	79143
ENSG00000213088	150.0956925	4.755505066	0.660990834	7.194509851	6.27E-13	1.46E-09	ACKR1	2532
ENSG00000206486	134.6408892	-24.27712761	3.436393185	-7.064711836	1.61E-12	3.28E-09	DHX16	8449
ENSG00000123080	232.9076367	2.789335067	0.404034797	6.90370009	5.07E-12	9.18E-09	CDKN2C	1031
ENSG00000133392	6696.375035	6.595923617	0.975282983	6.763086951	1.35E-11	2.20E-08	MYH11	4629
ENSG00000100285	340.7881007	7.788721348	1.166571087	6.676593853	2.45E-11	3.63E-08	NEFH	4744
ENSG00000235396	46.83113548	-22.89009023	3.436670599	-6.660542396	2.73E-11	3.71E-08	NOTCH4	4855
ENSG00000164400	201.630471	-4.149534379	0.627966496	-6.607891348	3.90E-11	4.89E-08	CSF2	1437
ENSG00000105278	29.59760307	6.502456279	1.014199325	6.411418466	1.44E-10	1.68E-07	ZFR2	23217
ENSG00000175084	6362.695793	7.52308566	1.190408953	6.319748889	2.62E-10	2.85E-07	DES	1674
ENSG00000139289	4263.25855	-2.448036184	0.397208874	-6.163095397	7.13E-10	7.27E-07	PHLDA1	22822
ENSG00000197172	104.6136588	-8.969997192	1.470502993	-6.099951674	1.06E-09	1.02E-06	MAGEA6	4105
ENSG00000004776	265.4338081	5.111305841	0.843362445	6.060627752	1.36E-09	1.21E-06	HSPB6	126393
ENSG00000122641	2839.445853	-4.210641455	0.69546989	-6.054383537	1.41E-09	1.21E-06	INHBA	3624
ENSG00000132470	12601.41694	-1.703363485	0.282149558	-6.037094289	1.57E-09	1.28E-06	ITGB4	3691
ENSG00000169116	245.3720527	3.313647258	0.554597095	5.974873091	2.30E-09	1.79E-06	PARM1	25849
ENSG00000148798	58.90703791	7.599364733	1.277681737	5.947775972	2.72E-09	2.01E-06	INA	9118
ENSG00000196074	254.4644857	3.948288236	0.667562103	5.91448828	3.33E-09	2.36E-06	SYCP2	10388
ENSG00000282399	337.4501777	6.75740633	1.145143403	5.900925866	3.61E-09	2.46E-06	NA	NA
**ENSG00000149243**	106.0809406	3.502199639	0.595539906	5.880713627	4.09E-09	2.66E-06	KLHL35	283212

ENSG00000058085	17322.7843	-3.49940035	0.596109244	-5.870401082	4.35E-09	2.73E-06	LAMC2	3918
ENSG00000156414	209.0485202	5.920207944	1.012148176	5.849151423	4.94E-09	2.98E-06	TDRD9	122402
ENSG00000100196	274.1089093	-1.983120443	0.339517113	-5.841002902	5.19E-09	3.02E-06	KDELR3	11015
ENSG00000095777	48.82227709	7.631553434	1.331992717	5.729425799	1.01E-08	5.67E-06	MYO3A	53904
ENSG00000226165	33.53761479	7.960493531	1.392582962	5.716351374	1.09E-08	5.86E-06	HLA-DQB2	3120
ENSG00000112562	212.9755907	4.056354279	0.710096051	5.71240225	1.11E-08	5.86E-06	SMOC2	64094
ENSG00000049540	707.5582786	5.469371915	0.96146631	5.688573651	1.28E-08	6.53E-06	ELN	2006
ENSG00000185247	152.1873626	-10.23222438	1.8032819	-5.674223413	1.39E-08	6.88E-06	MAGEA11	4110
ENSG00000106366	5317.391264	-3.199221574	0.566034511	-5.651990318	1.59E-08	7.61E-06	SERPINE1	5054
ENSG00000164825	116.6900977	-5.600606099	0.992360934	-5.643718838	1.66E-08	7.75E-06	DEFB1	1672
ENSG00000047936	77.66247615	-5.828395203	1.039345424	-5.607755677	2.05E-08	9.28E-06	ROS1	6098
ENSG00000109472	318.7217448	2.014617849	0.360776516	5.584115812	2.35E-08	1.04E-05	CPE	1363
ENSG00000109321	856.2749329	-2.048399726	0.36850986	-5.558602225	2.72E-08	1.17E-05	AREG	374
ENSG00000173641	253.003848	5.514750988	0.994528535	5.545090761	2.94E-08	1.23E-05	HSPB7	27129
ENSG00000100626	50.84609244	5.037027891	0.912026823	5.522894463	3.33E-08	1.36E-05	GALNT16	57452
ENSG00000227801	8.777282553	15.61305309	2.854815446	5.469023614	4.53E-08	1.80E-05	COL11A2	1302
ENSG00000231939	63.36953281	8.914247364	1.635621504	5.450067356	5.04E-08	1.95E-05	HLA-DQB1	3119
ENSG00000077943	416.4239603	5.794064694	1.068091473	5.42468959	5.81E-08	2.20E-05	ITGA8	8516
ENSG00000005884	9399.46208	-1.841073925	0.341866319	-5.385362126	7.23E-08	2.68E-05	ITGA3	3675
ENSG00000130176	1835.430048	5.071833456	0.943332044	5.376509245	7.59E-08	2.75E-05	CNN1	1264
ENSG00000163815	120.1933943	4.224891549	0.787201023	5.366979239	8.01E-08	2.84E-05	CLEC3B	7123
ENSG00000230708	400.3859767	6.684957195	1.254677443	5.328028518	9.93E-08	3.44E-05	HLA-DPB1	3115
ENSG00000118156	47.98328499	5.614251519	1.056628019	5.313366121	1.08E-07	3.66E-05	ZNF541	84215
ENSG00000167077	303.6154519	4.150134278	0.782447063	5.304044805	1.13E-07	3.77E-05	MEI1	150365
ENSG00000006118	2226.388398	-2.177580822	0.413777831	-5.26268122	1.42E-07	4.63E-05	TMEM132A	54972
ENSG00000275993	2133.750399	-1.64088464	0.312392163	-5.252643424	1.50E-07	4.79E-05	SIK1B	102724428
ENSG00000204304	173.9933544	5.111621067	0.979845972	5.216759789	1.82E-07	5.68E-05	PBX2	5089
ENSG00000163431	833.5343468	4.861762893	0.932400388	5.214243746	1.85E-07	5.68E-05	LMOD1	25802
ENSG00000236698	7.527733909	15.30137	2.944593073	5.196429395	2.03E-07	6.13E-05	NA	NA
**ENSG00000161609**	27.22119294	8.292778973	1.596840967	5.193240371	2.07E-07	6.13E-05	KASH5	147872

ENSG00000169282	61.13261105	7.794214613	1.509936484	5.161948663	2.44E-07	7.12E-05	KCNAB1	7881
ENSG00000154330	184.516756	6.226354027	1.214477428	5.126776243	2.95E-07	8.43E-05	PGM5	5239
ENSG00000124212	106.1264796	5.570849451	1.089640145	5.112558928	3.18E-07	8.83E-05	PTGIS	5740
ENSG00000172403	1127.754958	4.430061757	0.866680267	5.111529507	3.20E-07	8.83E-05	SYNPO2	171024
ENSG00000177494	352.6793956	-2.632926678	0.517086338	-5.091851177	3.55E-07	9.64E-05	ZBED2	79413
ENSG00000264230	4344.871848	-1.830804549	0.360279738	-5.081619526	3.74E-07	0.00010003	ANXA8L1	728113
ENSG00000221867	218.7988039	-7.598255878	1.49638506	-5.077741072	3.82E-07	0.000100446	MAGEA3	4102
ENSG00000184613	425.3256707	-3.64894791	0.721101267	-5.060243374	4.19E-07	0.000108369	NELL2	4753
ENSG00000163017	2539.685352	5.267135606	1.050825198	5.012380379	5.38E-07	0.000136964	ACTG2	72
ENSG00000206435	150.2207054	-6.325757836	1.268963242	-4.984981147	6.20E-07	0.000155444	HLA-C	3107
ENSG00000095637	728.84793	4.028814071	0.809813054	4.974992751	6.53E-07	0.000161197	SORBS1	10580
ENSG00000162105	276.6248693	-3.870773658	0.778872299	-4.969715396	6.71E-07	0.000163175	SHANK2	22941
ENSG00000125089	1357.703447	-1.59963434	0.326055542	-4.906017936	9.29E-07	0.000222861	SH3TC1	54436
ENSG00000171766	74.02789293	3.553930904	0.725990921	4.895282847	9.82E-07	0.000231967	GATM	2628
ENSG00000114268	1179.337983	-2.178374492	0.445610538	-4.888516559	1.02E-06	0.000235661	PFKFB4	5210
ENSG00000241644	71.55777285	3.83943444	0.785714747	4.886550057	1.03E-06	0.000235661	INMT	11185
ENSG00000228892	78.7952826	-9.009091154	1.845461986	-4.88175385	1.05E-06	0.000237649	AGPAT1	10554
ENSG00000099812	310.8275931	-2.500565963	0.512472166	-4.879418101	1.06E-06	0.000237649	MISP	126353
ENSG00000241294	85.75578107	5.259015331	1.083062759	4.855688449	1.20E-06	0.000264338	IGKV2-24	28923
ENSG00000228716	940.7634126	1.53128007	0.31607317	4.844701206	1.27E-06	0.000272788	DHFR	1719
ENSG00000211890	87.95576602	5.656810454	1.167759529	4.844156964	1.27E-06	0.000272788	IGHA2	3494
ENSG00000166349	81.4024223	-4.723483556	0.983187674	-4.804254246	1.55E-06	0.000328915	RAG1	5896
ENSG00000100181	87.28425635	3.929257435	0.818642331	4.799724235	1.59E-06	0.000332129	TPTEP1	387590
ENSG00000111700	172.8451433	-6.029480718	1.260741185	-4.782488896	1.73E-06	0.000357344	SLCO1B3	28234
ENSG00000165905	761.5852187	-1.724202401	0.362002614	-4.762955659	1.91E-06	0.000388829	LARGE2	120071
ENSG00000148357	377.0662319	5.559792454	1.170269531	4.750864913	2.03E-06	0.000407722	HMCN2	256158
ENSG00000107317	284.997148	2.925653128	0.616536075	4.745307288	2.08E-06	0.000413968	PTGDS	5730
ENSG00000277015	51.54916632	6.407997195	1.352607917	4.737512709	2.16E-06	0.000425025	SALL3	27164
ENSG00000069535	194.5483557	4.885768239	1.037341339	4.709894475	2.48E-06	0.000481085	MAOB	4129
ENSG00000146453	64.48401319	5.365175337	1.147959073	4.673664299	2.96E-06	0.000567554	PNLDC1	154197

ENSG00000174175	71.58049076	5.272846532	1.130209248	4.665371958	3.08E-06	0.000584059	SELP	6403
ENSG00000166670	5057.377688	-8.410006388	1.80424745	-4.661226699	3.14E-06	0.000589098	MMP10	4319
ENSG00000111012	348.3724596	-2.582598465	0.554359917	-4.658703458	3.18E-06	0.000589587	CYP27B1	1594
ENSG00000070404	1157.541829	-1.897540035	0.410300595	-4.624755747	3.75E-06	0.000687082	FSTL3	10272
ENSG00000158292	1401.339707	-1.962068528	0.425059548	-4.615985072	3.91E-06	0.000708788	GPR153	387509
ENSG00000187715	17.64861593	8.123743257	1.761426235	4.61202581	3.99E-06	0.000714488	KBTBD12	166348
ENSG00000183311	2250.398463	-5.886748913	1.277096107	-4.609479961	4.04E-06	0.000715431	TUBB	203068
ENSG00000167676	130.5097461	3.051704663	0.664738836	4.59083252	4.41E-06	0.000771079	PLIN4	729359
ENSG00000053747	7714.538986	-2.524807781	0.550287673	-4.588159802	4.47E-06	0.000771079	LAMA3	3909
ENSG00000115008	985.9269806	-4.234680684	0.923154682	-4.587184322	4.49E-06	0.000771079	IL1A	3552
ENSG00000122367	49.73312078	3.905527928	0.858627834	4.548568975	5.40E-06	0.000917359	LDB3	11155
ENSG00000284969	357.7745965	-2.164394655	0.476183685	-4.545293594	5.49E-06	0.000922136	NA	NA
**ENSG00000055118**	38.08266237	4.274299235	0.942237638	4.536328271	5.72E-06	0.000942883	KCNH2	3757
ENSG00000132429	76.4048385	-3.969150907	0.874975303	-4.536300502	5.72E-06	0.000942883	POPDC3	64208
ENSG00000149212	1017.418902	-1.327735906	0.293161436	-4.529026472	5.93E-06	0.00096617	SESN3	143686
ENSG00000171303	30.53320753	5.404549157	1.196479883	4.517041393	6.27E-06	0.001012357	KCNK3	3777
ENSG00000156218	28.61444905	4.365547786	0.968252407	4.508687771	6.52E-06	0.001042718	ADAMTSL3	57188
ENSG00000168874	53.13264467	4.585452772	1.018578989	4.501813628	6.74E-06	0.001066569	ATOH8	84913
ENSG00000196632	21.1140734	4.515663608	1.004703423	4.49452396	6.97E-06	0.001093152	WNK3	65267
ENSG00000163239	95.269871	4.412798144	0.983457918	4.487022844	7.22E-06	0.001121556	TDRD10	126668
ENSG00000166405	63.48144469	4.524096302	1.009673604	4.480751289	7.44E-06	0.00114413	RIC3	79608
ENSG00000110841	1231.96757	-1.125389839	0.25185772	-4.468355536	7.88E-06	0.001201133	PPFIBP1	8496
ENSG00000188783	218.6146241	3.470466504	0.778290234	4.459090391	8.23E-06	0.001242625	PRELP	5549
ENSG00000173599	1312.76125	-1.996328408	0.448406434	-4.452051216	8.51E-06	0.001266361	PC	5091
ENSG00000080845	1762.252146	-1.033237989	0.232259651	-4.448633168	8.64E-06	0.001266361	DLGAP4	22839
ENSG00000162493	1920.945692	-2.46139141	0.553455318	-4.447317297	8.69E-06	0.001266361	PDPN	10630
ENSG00000185630	297.3879093	2.467336706	0.554803767	4.447224144	8.70E-06	0.001266361	PBX1	5087
ENSG00000135424	822.0454118	2.994232688	0.674448874	4.439525073	9.02E-06	0.001300903	ITGA7	3679
ENSG00000204469	310.1006073	3.575573318	0.80580351	4.437276919	9.11E-06	0.001303028	PRRC2A	7916
ENSG00000120708	14404.40532	-2.932668015	0.664346261	-4.414366705	1.01E-05	0.001426915	TGFBI	7045

ENSG00000139970	28.88291027	3.728799631	0.844782153	4.413918566	1.02E-05	0.001426915	RTN1	6252
ENSG00000124225	1933.076754	-1.903136047	0.431606778	-4.409421133	1.04E-05	0.001444418	PMEPA1	56937
ENSG00000117122	1162.227446	-2.454554039	0.559443954	-4.387488723	1.15E-05	0.001584444	MFAP2	4237
ENSG00000119782	58.95951497	-2.003556636	0.457661681	-4.377811639	1.20E-05	0.001642515	FKBP1B	2281
ENSG00000162998	52.97546373	3.016411311	0.690280959	4.369831255	1.24E-05	0.001689505	FRZB	2487
ENSG00000126218	23.37685047	4.913992654	1.125338784	4.366678482	1.26E-05	0.0016999	F10	2159
ENSG00000064115	918.867858	1.076744914	0.247036916	4.358639719	1.31E-05	0.00174909	TM7SF3	51768
ENSG00000124134	83.8903205	4.121141044	0.946764691	4.352867279	1.34E-05	0.001781202	KCNS1	3787
ENSG00000148468	83.14727863	1.781451551	0.410485904	4.33986048	1.43E-05	0.001874723	FAM171A1	221061
ENSG00000131471	445.6485846	3.47518105	0.801129451	4.337852075	1.44E-05	0.001876796	AOC3	8639
ENSG00000165810	157.5395271	4.656157661	1.074879595	4.331794631	1.48E-05	0.001913882	BTNL9	153579
ENSG00000163689	107.6299753	-3.176264699	0.733676032	-4.329246914	1.50E-05	0.00192091	CFAP20DC	200844
ENSG00000188158	194.6694649	-1.416030809	0.329597393	-4.296243954	1.74E-05	0.002212848	NHS	4810
ENSG00000166482	802.6816395	4.46343532	1.039564325	4.293563385	1.76E-05	0.002222385	MFAP4	4239
ENSG00000187068	70.7977965	3.234277837	0.754559224	4.286314092	1.82E-05	0.002278462	C3orf70	285382
ENSG00000118503	2839.061035	-1.399791866	0.328176085	-4.265368291	2.00E-05	0.002483995	TNFAIP3	7128
ENSG00000101605	66.81763946	3.835630783	0.902101961	4.251881659	2.12E-05	0.002603988	MYOM1	8736
ENSG00000282367	282.2088003	-1.771262419	0.416627198	-4.251432527	2.12E-05	0.002603988	PTDSS2	81490
ENSG00000112761	53.89825748	-5.323170346	1.253833275	-4.245516889	2.18E-05	0.002653701	CCN6	8838
ENSG00000106772	194.3139761	3.640029261	0.858523647	4.239870706	2.24E-05	0.00268248	PRUNE2	158471
ENSG00000196878	12607.65406	-2.794744763	0.659354085	-4.238609917	2.25E-05	0.00268248	LAMB3	3914
ENSG00000106034	61.54919974	3.532892758	0.833597385	4.238128408	2.25E-05	0.00268248	CPED1	79974
ENSG00000077157	1341.320588	2.483199971	0.586221102	4.235944359	2.28E-05	0.002689062	PPP1R12B	4660
ENSG00000008441	558.9297664	1.462501751	0.348275716	4.199264218	2.68E-05	0.00313572	NFIX	4784
ENSG00000099399	65.52314685	-8.350865901	1.989232899	-4.198033275	2.69E-05	0.00313572	MAGEB2	4113
ENSG00000164465	1022.946376	-2.09112106	0.498539311	-4.194495829	2.73E-05	0.003162467	DCBLD1	285761
ENSG00000065534	1658.509656	2.731403989	0.651615802	4.191739951	2.77E-05	0.003178593	MYLK	4638
ENSG00000143429	109.4303182	-1.650065455	0.394136562	-4.186532316	2.83E-05	0.003229629	LSP1P5	645166
ENSG00000196867	29.32593044	3.424375752	0.819659435	4.177803129	2.94E-05	0.003332767	ZFP28	140612
ENSG00000122861	6836.23194	-2.548353008	0.610238163	-4.175997444	2.97E-05	0.003336151	PLAU	5328

ENSG00000105290	38.21767222	2.441912177	0.586968584	4.160209326	3.18E-05	0.003550872	APLP1	333
ENSG00000173175	144.2941961	5.374270718	1.293494754	4.154845391	3.26E-05	0.003609318	ADCY5	111
ENSG00000104635	659.9137935	-1.016615471	0.24480525	-4.152751922	3.29E-05	0.003609318	SLC39A14	23516
ENSG00000163393	205.5560579	-1.767056828	0.425609367	-4.151827862	3.30E-05	0.003609318	SLC22A15	55356
ENSG00000183914	109.2448235	4.053506519	0.97711255	4.148454052	3.35E-05	0.003618935	DNAH2	146754
ENSG00000125538	1192.776658	-2.321485982	0.55964143	-4.148166767	3.35E-05	0.003618935	IL1B	3553
ENSG00000110092	2673.383146	-1.928926743	0.465294425	-4.14560467	3.39E-05	0.003635566	CCND1	595
ENSG00000178038	3473.92648	-1.624963817	0.393140351	-4.133291866	3.58E-05	0.003810938	ALS2CL	259173
ENSG00000169247	99.98227513	-1.653916404	0.400798002	-4.126558503	3.68E-05	0.00389872	SH3TC2	79628
ENSG00000161638	4093.548844	-1.63241464	0.396048939	-4.121749813	3.76E-05	0.003955335	ITGA5	3678
ENSG00000124882	298.3145965	-3.075736998	0.749409541	-4.104213824	4.06E-05	0.004227385	EREG	2069
ENSG00000203799	95.30224803	-5.159937318	1.257466364	-4.103439634	4.07E-05	0.004227385	CCDC162P	221262
ENSG00000265107	84.69170814	2.613714535	0.637798467	4.098025741	4.17E-05	0.004271598	GJA5	2702
ENSG00000172081	1114.208349	-0.998642619	0.243695859	-4.097905574	4.17E-05	0.004271598	MOB3A	126308
ENSG00000196517	390.3654759	1.804062155	0.440464615	4.095816307	4.21E-05	0.004271598	SLC6A9	6536
ENSG00000239855	164.702652	4.580209429	1.118431394	4.095208213	4.22E-05	0.004271598	IGKV1-6	28943
ENSG00000240583	921.086488	2.434120417	0.595237312	4.089327678	4.33E-05	0.004354294	AQP1	358
ENSG00000164764	74.54400927	5.196090118	1.273705978	4.079505166	4.51E-05	0.004505958	SBSPON	157869
ENSG00000274646	411.7959011	4.798333828	1.176740783	4.077647257	4.55E-05	0.004505958	RPS9	6203
ENSG00000154556	73.91493988	3.917651599	0.960922308	4.076970182	4.56E-05	0.004505958	SORBS2	8470
ENSG00000108823	50.77553639	5.009311642	1.229172888	4.075351555	4.59E-05	0.004505958	SGCA	6442
ENSG00000075618	8934.353431	-1.512268708	0.371296442	-4.072941551	4.64E-05	0.004505958	FSCN1	6624
ENSG00000026652	466.3977991	-2.050538887	0.503456808	-4.072919176	4.64E-05	0.004505958	AGPAT4	56895
ENSG00000117525	6486.948036	-2.770242065	0.681440289	-4.065274843	4.80E-05	0.00462868	F3	2152
ENSG00000066382	63.15648568	4.598417856	1.133782796	4.055819045	5.00E-05	0.004791649	MPPED2	744
ENSG00000072952	471.8730747	2.614891806	0.645805031	4.049042174	5.14E-05	0.004884137	IRAG1	10335
ENSG00000170801	427.4169661	-2.285582702	0.564534916	-4.048611761	5.15E-05	0.004884137	HTRA3	94031
ENSG00000145675	421.0743016	1.578203403	0.390153716	4.045081051	5.23E-05	0.004909876	PIK3R1	5295
ENSG00000101846	161.5489919	1.746032922	0.431686991	4.044673473	5.24E-05	0.004909876	STS	412
ENSG00000176171	829.8400116	-1.470526115	0.364120841	-4.038566187	5.38E-05	0.005003604	BNIP3	664

ENSG00000278191	269.9577736	-4.170015439	1.033102914	-4.036398875	5.43E-05	0.005003604	CARS1	833
ENSG00000138316	506.0381347	-3.016815835	0.747434063	-4.03623006	5.43E-05	0.005003604	ADAMTS14	140766
ENSG00000133135	80.1802034	-3.822094754	0.947441239	-4.034123277	5.48E-05	0.005019489	RNF128	79589
ENSG00000089820	1131.448619	3.161397982	0.783912153	4.032847267	5.51E-05	0.005019489	ARHGAP4	393
ENSG00000141639	129.8969206	4.179848648	1.039277432	4.021879546	5.77E-05	0.00522367	MAPK4	5596
ENSG00000164796	23.68217074	-5.505012503	1.369115035	-4.020854613	5.80E-05	0.00522367	CSMD3	114788
ENSG00000196793	38.82501655	3.663310485	0.912064119	4.01650543	5.91E-05	0.005291734	ZNF239	8187
ENSG00000274437	347.6098677	3.280183806	0.817057459	4.014630515	5.95E-05	0.005304826	DERL3	91319
ENSG00000072682	1257.528451	-1.646661073	0.410868841	-4.007753587	6.13E-05	0.00543196	P4HA2	8974
ENSG00000141337	48.22535987	1.97248337	0.492762706	4.002907175	6.26E-05	0.005514515	ARSG	22901
ENSG00000137033	141.373045	3.400555102	0.850107784	4.000145826	6.33E-05	0.005549263	IL33	90865
ENSG00000135423	88.66645078	4.447184877	1.114796975	3.989232994	6.63E-05	0.005779755	GLS2	27165
ENSG00000197888	54.95234629	4.610583064	1.160557666	3.972730695	7.11E-05	0.006149735	UGT2B17	7367
ENSG00000168528	4547.89441	-1.424455653	0.358628254	-3.971956022	7.13E-05	0.006149735	SERINC2	347735
ENSG00000107796	5960.049466	3.105960847	0.782423348	3.969667899	7.20E-05	0.006176409	ACTA2	59
ENSG00000152926	497.0705866	-1.98956766	0.502137674	-3.962195557	7.43E-05	0.006339629	ZNF117	51351
ENSG00000169220	569.2373745	1.492134699	0.376825799	3.959746663	7.50E-05	0.006353842	RGS14	10636
ENSG00000133063	53.05624321	4.295719097	1.08500396	3.959173657	7.52E-05	0.006353842	CHIT1	1118
ENSG00000164742	76.14406468	2.712287325	0.686557853	3.950559027	7.80E-05	0.006553002	ADCY1	107
ENSG00000137936	364.4154324	-1.514790387	0.383614761	-3.948728102	7.86E-05	0.006569454	BCAR3	8412
ENSG00000165244	143.5164167	1.965210411	0.497867026	3.947259629	7.91E-05	0.006576141	ZNF367	195828
ENSG00000079308	1832.996741	2.48073991	0.628773779	3.94536158	7.97E-05	0.006594806	TNS1	7145
ENSG00000149948	366.854821	-2.497843039	0.634095942	-3.939219408	8.17E-05	0.006731754	HMGA2	8091
ENSG00000198523	101.7939636	4.498733145	1.143978487	3.932532994	8.41E-05	0.006887055	PLN	5350
ENSG00000122642	1810.481675	-1.085836476	0.276988514	-3.920149827	8.85E-05	0.0071813	FKBP9	11328
ENSG00000106348	1511.097355	-1.346764092	0.343557153	-3.920058365	8.85E-05	0.0071813	IMPDH1	3614
ENSG00000231968	105.9679653	12.91643074	3.296158253	3.918631858	8.91E-05	0.007188165	UBD	10537
ENSG00000230034	114.7105147	12.90407835	3.296108706	3.914943196	9.04E-05	0.007262994	PSMB8	5696
ENSG00000244306	733.6476462	-2.484298932	0.634853036	-3.913187451	9.11E-05	0.007278732	DUXAP10	503639
ENSG00000067840	125.8583686	4.007459937	1.024387559	3.912054479	9.15E-05	0.007278732	PDZD4	57595

ENSG00000206208	1019.065883	-3.11074333	0.795944632	-3.908240854	9.30E-05	0.007328051	TAPBP	6892
ENSG00000274166	60.28137714	-3.208770935	0.821202374	-3.90740582	9.33E-05	0.007328051	GOLGA6L4	643707
ENSG00000072163	356.9250454	2.121724455	0.543069237	3.906913358	9.35E-05	0.007328051	LIMS2	55679
ENSG00000111961	332.9172866	1.416287659	0.362854719	3.903181035	9.49E-05	0.00740642	SASH1	23328
ENSG00000089159	4894.864805	-1.339807357	0.343659537	-3.898647394	9.67E-05	0.007510511	PXN	5829
ENSG00000137216	1381.626914	-0.887595411	0.227832486	-3.895824638	9.79E-05	0.007540572	TMEM63B	55362
ENSG00000182575	88.25875165	4.000728123	1.027043544	3.895383156	9.80E-05	0.007540572	NXPH3	11248
ENSG00000133612	1833.520856	-1.041587015	0.267549875	-3.89305737	9.90E-05	0.007577518	AGAP3	116988
ENSG00000117643	84.56240391	2.868307978	0.737508306	3.889187353	0.00010058	0.007663384	MAN1C1	57134
ENSG00000196611	12741.72805	-4.165615698	1.072798748	-3.882942356	0.0001032	0.007826403	MMP1	4312
ENSG00000152583	1662.260445	2.92392763	0.753919784	3.878300706	0.000105189	0.007940282	SPARCL1	8404
ENSG00000088826	634.5263389	-2.08531584	0.538033731	-3.875808746	0.000106271	0.007985027	SMOX	54498
ENSG00000155265	657.1493661	-2.882515432	0.743935346	-3.874685409	0.000106763	0.007985152	GOLGA7B	401647
ENSG00000161509	17.1365101	4.278351613	1.104992966	3.87183606	0.000108019	0.008032438	GRIN2C	2905
ENSG00000124440	41.43002416	4.182067323	1.08035226	3.87102196	0.00010838	0.008032438	HIF3A	64344
ENSG00000007908	160.6643712	4.064149641	1.052005932	3.863238331	0.000111894	0.008255328	SELE	6401
ENSG00000136842	87.91976377	3.790717926	0.981823247	3.860896487	0.000112972	0.008297318	TMOD1	7111
ENSG00000274577	44.41991384	-6.303440708	1.63573205	-3.853590022	0.000116398	0.008480876	LHX1	3975
ENSG00000283196	378.887903	-1.603237584	0.416063004	-3.853352903	0.000116511	0.008480876	LSP1P5	645166
ENSG00000173157	30.02942751	-7.789889517	2.022563971	-3.851492278	0.0001174	0.008507604	ADAMTS20	80070
ENSG00000019991	23.5616668	3.51829131	0.914314857	3.848008467	0.000119082	0.008591288	HGF	3082
ENSG00000267640	17.0120873	3.826366442	0.994847141	3.846185293	0.000119971	0.008617304	NA	NA
**ENSG00000101335**	4415.757966	2.992063255	0.778569425	3.843026917	0.000121526	0.008690718	MYL9	10398
ENSG00000165092	84.35352748	3.390377729	0.882748711	3.840705387	0.000122681	0.008701038	ALDH1A1	216
ENSG00000231558	110.3152019	5.026172625	1.308697205	3.840592464	0.000122738	0.008701038	HLA-DOA	3111
ENSG00000103710	277.5981035	3.295564107	0.858720687	3.837760238	0.000124162	0.008763876	RASL12	51285
ENSG00000185432	140.5630867	2.572612383	0.670761116	3.835363028	0.000125379	0.008810619	METTL7A	25840
ENSG00000172602	63.15846434	2.610399729	0.680975404	3.833324541	0.000126423	0.008810619	RND1	27289
ENSG00000277236	59.87574303	4.845642133	1.264097537	3.833281841	0.000126445	0.008810619	CCL14	6358
ENSG00000087494	5428.626962	-3.782722795	0.988584866	-3.826401684	0.00013003	0.009021872	PTHLH	5744

ENSG00000198563	655.3780917	4.30434381	1.125662883	3.823830273	0.000131394	0.009077904	DDX39B	7919
ENSG00000106089	305.3557986	-1.872718619	0.490546381	-3.817617846	0.000134746	0.009270213	STX1A	6804
ENSG00000073910	149.2689623	2.476794329	0.649702378	3.812198344	0.000137736	0.009436092	FRY	10129
ENSG00000075223	618.4642485	-1.908228408	0.500823626	-3.810180487	0.000138865	0.00947364	SEMA3C	10512
ENSG00000065717	112.9804859	2.500858106	0.657096935	3.805919601	0.000141278	0.009543597	TLE2	7089
ENSG00000143248	376.6814679	2.721496612	0.715092767	3.805795189	0.000141349	0.009543597	RGS5	8490
ENSG00000151892	59.35785298	3.803245989	0.999466809	3.805274929	0.000141647	0.009543597	GFRA1	2674
ENSG00000073578	1791.568958	-1.066791055	0.280657026	-3.801048817	0.000144085	0.009667919	SDHA	6389
ENSG00000167972	65.55446433	2.80985982	0.740760941	3.793207316	0.000148714	0.009937623	ABCA3	21
ENSG00000168477	93.46437703	5.740164209	1.513748824	3.792018938	0.000149428	0.009944554	TNXB	7148
ENSG00000144218	20.8454451	3.598500535	0.950112493	3.787446814	0.000152203	0.010088106	AFF3	3899
ENSG00000111261	182.2143801	2.4675455	0.652002258	3.784565878	0.000153977	0.010096617	MANSC1	54682
ENSG00000254681	134.2974135	2.064122479	0.545456212	3.784212984	0.000154196	0.010096617	PKD1P5	348156
ENSG00000004799	72.31735227	3.42869179	0.906197486	3.783603291	0.000154574	0.010096617	PDK4	5166
ENSG00000213859	1096.746961	-1.682639739	0.444763185	-3.783226215	0.000154809	0.010096617	KCTD11	147040
ENSG00000100297	1585.713221	0.923262054	0.244131776	3.781818443	0.000155687	0.010112838	MCM5	4174
ENSG00000175866	1538.021592	-1.314116433	0.34757227	-3.780843719	0.000156298	0.010112838	BAIAP2	10458
ENSG00000223532	3886.833893	3.711840845	0.98256938	3.777688294	0.000158291	0.010201312	HLA-B	3106
ENSG00000172830	1787.43034	-1.291418043	0.342464993	-3.770949058	0.000162628	0.010439557	SSH3	54961
ENSG00000137819	241.4656044	-2.541003298	0.674745248	-3.76587061	0.00016597	0.0106123	PAQR5	54852
ENSG00000213073	84.40063947	-1.782361442	0.4739956	-3.760291115	0.000169716	0.010809434	CHP1P2	729603
ENSG00000185627	2448.14799	-1.059487571	0.283332354	-3.739380822	0.000184474	0.011703696	PSMD13	5719
ENSG00000075213	90.70070089	-2.004566459	0.537369831	-3.730329361	0.00019123	0.012003831	SEMA3A	10371
ENSG00000175928	9.096298349	4.733189958	1.269064212	3.729669399	0.000191731	0.012003831	LRRN1	57633
ENSG00000107984	213.6981215	-2.514944383	0.674519222	-3.728499205	0.000192624	0.012003831	DKK1	22943
ENSG00000113368	819.1713744	1.554176394	0.416926417	3.727699492	0.000193236	0.012003831	LMNB1	4001
ENSG00000154914	322.60349	-1.699957287	0.456047607	-3.727587343	0.000193322	0.012003831	USP43	124739
ENSG00000169908	3819.995075	-1.636018216	0.438940779	-3.727195776	0.000193622	0.012003831	TM4SF1	4071
ENSG00000125266	1009.597712	-2.017903108	0.543418228	-3.713351896	0.000204532	0.012632186	EFNB2	1948
ENSG00000128422	137174.8091	-1.918400129	0.517535101	-3.706801963	0.000209893	0.012914356	KRT17	3872

ENSG00000144730	53.57326233	1.543318878	0.416755629	3.703174641	0.000212918	0.013051245	IL17RD	54756
ENSG00000147246	14.04114799	-6.649406597	1.796824018	-3.70064432	0.000215053	0.013086938	HTR2C	3358
ENSG00000095587	27.79179023	-3.021415964	0.816470535	-3.700581752	0.000215106	0.013086938	TLL2	7093
ENSG00000185101	1437.685065	-1.774296071	0.479977637	-3.696622375	0.000218487	0.013236782	ANO9	338440
ENSG00000186377	105.7889485	4.063446426	1.09947574	3.695803627	0.000219192	0.013236782	CYP4X1	260293
ENSG00000122756	15.46965438	3.633804889	0.983748885	3.693833807	0.000220898	0.013290573	CNTFR	1271
ENSG00000146374	37.28251072	3.618051963	0.9802901	3.690797207	0.000223552	0.013395668	RSPO3	84870
ENSG00000135069	572.4897652	2.331133228	0.631750034	3.68996138	0.000224288	0.013395668	PSAT1	29968
ENSG00000025423	45.15912631	2.91889173	0.792281902	3.684158029	0.00022946	0.013654536	HSD17B6	8630
ENSG00000052126	485.4914971	-1.035251916	0.28125723	-3.680801087	0.000232502	0.013785275	PLEKHA5	54477
ENSG00000127947	1552.061043	-1.312308943	0.356705505	-3.678970259	0.000234178	0.013834295	PTPN12	5782
ENSG00000176788	1316.169053	-2.170369138	0.590097414	-3.67798449	0.000235084	0.013837721	BASP1	10409
ENSG00000133818	405.1455253	-1.25767836	0.342400746	-3.673118046	0.000239609	0.01405331	RRAS2	22800
ENSG00000145687	180.2255067	1.470505661	0.400694568	3.669891675	0.000242653	0.014180866	SSBP2	23635
ENSG00000184564	255.5623255	-4.870036015	1.332717929	-3.654213626	0.000257971	0.015011421	SLITRK6	84189
ENSG00000144749	401.1703699	1.784991285	0.488572432	3.653483432	0.000258706	0.015011421	LRIG1	26018
ENSG00000132967	195.7864359	2.943360973	0.805876297	3.65237318	0.000259828	0.015023029	HMGB1P5	10354
ENSG00000253797	48.85715696	6.992969566	1.920632018	3.640973127	0.000271609	0.01562351	UTP14C	9724
ENSG00000236177	77.42208139	11.99784976	3.296257984	3.639839422	0.000272808	0.01562351	HLA-DPA1	3113
ENSG00000204186	62.23644217	1.601954885	0.440148845	3.639575342	0.000273088	0.01562351	ZDBF2	57683
ENSG00000205307	151.137609	1.781239993	0.489845987	3.636326598	0.000276554	0.015766459	SAP25	100316904
ENSG00000103005	1385.527446	-0.951079104	0.261682144	-3.634482238	0.000278539	0.015787517	USB1	79650
ENSG00000166922	83.60669903	-3.523446526	0.969528375	-3.634186081	0.00027886	0.015787517	SCG5	6447
ENSG00000107281	435.0985507	2.18178473	0.600744114	3.631803756	0.000281447	0.015878876	NPDC1	56654
ENSG00000083444	2499.456532	-1.2416385	0.342025175	-3.630254706	0.000283142	0.015919397	PLOD1	5351
ENSG00000054277	322.234659	-2.01251851	0.555017303	-3.62604643	0.000287794	0.016123805	OPN3	23596
ENSG00000206478	1026.721619	-7.081558619	1.953784882	-3.624533429	0.000289484	0.016123805	IER3	8870
ENSG00000115353	9.625203234	4.418503934	1.219132698	3.624301065	0.000289744	0.016123805	TACR1	6869
ENSG00000100307	346.8738793	1.998809869	0.551731824	3.622792418	0.00029144	0.016163008	CBX7	23492
ENSG00000282213	304.7862486	4.916429044	1.358438822	3.619175899	0.000295543	0.016334996	NA	NA

**ENSG00000152939**	65.62895261	3.244895606	0.896904121	3.617884598	0.000297021	0.016361229	MARVELD2	153562
ENSG00000147459	932.1994211	-1.447920874	0.400497483	-3.6153058	0.000299993	0.01644197	DOCK5	80005
ENSG00000011422	1311.803845	-1.514192299	0.418879266	-3.614865716	0.000300503	0.01644197	PLAUR	5329
ENSG00000107186	136.7132632	1.960354748	0.542784918	3.611660316	0.000304243	0.016590905	MPDZ	8777
ENSG00000010319	82.72323451	2.52935797	0.7011895	3.607238802	0.000309473	0.016819848	SEMA3G	56920
ENSG00000214694	14.18125286	2.798709651	0.777487858	3.599682775	0.000318606	0.017218155	ARHGEF33	100271715
ENSG00000002746	32.22410566	-2.773800168	0.770621698	-3.599431699	0.000318913	0.017218155	HECW1	23072
ENSG00000246596	20.69548959	3.837855819	1.06881076	3.590772066	0.0003297	0.017741771	NA	NA
**ENSG00000138735**	109.5353812	2.633919894	0.734567236	3.585675707	0.000336207	0.018032394	PDE5A	8654
ENSG00000228672	83.37810472	2.000801658	0.5583052	3.583705931	0.000338753	0.018109427	PROB1	389333
ENSG00000230373	447.1529928	-1.438195247	0.401532778	-3.581763003	0.000341283	0.018185048	GOLGA6L17P	642402
ENSG00000145936	180.2929386	2.733376378	0.765101465	3.572567172	0.000353499	0.01867823	KCNMB1	3779
ENSG00000151789	32.40069104	3.723779522	1.042386109	3.572361037	0.000353777	0.01867823	ZNF385D	79750
ENSG00000166313	111.9234991	2.239932556	0.627210973	3.571258562	0.00035527	0.01867823	APBB1	322
ENSG00000157368	52.20015466	2.388334519	0.668877052	3.570662967	0.000356079	0.01867823	IL34	146433
ENSG00000149557	763.2208827	-2.660499228	0.745128348	-3.570524776	0.000356267	0.01867823	FEZ1	9638
ENSG00000111640	64190.71488	-0.761895007	0.213905364	-3.56183217	0.000368276	0.019187502	GAPDH	2597
ENSG00000085733	7398.094057	-1.624710377	0.456185522	-3.561512364	0.000368725	0.019187502	CTTN	2017
ENSG00000125618	81.91373412	-2.22739608	0.625505572	-3.560953217	0.000369511	0.019187502	PAX8	7849
ENSG00000064205	45.11662323	2.310723034	0.652335545	3.54223076	0.000396758	0.0204766	CCN5	8839
ENSG00000233490	40.7238447	-8.483407819	2.39614639	-3.540438035	0.000399463	0.0204766	GPSM3	63940
ENSG00000232040	17.35464724	-4.582010992	1.294374936	-3.539941067	0.000400216	0.0204766	ZBED9	114821
ENSG00000197106	39.70899551	2.835615227	0.801074953	3.539762684	0.000400487	0.0204766	SLC6A17	388662
ENSG00000116285	1450.84002	-1.280106988	0.361645037	-3.53967802	0.000400615	0.0204766	ERRFI1	54206
ENSG00000121690	158.1630827	-1.522441987	0.431157319	-3.531059132	0.000413899	0.021089458	DEPDC7	91614
ENSG00000198168	40.25466533	2.688846675	0.762789409	3.525018364	0.000423453	0.02150906	SVIP	258010
ENSG00000138829	231.2881174	-2.626519599	0.746131804	-3.520181801	0.000431251	0.021767434	FBN2	2201
ENSG00000146477	583.7532978	-2.296003905	0.652300862	-3.519854161	0.000431784	0.021767434	SLC22A3	6581
ENSG00000056736	85.19074534	4.380599226	1.244705156	3.519387065	0.000432545	0.021767434	IL17RB	55540
ENSG00000078487	112.7354019	2.67712821	0.76089547	3.518391573	0.000434171	0.021782035	ZCWPW1	55063

ENSG00000165269	24.77876397	3.885998227	1.106086071	3.513287374	0.000442598	0.022136713	AQP7	364
ENSG00000159674	773.6469005	-1.995675032	0.568668257	-3.50938356	0.000449147	0.022395525	SPON2	10417
ENSG00000149582	135.828426	2.195147756	0.625715103	3.508222428	0.000451112	0.022405508	TMEM25	84866
ENSG00000124772	128.1803899	2.202523909	0.627921333	3.507643063	0.000452095	0.022405508	CPNE5	57699
ENSG00000003400	385.5332491	1.34073396	0.382603936	3.504234621	0.000457922	0.022625496	CASP10	843
ENSG00000174775	927.4724075	-1.365271784	0.39000982	-3.500608743	0.000464197	0.022866251	HRAS	3265
ENSG00000064655	124.4239295	3.775564922	1.07944719	3.497683775	0.000469317	0.022996773	EYA2	2139
ENSG00000175899	3996.717927	2.311101	0.660789415	3.497484896	0.000469667	0.022996773	A2M	2
ENSG00000211893	4161.871305	4.361994715	1.247999986	3.495188111	0.000473728	0.023072931	IGHG2	3501
ENSG00000241106	67.55347989	4.103423516	1.174082301	3.495005004	0.000474053	0.023072931	HLA-DOB	3112
ENSG00000131409	19.06966211	3.945670993	1.131326567	3.487649903	0.000487286	0.023646408	LRRC4B	94030
ENSG00000198598	158.6004908	-2.087075216	0.598824741	-3.48528555	0.000491612	0.02373361	MMP17	4326
ENSG00000213413	58.4373331	3.429398396	0.98402347	3.485077848	0.000491994	0.02373361	PVRIG	79037
ENSG00000224041	41.85284224	3.855293061	1.106590174	3.48393936	0.000494091	0.023764486	IGKV3D-15	28875
ENSG00000144671	13.21838712	-4.68089662	1.345121362	-3.479906538	0.000501589	0.02405413	SLC22A14	9389
ENSG00000164530	36.25193369	7.543976579	2.16882353	3.478372709	0.000504468	0.024121262	PI16	221476
ENSG00000108405	98.46989456	3.35624224	0.965401903	3.476523333	0.00050796	0.024217217	P2RX1	5023
ENSG00000155792	48.45323827	2.791594606	0.803233125	3.475447564	0.000510002	0.024243666	DEPTOR	64798
ENSG00000132514	37.61954829	2.784070622	0.80167291	3.472826122	0.000515009	0.024410523	CLEC10A	10462
ENSG00000165995	25.50762007	3.309956036	0.954316562	3.468404686	0.000523558	0.024743816	CACNB2	783
ENSG00000115363	150.5266339	-3.066701241	0.884815611	-3.465921262	0.000528418	0.024864549	EVA1A	84141
ENSG00000149968	3092.99788	-3.509343694	1.012639038	-3.465542569	0.000529163	0.024864549	MMP3	4314
ENSG00000162407	284.0215541	1.658744852	0.479557673	3.458905876	0.000542374	0.025249803	PLPP3	8613
ENSG00000166012	2087.617761	-1.389063426	0.401615171	-3.458692612	0.000542804	0.025249803	TAF1D	79101
ENSG00000266964	69.19584333	3.167429465	0.916089096	3.457556126	0.000545099	0.025249803	FXYD1	5348
ENSG00000118729	70.4979355	4.378732835	1.266588869	3.457106677	0.000546009	0.025249803	CASQ2	845
ENSG00000197016	36.56241663	3.040013813	0.879397086	3.456929597	0.000546368	0.025249803	ZNF470	388566
ENSG00000168209	4695.33612	-1.825197631	0.528003811	-3.456788746	0.000546653	0.025249803	DDIT4	54541
ENSG00000198691	66.55278161	-3.485346858	1.008798401	-3.454948833	0.000550397	0.025350925	ABCA4	24
ENSG00000167996	20634.12388	-0.910253124	0.263722833	-3.451552192	0.000557372	0.025532526	FTH1	2495

ENSG00000022267	1229.916389	3.15784254	0.914917879	3.451503803	0.000557472	0.025532526	FHL1	2273
ENSG00000187955	588.3296837	2.574206334	0.746010585	3.450629772	0.00055928	0.025543596	COL14A1	7373
ENSG00000234507	470.4603863	-3.46192796	1.004835275	-3.445269137	0.000570491	0.02598284	BRD2	6046
ENSG00000100350	418.5135951	2.061751618	0.599088806	3.441479123	0.000578543	0.026219708	FOXRED2	80020
ENSG00000123989	1740.182979	-1.584273232	0.460408478	-3.441016637	0.000579533	0.026219708	CHPF	79586
ENSG00000232708	33.34189661	-7.824360282	2.274559452	-3.439945381	0.000581832	0.026219708	NA	NA
**ENSG00000204315**	99.6271528	4.02431186	1.169922946	3.439809327	0.000582124	0.026219708	FKBPL	63943
ENSG00000019186	255.3881138	2.56354837	0.745559248	3.43842341	0.000585112	0.026281685	CYP24A1	1591
ENSG00000121898	189.2031917	2.964380899	0.863554728	3.432765525	0.000597459	0.026762532	CPXM2	119587
ENSG00000050820	948.4357131	-1.341805857	0.39170821	-3.425523957	0.000613615	0.02741093	BCAR1	9564
ENSG00000170271	103.3908979	2.549465004	0.744447626	3.424639846	0.000615615	0.02742514	FAXDC2	10826
ENSG00000092621	890.350912	2.473541714	0.723871241	3.417101794	0.000632916	0.028119065	PHGDH	26227
ENSG00000047634	127.7333057	1.409502479	0.41300654	3.412784889	0.000643027	0.028444957	SCML1	6322
ENSG00000171016	22.82708261	2.756661663	0.807817076	3.412482536	0.000643741	0.028444957	PYGO1	26108
ENSG00000274419	36.49943474	-8.285289466	2.431699159	-3.407201682	0.000656326	0.028922695	TBC1D3D	101060389
ENSG00000179455	41.65113919	-4.723465812	1.386908442	-3.405751719	0.000659822	0.028946273	MKRN3	7681
ENSG00000170921	1119.69402	-1.68775062	0.495634348	-3.40523337	0.000661075	0.028946273	TANC2	26115
ENSG00000100219	1957.469478	1.32225606	0.388353487	3.404774524	0.000662187	0.028946273	XBP1	7494
ENSG00000131711	491.0212931	1.812848541	0.533229917	3.399750245	0.000674474	0.029404555	MAP1B	4131
ENSG00000105976	1127.073563	-1.133575974	0.333815138	-3.395819556	0.000684235	0.029750517	MET	4233
ENSG00000170270	35.59143438	-7.435831899	2.191091818	-3.39366513	0.00068964	0.029863522	GON7	84520
ENSG00000166123	620.7649504	1.85021379	0.545250984	3.393324993	0.000690497	0.029863522	GPT2	84706
ENSG00000172348	129.9197854	2.754344299	0.813166137	3.387185194	0.000706137	0.030363422	RCAN2	10231
ENSG00000134243	404.2031546	0.995526999	0.293952663	3.386691545	0.000707409	0.030363422	SORT1	6272
ENSG00000266714	795.8572668	1.960544638	0.578912175	3.386601152	0.000707642	0.030363422	MYO15B	80022
ENSG00000174099	304.7647485	2.264117812	0.668791848	3.385384884	0.000710785	0.030418247	MSRB3	253827
ENSG00000175567	404.3128779	2.196849562	0.650028384	3.379620976	0.000725859	0.030981999	UCP2	7351
ENSG00000144837	19.3735026	3.19038887	0.944916875	3.376369873	0.000734491	0.03123112	PLA1A	51365
ENSG00000169291	71.1240636	2.844920894	0.842694202	3.37598252	0.000735526	0.03123112	SHE	126669
ENSG00000186204	51.12734085	3.314398789	0.982500157	3.373433342	0.00074237	0.031439859	CYP4F12	66002

ENSG00000160298	291.550609	1.583484813	0.469719631	3.371127602	0.000748612	0.031545721	C21orf58	54058
ENSG00000092470	225.7092051	1.370828558	0.406643653	3.371080672	0.000748739	0.031545721	WDR76	79968
ENSG00000189136	86.06999836	-1.809824257	0.53713127	-3.369426354	0.000753248	0.031653895	UBE2Q2P1	388165
ENSG00000244693	128.3253458	-5.550682099	1.648136935	-3.367852501	0.000757561	0.031753302	CTAGE8	100142659
ENSG00000166432	105.712403	3.046578575	0.905173164	3.365741158	0.000763383	0.031915284	ZMAT1	84460
ENSG00000117748	565.6239285	1.372144762	0.407871576	3.364158827	0.000767773	0.032016742	RPA2	6118
ENSG00000111452	38.82883422	3.869590381	1.150485584	3.363440999	0.000769773	0.032018232	ADGRD1	283383
ENSG00000090776	2923.158081	-1.366353553	0.406533237	-3.360988547	0.00077664	0.032097142	EFNB1	1947
ENSG00000100024	13.07408612	4.495921279	1.338003884	3.360170574	0.000778944	0.032097142	UPB1	51733
ENSG00000128268	42.42073578	2.358662523	0.702140989	3.359243457	0.000781562	0.032097142	MGAT3	4248
ENSG00000174292	461.5710435	-1.727089579	0.514159925	-3.359051327	0.000782105	0.032097142	TNK1	8711
ENSG00000100721	36.68674726	5.460409341	1.625748908	3.358703988	0.000783089	0.032097142	TCL1A	8115
ENSG00000185437	33.5796158	3.280576752	0.976779127	3.358565577	0.000783481	0.032097142	SH3BGR	6450
ENSG00000189159	3168.159574	-1.110362139	0.331869022	-3.345784225	0.000820502	0.033523492	JPT1	51155
ENSG00000141052	94.0563256	4.211244495	1.25891452	3.345139348	0.000822412	0.033523492	MYOCD	93649
ENSG00000144668	112.4104005	3.038214077	0.908435254	3.344447568	0.000824466	0.033523492	ITGA9	3680
ENSG00000116062	742.785221	0.827541017	0.247575916	3.342574799	0.00083005	0.033666573	MSH6	2956
ENSG00000123342	330.8789507	-1.293814971	0.387356512	-3.340114158	0.00083744	0.033882016	MMP19	4327
ENSG00000197937	74.12914196	2.387370196	0.715153767	3.338261371	0.000843044	0.034024341	ZNF347	84671
ENSG00000122035	87.20338168	2.507269504	0.7512371	3.337520876	0.000845294	0.034030899	RASL11A	387496
ENSG00000153012	28.29587614	3.229602328	0.967899239	3.336713366	0.000847753	0.034045856	LGI2	55203
ENSG00000112214	18.43320137	4.151820616	1.245666297	3.33301192	0.000859113	0.034417276	FHL5	9457
ENSG00000079435	61.88802411	2.298915188	0.690193269	3.330828178	0.00086588	0.034603379	LIPE	3991
ENSG00000237438	58.55230882	4.506740558	1.353982668	3.32850683	0.000873129	0.034807732	NA	NA
**ENSG00000147255**	35.85640699	-3.781591524	1.136676464	-3.326884689	0.000878227	0.034925591	IGSF1	3547
ENSG00000170873	1698.006216	-1.845174226	0.554944036	-3.32497352	0.000884269	0.03508032	MTSS1	9788
ENSG00000183798	27.07075097	3.146725098	0.947313127	3.321737035	0.00089459	0.035403601	EMILIN3	90187
ENSG00000225210	182.9014523	-2.589558575	0.782497725	-3.309349651	0.00093513	0.036918379	DUXAP9	503638
ENSG00000196263	34.89105072	2.28474148	0.690885275	3.306976661	0.000943087	0.037127868	ZNF471	57573
ENSG00000038382	1869.943692	-1.390694723	0.420605391	-3.306412025	0.00094499	0.037127868	TRIO	7204

ENSG00000285376	14.2923138	-6.857701595	2.075152105	-3.304674187	0.000950869	0.037269024	DEFB103A	414325
ENSG00000094916	1294.291319	1.259437073	0.381372637	3.302379226	0.000958684	0.037383034	CBX5	23468
ENSG00000178860	291.0036593	-1.833457689	0.555213659	-3.302256094	0.000959105	0.037383034	MSC	9242
ENSG00000238083	95.33038251	-1.336652027	0.404900095	-3.301189709	0.000962758	0.037383034	LRRC37A2	474170
ENSG00000100504	3001.853055	-1.126906927	0.34136963	-3.301134106	0.000962948	0.037383034	PYGL	5836
ENSG00000125845	278.4113653	-2.682425025	0.813179727	-3.298686544	0.000971383	0.037570283	BMP2	650
ENSG00000128641	3226.105828	-1.469273906	0.445572128	-3.297499585	0.000975498	0.037570283	MYO1B	4430
ENSG00000131746	7530.564709	-1.621698307	0.491808812	-3.297416127	0.000975788	0.037570283	TNS4	84951
ENSG00000105971	1392.15236	-1.073352236	0.325547219	-3.297070816	0.000976989	0.037570283	CAV2	858
ENSG00000111341	731.7314374	2.772448575	0.841307482	3.295404635	0.000982801	0.037620609	MGP	4256
ENSG00000138166	835.3023671	-1.273157941	0.386347167	-3.295372786	0.000982912	0.037620609	DUSP5	1847
ENSG00000197747	10021.93072	-1.296591141	0.39371344	-3.293235665	0.000990414	0.037818978	S100A10	6281
ENSG00000196159	72.96725649	2.440018921	0.741915028	3.288811831	0.001006113	0.03832866	FAT4	79633
ENSG00000172935	204.0184039	2.341986755	0.712431111	3.287316795	0.00101147	0.038404883	MRGPRF	116535
ENSG00000176046	787.0536265	1.934052365	0.588405139	3.286939959	0.001012824	0.038404883	NUPR1	26471
ENSG00000074527	256.5003081	1.785214723	0.543821193	3.282723709	0.001028094	0.038893431	NTN4	59277
ENSG00000154553	346.8883477	2.739060516	0.834910084	3.28066527	0.001035626	0.039087679	PDLIM3	27295
ENSG00000120693	37.25897405	2.237814092	0.682451949	3.27907935	0.001041463	0.039217233	SMAD9	4093
ENSG00000138311	80.3526509	-3.240312056	0.989759305	-3.273838436	0.001060973	0.03985981	ZNF365	22891
ENSG00000203995	49.33683268	4.920642952	1.503680739	3.272398737	0.001066391	0.039971263	ZYG11A	440590
ENSG00000110427	298.2468599	-2.941955996	0.899247406	-3.271575737	0.001069499	0.039995843	KIAA1549L	25758
ENSG00000235220	557.6525981	-2.668295921	0.816082111	-3.269641478	0.001076839	0.040178159	HLA-F	3134
ENSG00000017427	22.78780584	3.372315102	1.031961199	3.267870056	0.001083601	0.040338162	IGF1	3479
ENSG00000078596	79.75825622	3.396599834	1.041758815	3.260447414	0.001112366	0.041290152	ITM2A	9452
ENSG00000137642	937.0734207	-1.978968273	0.60712128	-3.259592998	0.001115722	0.041290152	SORL1	6653
ENSG00000087301	55.17083043	1.551330586	0.475966637	3.259326314	0.001116771	0.041290152	TXNDC16	57544
ENSG00000109610	426.5186235	2.974266817	0.913143803	3.257172427	0.001125281	0.041510632	SOD3	6649
ENSG00000187513	75.73193943	1.998472647	0.614186259	3.253854377	0.001138506	0.041903712	GJA4	2701
ENSG00000105329	1689.370715	-1.62166079	0.498663355	-3.25201516	0.001145899	0.042080822	TGFB1	7040
ENSG00000184363	4776.839739	-1.385175729	0.426043868	-3.251251417	0.001148982	0.042099218	PKP3	11187

ENSG00000198046	46.22274002	3.353127752	1.031634554	3.250305778	0.00115281	0.042144763	ZNF667	63934
ENSG00000151348	1306.82426	-1.067125888	0.328539688	-3.248088212	0.001161832	0.042379593	EXT2	2132
ENSG00000185038	46.42826556	4.570189929	1.407770446	3.246402808	0.001168733	0.042536159	MROH2A	339766
ENSG00000174348	156.7052546	2.873534516	0.886260794	3.242312572	0.001185639	0.043055334	PODN	127435
ENSG00000131831	37.99658669	2.720933721	0.839561552	3.240898437	0.001191536	0.043084929	RAI2	10742
ENSG00000158169	240.8410229	1.243315542	0.383638723	3.240849962	0.001191739	0.043084929	FANCC	2176
ENSG00000115468	49.05551471	2.470646202	0.763061931	3.237805613	0.001204529	0.043385144	EFHD1	80303
ENSG00000145365	138.1035419	1.234820609	0.381457097	3.237115312	0.001207446	0.043385144	TIFA	92610
ENSG00000135476	537.3874377	0.932684378	0.28822713	3.235935421	0.001212448	0.043385144	ESPL1	9700
ENSG00000133466	765.8004917	-1.135768959	0.350991411	-3.235888185	0.001212649	0.043385144	C1QTNF6	114904
ENSG00000016391	57.43329674	2.553553118	0.789175237	3.235723825	0.001213347	0.043385144	CHDH	55349
ENSG00000121769	61.95925726	3.162547877	0.97767655	3.234758854	0.001217455	0.043436772	FABP3	2170
ENSG00000053702	17.96805823	3.147466708	0.974074338	3.231238711	0.00123255	0.043879303	NRIP2	83714
ENSG00000133816	1552.656667	-1.40080575	0.433952585	-3.22801568	0.001246521	0.044280025	MICAL2	9645
ENSG00000131620	4225.352048	-2.765845523	0.857028216	-3.227251417	0.001249856	0.044301957	ANO1	55107
ENSG00000211947	649.13358	7.928892266	2.459810854	3.223374778	0.001266897	0.044651055	IGHV3-21	28444
ENSG00000125912	1794.914956	-0.866061934	0.268693435	-3.223234441	0.001267518	0.044651055	NCLN	56926
ENSG00000265190	6262.603373	-1.393173346	0.432240558	-3.223143499	0.00126792	0.044651055	ANXA8	653145
ENSG00000285213	95.86064003	-9.643578489	2.992641311	-3.222430451	0.00127108	0.044665868	NA	NA
**ENSG00000275395**	346.4980256	3.7906173	1.178184646	3.217337209	0.001293864	0.045368725	FCGBP	8857
ENSG00000101333	36.23155275	2.762735499	0.859124015	3.215758668	0.001301002	0.045521107	PLCB4	5332
ENSG00000100311	327.0999218	-1.143406339	0.355697801	-3.214544301	0.001306518	0.045616206	PDGFB	5155
ENSG00000176148	277.8038021	-1.195728082	0.3720842	-3.213595426	0.001310842	0.04566941	TCP11L1	55346
ENSG00000123612	29.47152384	-2.218273896	0.69073607	-3.211463818	0.001320606	0.045911473	ACVR1C	130399
ENSG00000147526	665.0355352	1.230407162	0.383304631	3.209998169	0.001327358	0.046047839	TACC1	6867
ENSG00000136295	1927.578275	-1.490495299	0.46441724	-3.209388392	0.001330177	0.046047839	TTYH3	80727
ENSG00000184208	436.4266751	0.894450773	0.279051821	3.2053214	0.001349117	0.046511479	C22orf46	79640
ENSG00000196616	40.27038561	7.240820026	2.259023378	3.205287779	0.001349275	0.046511479	ADH1B	125
ENSG00000163235	1012.562715	-1.6099607	0.502861165	-3.201600783	0.001366663	0.046952976	TGFA	7039
ENSG00000006459	764.3485241	-1.45350542	0.45402858	-3.201352259	0.001367842	0.046952976	KDM7A	80853

ENSG00000241749	29.5046992	-3.489562741	1.090442161	-3.200135566	0.00137363	0.047052586	RPSAP52	204010
ENSG00000277318	114.6697826	3.931019689	1.229791766	3.196492119	0.001391096	0.047550995	NA	NA
**ENSG00000170629**	33.04465463	2.713793699	0.849301651	3.195323708	0.001396741	0.047595624	DPY19L2P2	349152
ENSG00000108924	36.87128086	2.889815754	0.904476679	3.195014112	0.00139824	0.047595624	HLF	3131
ENSG00000224389	349.445928	2.438525221	0.76396379	3.191938221	0.001413216	0.047940589	C4B	721
ENSG00000112118	1809.856695	0.815998461	0.25566057	3.191725897	0.001414255	0.047940589	MCM3	4172
ENSG00000187243	388.501891	-1.861431022	0.584366823	-3.18538108	0.001445635	0.048847046	MAGED4B	81557
ENSG00000133083	43.59143993	2.866707206	0.900322791	3.184088235	0.001452107	0.048847046	DCLK1	9201
ENSG00000172915	15.08768231	3.271903641	1.027615094	3.183977793	0.001452662	0.048847046	NBEA	26960
ENSG00000170017	688.6900198	-1.802610516	0.566251635	-3.183408939	0.001455519	0.048847046	ALCAM	214
ENSG00000147027	79.7634264	3.291714006	1.034051161	3.183318322	0.001455975	0.048847046	TMEM47	83604
ENSG00000163536	44.31256632	2.312080265	0.72774647	3.177040851	0.001487861	0.049814312	SERPINI1	5274
ENSG00000183287	86.29764649	-2.793861857	0.879644892	-3.176124688	0.001492568	0.049869505	CCBE1	147372
ENSG00000172346	76.61619187	3.387656452	1.066812113	3.175494926	0.001495811	0.049875674	CSDC2	27254
ENSG00000233564	38.52595583	4.205526297	1.326186799	3.171141728	0.00151841	0.050470826	PRR3	80742
ENSG00000106018	24.87900223	3.625426465	1.143460673	3.170573812	0.001521382	0.050470826	VIPR2	7434
ENSG00000057294	310.9961392	-1.752579083	0.552816088	-3.170275107	0.001522947	0.050470826	PKP2	5318
ENSG00000140479	167.145153	-1.249405661	0.39443481	-3.167584678	0.001537109	0.050696447	PCSK6	5046
ENSG00000182472	345.7405892	-2.213210321	0.698798159	-3.16716679	0.00153932	0.050696447	CAPN12	147968
ENSG00000119686	163.0096827	2.243890747	0.708505381	3.167076508	0.001539798	0.050696447	FLVCR2	55640
ENSG00000159374	26.39792688	2.892306303	0.913371975	3.166624752	0.001542192	0.050696447	M1AP	130951
ENSG00000186638	193.5997362	1.359112705	0.429629135	3.163455631	0.001559081	0.051148527	KIF24	347240
ENSG00000006453	1592.750599	-0.973072141	0.307837943	-3.160988314	0.001572348	0.051439648	BAIAP2L1	55971
ENSG00000171067	932.7470913	-1.09276902	0.345743671	-3.160633473	0.001574265	0.051439648	C11orf24	53838
ENSG00000110203	35.20868829	-3.854499909	1.219911207	-3.159656118	0.001579555	0.051507034	FOLR3	2352
ENSG00000197361	58.52825622	3.765202458	1.191864295	3.159086545	0.001582645	0.051507034	FBXL22	283807
ENSG00000137960	26.45751498	4.670162355	1.478921061	3.157817194	0.001589552	0.051628776	GIPC2	54810
ENSG00000075073	23.44699924	3.712770098	1.176590408	3.155533201	0.00160205	0.051836367	TACR2	6865
ENSG00000141232	606.3062	-1.058748681	0.335526192	-3.155487432	0.001602302	0.051836367	TOB1	10140
ENSG00000101443	226.4364633	3.585985916	1.137012112	3.153867826	0.00161122	0.052021681	WFDC2	10406

ENSG00000143507	298.8052595	-2.261954186	0.717500937	-3.152545271	0.001618537	0.052057332	DUSP10	11221
ENSG00000120802	1086.261292	0.941505581	0.298652298	3.152514099	0.00161871	0.052057332	TMPO	7112
ENSG00000007968	326.4818255	2.072893521	0.657779641	3.151349465	0.001625179	0.052095369	E2F2	1870
ENSG00000107954	74.10058075	3.165528524	1.00456257	3.151151175	0.001626283	0.052095369	NEURL1	9148
ENSG00000075673	55.07140539	-3.957400874	1.25668161	-3.149087918	0.001637809	0.052358404	ATP12A	479
ENSG00000197380	99.72909071	2.614519126	0.830392545	3.148533959	0.001640917	0.052358404	DACT3	147906
ENSG00000206341	116.0523991	3.564749588	1.132700733	3.147123935	0.001648851	0.052437729	NA	NA
**ENSG00000179431**	352.8577138	-1.44788457	0.460091458	-3.146949469	0.001649835	0.052437729	FJX1	24147
ENSG00000135929	106.4422176	2.17654666	0.692201209	3.144384367	0.001664367	0.052794835	CYP27A1	1593
ENSG00000173156	1755.476208	-1.757100414	0.558949076	-3.143578709	0.001668955	0.052794835	RHOD	29984
ENSG00000186493	80.67442103	-1.391919585	0.442827001	-3.143258163	0.001670784	0.052794835	C5orf38	153571
ENSG00000068976	46.36408251	2.865530302	0.912111376	3.141645172	0.001680015	0.052983839	PYGM	5837
ENSG00000125731	729.447165	-1.485638597	0.473393632	-3.138273303	0.001699463	0.053493726	SH2D3A	10045
ENSG00000105875	1349.359331	-1.112883365	0.354977597	-3.135080563	0.001718069	0.053867207	WDR91	29062
ENSG00000135919	1210.48275	-2.543115094	0.811263306	-3.134759178	0.001719952	0.053867207	SERPINE2	5270
ENSG00000109046	3763.562143	-0.923602237	0.29465323	-3.134539667	0.00172124	0.053867207	WSB1	26118
ENSG00000205076	831.1873593	-3.940590292	1.25752062	-3.133618828	0.00172665	0.053933002	LGALS7	3963
ENSG00000145362	57.83422398	4.040451202	1.290263049	3.131494159	0.001739192	0.054220904	ANK2	287
ENSG00000196126	1567.394346	3.343476406	1.068908517	3.12793504	0.001760391	0.054777047	HLA-DRB1	3123
ENSG00000165795	478.2124929	1.344924471	0.430101036	3.126996583	0.00176602	0.054827352	NDRG2	57447
ENSG00000177697	4788.829827	-1.275568733	0.407980254	-3.126545268	0.001768733	0.054827352	CD151	977
ENSG00000139194	20.82778445	2.917753593	0.933480234	3.125672604	0.001773989	0.05488595	RBP5	83758
ENSG00000187957	36.66563517	-2.75190434	0.881371605	-3.122297479	0.001794455	0.055377907	DNER	92737
ENSG00000162804	111.9082614	1.734060986	0.555504597	3.121596102	0.001798735	0.055377907	SNED1	25992
ENSG00000011347	584.4203877	-2.999806498	0.961052531	-3.1213762	0.001800079	0.055377907	SYT7	9066
ENSG00000196581	270.2104859	-3.500537974	1.122154222	-3.119480287	0.001811704	0.055630567	AJAP1	55966
ENSG00000165821	26.27878427	3.082493314	0.989322869	3.115760699	0.001834711	0.056158489	SALL2	6297
ENSG00000176890	588.2453641	0.893340218	0.286732453	3.115588103	0.001835785	0.056158489	TYMS	7298
ENSG00000269404	26.66130257	4.057150882	1.302508761	3.114874161	0.001840234	0.056189167	SPIB	6689
ENSG00000183780	78.49066352	-3.394775931	1.090407019	-3.113310783	0.001850011	0.056300891	SLC35F3	148641

ENSG00000211611	30.43737846	5.683423459	1.825906416	3.112658682	0.001854103	0.056300891	IGKV6-21	28906
ENSG00000122783	603.116551	-0.778966826	0.250266871	-3.112544712	0.001854819	0.056300891	CYREN	78996
ENSG00000106258	57.2274749	-2.303825579	0.740283437	-3.112085807	0.001857705	0.056300891	CYP3A5	1577
ENSG00000182985	44.20645166	1.890950031	0.60812421	3.109479938	0.00187417	0.056694515	CADM1	23705
ENSG00000137312	187.7795158	-10.66362145	3.434448548	-3.104900626	0.00190343	0.057419256	FLOT1	10211
ENSG00000124731	280.4578615	-2.724187526	0.877459616	-3.104630091	0.001905171	0.057419256	TREM1	54210
ENSG00000137745	1599.364812	-4.033961183	1.299855252	-3.10339261	0.001913157	0.057553538	MMP13	4322
ENSG00000211945	14.9810692	4.552830329	1.467585978	3.102257991	0.001920505	0.057661705	IGHV1-18	28468
ENSG00000129521	749.6326452	-1.490567108	0.48055733	-3.101746689	0.001923825	0.057661705	EGLN3	112399
ENSG00000133019	58.48289586	-3.651934621	1.177816584	-3.100597047	0.001931309	0.057779809	CHRM3	1131
ENSG00000157680	36.93430784	-2.493924646	0.804872649	-3.098533225	0.001944812	0.057861889	DGKI	9162
ENSG00000204764	24.32938108	4.792768903	1.546923313	3.098258888	0.001946613	0.057861889	RANBP17	64901
ENSG00000165140	174.9756052	2.948022097	0.951549808	3.098126943	0.00194748	0.057861889	FBP1	2203
ENSG00000234906	62.16512244	3.128965602	1.01012238	3.097610413	0.001950877	0.057861889	APOC2	344
ENSG00000164985	402.717575	1.398222038	0.451407659	3.097470788	0.001951796	0.057861889	PSIP1	11168
ENSG00000117632	2568.487633	1.093989737	0.3533029	3.096464074	0.001958436	0.057953353	STMN1	3925
ENSG00000170153	33.27879366	3.303873712	1.068586294	3.091817414	0.001989352	0.058691503	RNF150	57484
ENSG00000151376	121.2342872	1.769430157	0.572328428	3.091634227	0.00199058	0.058691503	ME3	10873
ENSG00000174996	991.8318746	-1.252011904	0.405247715	-3.089497749	0.002004952	0.059008568	KLC2	64837
ENSG00000211959	230.9013615	4.859919223	1.573504967	3.088594777	0.002011055	0.059081545	IGHV4-39	28394
ENSG00000157110	551.8542704	2.131024445	0.690484045	3.086276157	0.002026805	0.059272874	RBPMS	11030
ENSG00000169994	167.357517	-3.566521894	1.155666829	-3.086116002	0.002027897	0.059272874	MYO7B	4648
ENSG00000177731	2702.888474	-0.840256989	0.272308994	-3.085674757	0.002030908	0.059272874	FLII	2314
ENSG00000132840	29.66956216	3.135001013	1.01612687	3.085245658	0.002033841	0.059272874	BHMT2	23743
ENSG00000118160	44.68445073	3.717952833	1.205183807	3.084967463	0.002035744	0.059272874	SLC8A2	6543
ENSG00000125531	26.032667	-2.812842149	0.913198481	-3.080208967	0.002068554	0.060120809	FNDC11	79025
ENSG00000132016	130.0565309	2.532336646	0.823149917	3.076397862	0.002095181	0.060745224	BRME1	79173
ENSG00000133619	518.5215423	-1.521143365	0.49450877	-3.076069542	0.002097489	0.060745224	KRBA1	84626
ENSG00000089327	2266.350468	-1.363337083	0.443358182	-3.075024078	0.002104856	0.06085048	FXYD5	53827
ENSG00000183172	303.0460372	0.852699265	0.277494168	3.072854718	0.002120217	0.06118608	SMDT1	91689

ENSG00000227372	282.4354326	1.698736171	0.552921994	3.072289017	0.002124239	0.061193858	TP73-AS1	57212
ENSG00000026508	19634.62255	-1.374938225	0.447642206	-3.071511595	0.002129779	0.061213212	CD44	960
ENSG00000144712	42.87718495	3.191445779	1.039172454	3.071141625	0.00213242	0.061213212	CAND2	23066
ENSG00000105369	186.2838702	3.250848528	1.059722295	3.067641913	0.00215755	0.061825748	CD79A	973
ENSG00000061455	23.26177156	3.213651925	1.048576645	3.064775418	0.002178335	0.062311847	PRDM6	93166
ENSG00000172724	122.9655647	3.601249258	1.175259535	3.064216158	0.002182412	0.062319126	CCL19	6363
ENSG00000275365	258.2443749	-10.51850224	3.434546078	-3.062559653	0.002194527	0.062555538	MMP11	4320
ENSG00000134013	1102.839855	-1.553686002	0.507403779	-3.062030809	0.002198408	0.0625568	LOXL2	4017
ENSG00000112294	158.7312959	2.207856179	0.721296146	3.060956573	0.002206311	0.062672299	ALDH5A1	7915
ENSG00000104327	85.67804153	-5.230409099	1.709687183	-3.059278417	0.002218708	0.062914856	CALB1	793
ENSG00000149927	52.33030322	3.611616273	1.180808007	3.058597376	0.002223758	0.062948565	DOC2A	8448
ENSG00000134333	19071.58893	-1.196558434	0.391352676	-3.057493937	0.002231962	0.063015966	LDHA	3939
ENSG00000100612	714.2549522	-1.161684116	0.379978311	-3.057237956	0.002233869	0.063015966	DHRS7	51635
ENSG00000169946	33.07148176	2.555169445	0.836045272	3.056257274	0.002241189	0.063113264	ZFPM2	23414
ENSG00000094963	23.91976148	3.257919544	1.066455795	3.054903502	0.002251329	0.063289529	FMO2	2327
ENSG00000115009	356.4784031	-3.472339538	1.138178818	-3.050785592	0.002282435	0.064053533	CCL20	6364
ENSG00000165507	717.5023573	2.236299328	0.733374974	3.049325935	0.002293555	0.064255007	DEPP1	11067
ENSG00000078098	589.7283333	-2.657965676	0.871854046	-3.048636052	0.002298828	0.064292264	FAP	2191
ENSG00000083807	36.74583712	2.532829654	0.834265479	3.035999594	0.002397397	0.066934171	SLC27A5	10998
ENSG00000281123	29.44621776	5.536421665	1.824057298	3.035223548	0.002403575	0.066991938	FCGBP	8857
ENSG00000174080	210.0773264	1.599623655	0.528114333	3.028934372	0.00245418	0.068253956	CTSF	8722
ENSG00000175305	126.7603187	1.713344819	0.565729275	3.028559588	0.002457226	0.068253956	CCNE2	9134
ENSG00000095209	117.2547266	1.724359343	0.569689656	3.026839831	0.002471249	0.068526717	TMEM38B	55151
ENSG00000081052	21.61167998	2.833129215	0.936642082	3.024772504	0.002488202	0.068879686	COL4A4	1286
ENSG00000205089	32.97892988	3.294462857	1.091664661	3.017834115	0.002545882	0.070356966	CCNI2	645121
ENSG00000084652	1585.857882	0.915606428	0.3034528	3.017294381	0.00255042	0.07036311	TXLNA	200081
ENSG00000185070	814.1583038	-2.494417519	0.827369907	-3.01487581	0.002570845	0.070806803	FLRT2	23768
ENSG00000039560	1269.130908	-1.678891142	0.557294059	-3.012576782	0.002590399	0.071150031	RAI14	26064
ENSG00000249915	1607.008728	-0.729334191	0.242111859	-3.012385237	0.002592034	0.071150031	PDCD6	10016
ENSG00000121577	120.3921124	2.199138557	0.730580084	3.010126616	0.002611388	0.07156081	POPDC2	64091

ENSG00000276536	761.5389053	-1.602734275	0.532538055	-3.009614541	0.002615794	0.071561285	HRAS	3265
ENSG00000145555	1653.177361	-1.364867623	0.454559317	-3.0026172	0.002676689	0.072976543	MYO10	4651
ENSG00000148848	417.8331075	-2.031069611	0.676544604	-3.002122254	0.002681045	0.072976543	ADAM12	8038
ENSG00000125821	367.2671917	-0.905422164	0.301628531	-3.001778914	0.00268407	0.072976543	DTD1	92675
ENSG00000151729	161.0699404	1.737795222	0.579046318	3.001133359	0.002689767	0.072976543	SLC25A4	291
ENSG00000177951	957.9454078	-0.749400714	0.2497072	-3.001117769	0.002689905	0.072976543	BET1L	51272
ENSG00000163710	69.00503486	3.532328366	1.177700086	2.999344576	0.002705611	0.073201191	PCOLCE2	26577
ENSG00000135437	102.369659	2.235436638	0.745351829	2.999169723	0.002707165	0.073201191	RDH5	5959
ENSG00000173077	55.42361885	-3.086282513	1.029512806	-2.997808766	0.002719282	0.073317995	DELEC1	50514
ENSG00000187689	86.91511094	-4.055661654	1.352935813	-2.997674845	0.002720478	0.073317995	AMTN	401138
ENSG00000157470	16.0819974	2.972586203	0.992055914	2.996389781	0.00273197	0.073506217	FAM81A	145773
ENSG00000196935	342.158186	-1.57376433	0.527241513	-2.984902155	0.00283669	0.076198085	SRGAP1	57522
ENSG00000076604	1356.532479	-0.949839184	0.31828045	-2.984283776	0.00284243	0.076226685	TRAF4	9618
ENSG00000155962	89.60140951	1.945478585	0.652592618	2.981153222	0.002871651	0.076811895	CLIC2	1193
ENSG00000166803	273.6800755	0.977642476	0.327964765	2.98093753	0.002873674	0.076811895	PCLAF	9768
ENSG00000063660	3966.906419	-1.384550548	0.464727618	-2.979273224	0.00288933	0.076928095	GPC1	2817
ENSG00000243264	52.46694768	4.042044636	1.35696313	2.978743156	0.002894333	0.076928095	IGKV2D-29	28882
ENSG00000113296	76.58462828	3.737241017	1.254693103	2.978609677	0.002895594	0.076928095	THBS4	7060
ENSG00000122884	542.3839026	-1.446378665	0.485610942	-2.978472145	0.002896894	0.076928095	P4HA1	5033
ENSG00000102890	925.8553314	-1.46508326	0.492172231	-2.976769445	0.00291303	0.077230815	ELMO3	79767
ENSG00000280759	382.7535733	-3.109091494	1.044926049	-2.97541773	0.002925898	0.077446055	AP2A2	161
ENSG00000173621	598.817383	-1.236160827	0.415604456	-2.974368559	0.002935922	0.077585429	LRFN4	78999
ENSG00000085063	10767.28457	-1.539521924	0.517901134	-2.972617403	0.002952723	0.077903143	CD59	966
ENSG00000151065	128.3884775	1.628006996	0.547858984	2.971580362	0.002962713	0.078040452	DCP1B	196513
ENSG00000171603	5356.137513	-0.930760165	0.313647286	-2.967537763	0.003001954	0.07894655	CLSTN1	22883
ENSG00000068831	135.9133232	2.266039275	0.763838432	2.966647371	0.00301066	0.079048015	RASGRP2	10235
ENSG00000161714	769.9602394	-1.385046483	0.467051719	-2.965509871	0.003021816	0.079209168	PLCD3	113026
ENSG00000149591	8903.048121	2.617118658	0.882661172	2.965032041	0.003026514	0.079209168	TAGLN	6876
ENSG00000174851	1231.066693	-1.25134329	0.422220247	-2.963721659	0.003039431	0.07941974	YIF1A	10897
ENSG00000224081	131.756262	-2.316256451	0.781906788	-2.96231787	0.003053324	0.079532591	SLC44A3-AS1	101928079

ENSG00000258311	101.2805931	1.914117332	0.646159267	2.96229959	0.003053505	0.079532591	NA	NA
**ENSG00000176533**	42.09008564	2.789205259	0.942095443	2.960639795	0.003070008	0.079816358	GNG7	2788
ENSG00000185010	47.84655717	1.767657398	0.597137092	2.960220395	0.00307419	0.079816358	F8	2157
ENSG00000150687	1974.903572	-1.138890788	0.386070264	-2.949957289	0.003178179	0.082385059	PRSS23	11098
ENSG00000144647	124.9179725	1.679493846	0.569480015	2.949170825	0.003186278	0.08246391	POMGNT2	84892
ENSG00000138135	41.24300443	1.726568028	0.587074095	2.94097124	0.003271849	0.084473197	CH25H	9023
ENSG00000134369	2147.498607	-1.661485504	0.564988607	-2.940741608	0.003274275	0.084473197	NAV1	89796
ENSG00000136840	260.755057	1.433932854	0.487978107	2.938518827	0.003297846	0.084946894	ST6GALNAC4	27090
ENSG00000278082	219.8215798	3.458008454	1.177423969	2.936927177	0.003314819	0.085249415	NA	NA
**ENSG00000076003**	897.9903582	1.172801458	0.399470242	2.935891924	0.003325902	0.085253579	MCM6	4175
ENSG00000177426	1270.515108	-1.205294325	0.410658225	-2.935030279	0.003335151	0.085253579	TGIF1	7050
ENSG00000241351	949.0982094	3.831820204	1.305561711	2.934997382	0.003335505	0.085253579	IGKV3-11	28914
ENSG00000177943	347.5675589	1.368444248	0.466256363	2.934961017	0.003335896	0.085253579	MAMDC4	158056
ENSG00000225697	892.7899037	-1.02028218	0.347792525	-2.933594332	0.003350618	0.085495821	SLC26A6	65010
ENSG00000134533	45.7250316	2.660078705	0.906949529	2.9329953	0.00335709	0.085527107	RERG	85004
ENSG00000115590	891.0118843	-2.995254353	1.021682648	-2.931687602	0.003371257	0.085633957	IL1R2	7850
ENSG00000139722	1458.881655	-0.993567388	0.338985255	-2.931004732	0.003378676	0.085633957	VPS37B	79720
ENSG00000183578	86.22670722	1.992664074	0.679908437	2.93078298	0.003381089	0.085633957	TNFAIP8L3	388121
ENSG00000275873	56.90694499	-2.526717137	0.862162921	-2.930672468	0.003382292	0.085633957	LMNTD2	256329
ENSG00000186831	83.06960239	3.945845511	1.346669406	2.930077341	0.003388776	0.085665117	NA	NA
**ENSG00000144681**	41.78512227	-3.121945143	1.06644699	-2.927426467	0.003417799	0.08619924	STAC	6769
ENSG00000167291	566.715619	-1.031824095	0.352497334	-2.927182694	0.003420479	0.08619924	TBC1D16	125058
ENSG00000176597	846.6909358	-1.44783319	0.494864523	-2.925716277	0.003436642	0.086242256	B3GNT5	84002
ENSG00000143067	108.2339548	-1.431473285	0.48928455	-2.925645796	0.003437421	0.086242256	ZNF697	90874
ENSG00000132000	174.5161171	-1.54840733	0.529263542	-2.925588495	0.003438054	0.086242256	PODNL1	79883
ENSG00000188157	7675.955033	-1.049938395	0.359149449	-2.923402497	0.003462286	0.086716713	AGRN	375790
ENSG00000185761	293.3946466	-1.642813023	0.562263537	-2.921784742	0.00348032	0.08687642	ADAMTSL5	339366
ENSG00000282211	190.4613196	3.98167902	1.362837066	2.921610455	0.003482268	0.08687642	NA	NA
**ENSG00000168309**	40.91773257	2.853477843	0.976750923	2.921397643	0.003484648	0.08687642	FAM107A	11170
ENSG00000150594	48.14079279	3.631233513	1.243285819	2.920674763	0.003492742	0.08694529	ADRA2A	150

ENSG00000214530	887.91805	-1.358740202	0.465394967	-2.919542108	0.00350546	0.087128858	STARD10	10809
ENSG00000139549	7.878560987	4.975411492	1.704963057	2.918193137	0.003520662	0.087279979	DHH	50846
ENSG00000112715	7734.697039	-1.590146448	0.544934078	-2.918052863	0.003522246	0.087279979	VEGFA	7422
ENSG00000153558	133.0617124	-1.629145848	0.558871987	-2.915060848	0.003556192	0.087864504	FBXL2	25827
ENSG00000078124	434.1881751	-1.241806389	0.426002127	-2.915023918	0.003556613	0.087864504	ACER3	55331
ENSG00000198208	35.40578716	2.951166249	1.012649395	2.91430209	0.003564848	0.087934705	RPS6KL1	83694
ENSG00000274538	329.4528304	-1.401277932	0.480930201	-2.913682544	0.003571929	0.087976296	PHLDA2	7262
ENSG00000133398	664.0037821	-1.189130941	0.408366443	-2.911921296	0.003592131	0.088258208	MED10	84246
ENSG00000120051	27.68251816	-2.09373522	0.719066348	-2.911741353	0.003594201	0.088258208	CFAP58	159686
ENSG00000159176	4702.962325	1.614990816	0.554868855	2.910581124	0.003607573	0.08845335	CSRP1	1465
ENSG00000276230	2658.093128	-0.695903851	0.239173321	-2.909621565	0.003618666	0.088592123	RNH1	6050
ENSG00000101384	7216.042822	-1.55715582	0.535380529	-2.908502899	0.003631638	0.0887764	JAG1	182
ENSG00000233192	8.032597087	6.974968847	2.398521636	2.908028321	0.003637154	0.088778137	HLA-DQA1	3117
ENSG00000189423	89.05526	-1.426094923	0.490860567	-2.905295348	0.003669067	0.089423225	NA	NA
**ENSG00000274276**	94.09085682	4.455797614	1.534559399	2.903633197	0.0036886	0.089765117	CBSL	102724560
ENSG00000070808	63.92162906	-1.893360596	0.652260824	-2.90276608	0.003698828	0.089879867	CAMK2A	815
ENSG00000197712	722.4751505	-1.169882958	0.403243011	-2.901185947	0.003717532	0.090199941	FAM114A1	92689
ENSG00000143476	302.6767375	1.05804303	0.365078702	2.898123129	0.003754032	0.090851897	DTL	51514
ENSG00000238105	277.8979809	2.316865381	0.799471381	2.897996645	0.003755546	0.090851897	GOLGA2P5	55592
ENSG00000101000	386.6020829	-1.346724809	0.465170247	-2.895122415	0.003790107	0.091513444	PROCR	10544
ENSG00000115461	1345.620573	2.272292546	0.784960734	2.894784983	0.003794183	0.091513444	IGFBP5	3488
ENSG00000057704	406.3870946	-1.147542173	0.396529565	-2.893963715	0.003804121	0.091513444	TMCC3	57458
ENSG00000095752	271.4755556	-2.448250446	0.846014665	-2.893862893	0.003805343	0.091513444	IL11	3589
ENSG00000140873	9.232857628	4.178591769	1.444589755	2.892580232	0.003820916	0.091752624	ADAMTS18	170692
ENSG00000159588	75.27133418	-1.636962104	0.566134057	-2.891474348	0.003834389	0.091940752	CCDC17	149483
ENSG00000012124	178.8520882	3.102918319	1.073371258	2.890815545	0.003842436	0.091998406	CD22	933
ENSG00000286156	27.51335433	-2.440987542	0.845037046	-2.888616011	0.003869413	0.092451293	NA	NA
**ENSG00000080503**	741.099464	1.048677268	0.363071442	2.888349635	0.003872691	0.092451293	SMARCA2	6595
ENSG00000149328	108.680772	2.492234372	0.863349511	2.886703866	0.003893005	0.092800349	GLB1L2	89944
ENSG00000198626	80.52320082	2.713169697	0.940113161	2.886003313	0.00390168	0.092871388	RYR2	6262

ENSG00000138722	38.3015763	3.429053064	1.1888884	2.884251426	0.003923454	0.093253516	MMRN1	22915
ENSG00000006327	2220.772865	-1.388407649	0.481684549	-2.88240022	0.003946581	0.093590876	TNFRSF12A	51330
ENSG00000185222	742.8889549	-1.665635454	0.57790479	-2.882197003	0.003949127	0.093590876	TCEAL9	51186
ENSG00000211644	844.0632889	3.335338775	1.157716239	2.880963973	0.00396461	0.09382142	IGLV1-51	28820
ENSG00000100558	1158.265705	-1.631850663	0.566730396	-2.879412636	0.003984167	0.094147591	PLEK2	26499
ENSG00000142661	366.5159865	-2.971763975	1.032390673	-2.878526561	0.003995376	0.094275848	MYOM3	127294
ENSG00000126878	337.1406417	2.738852135	0.951748121	2.8777069	0.004005771	0.094384539	AIF1L	83543
ENSG00000108852	51.94311317	2.415445083	0.83958521	2.876950492	0.004015386	0.094474555	MPP2	4355
ENSG00000134769	79.39542553	2.89745267	1.007321147	2.876394166	0.004022471	0.094504875	DTNA	1837
ENSG00000126895	6.182055465	3.34284038	1.162946423	2.874457769	0.004047219	0.09470461	AVPR2	554
ENSG00000154188	14.90345364	2.247451071	0.781909459	2.874311142	0.004049099	0.09470461	ANGPT1	284
ENSG00000162545	280.1907395	-1.183113936	0.411658933	-2.874014969	0.004052898	0.09470461	CAMK2N1	55450
ENSG00000187325	131.4838924	1.438971917	0.5007733	2.873499678	0.004059515	0.09470461	TAF9B	51616
ENSG00000258484	35.44195316	3.748324245	1.304463297	2.873460873	0.004060014	0.09470461	SPESP1	246777
ENSG00000138758	624.9180247	-0.69057725	0.240451677	-2.872000139	0.004078828	0.094997837	SEPTIN11	55752
ENSG00000151025	17.92034989	-4.457871997	1.552410159	-2.87158131	0.004084237	0.094997837	GPR158	57512
ENSG00000198858	1477.670852	-1.050141429	0.365946965	-2.8696547	0.004109203	0.095268102	R3HDM4	91300
ENSG00000162409	22.54070386	3.664698659	1.277240619	2.869231219	0.004114709	0.095268102	PRKAA2	5563
ENSG00000177106	2575.498423	-0.86283541	0.300733384	-2.869104179	0.004116362	0.095268102	EPS8L2	64787
ENSG00000077782	595.7327102	1.87202739	0.652580781	2.868652347	0.004122246	0.095268102	FGFR1	2260
ENSG00000268606	12.05208059	-6.348899971	2.213366697	-2.868435664	0.004125071	0.095268102	MAGEA2	4101
ENSG00000067082	4341.636408	-1.008681315	0.351897077	-2.86641004	0.004151561	0.095744282	KLF6	1316
ENSG00000221829	587.76616	1.269266613	0.443014677	2.86506673	0.004169214	0.096015579	FANCG	2189
ENSG00000177963	2101.555406	-0.893111059	0.311881979	-2.863618674	0.004188319	0.096319519	RIC8A	60626
ENSG00000105136	145.1899247	-1.165744576	0.407611932	-2.859937319	0.004237247	0.09718575	ZNF419	79744
ENSG00000125170	431.2045874	-0.962832835	0.336668023	-2.859887988	0.004237907	0.09718575	DOK4	55715
ENSG00000168040	673.6346132	-1.429282257	0.499947443	-2.858865022	0.004251596	0.097362754	FADD	8772
ENSG00000143578	120.7695336	1.212769535	0.424475747	2.857099715	0.004275315	0.0977686	CREB3L4	148327
ENSG00000100154	123.0194278	1.577235925	0.552485976	2.85479812	0.004306419	0.0982286	TTC28	23331
ENSG00000244731	19.36981135	3.12458233	1.094531996	2.854719956	0.004307479	0.0982286	C4A	720

ENSG00000147509	155.4868126	-2.067659233	0.724878561	-2.852421557	0.004338752	0.098538758	RGS20	8601
ENSG00000148834	1493.938949	-1.139794264	0.399708422	-2.851564295	0.004350469	0.098538758	GSTO1	9446
ENSG00000150394	45.65008047	-4.242403065	1.487749399	-2.851557573	0.004350561	0.098538758	CDH8	1006
ENSG00000115107	746.217428	-1.139505684	0.399611021	-2.851537177	0.00435084	0.098538758	STEAP3	55240
ENSG00000157168	990.5759614	-2.29897613	0.806352256	-2.851081662	0.004357078	0.098538758	NRG1	3084
ENSG00000149050	18.0878113	2.80400983	0.983550356	2.850906224	0.004359482	0.098538758	ZNF214	7761
ENSG00000105974	3874.55319	-1.130734465	0.396662403	-2.850621731	0.004363384	0.098538758	CAV1	857
ENSG00000167770	2170.896659	-0.957169861	0.335996177	-2.848752242	0.004389105	0.098934007	OTUB1	55611
ENSG00000133101	319.8501263	-3.016120867	1.05895743	-2.848198409	0.00439675	0.098934007	CCNA1	8900
ENSG00000128595	2718.540584	-0.900237234	0.316104155	-2.847913319	0.004400691	0.098934007	CALU	813
ENSG00000084453	19.20166836	-7.28839721	2.559697102	-2.84736706	0.00440825	0.098934007	SLCO1A2	6579
ENSG00000277883	56.74785381	-2.717304481	0.954393784	-2.847152324	0.004411225	0.098934007	NA	NA
**ENSG00000162892**	854.7161674	-3.533522671	1.241610125	-2.845919664	0.004428337	0.099181356	IL24	11009
ENSG00000198467	4334.599584	1.954336041	0.687252202	2.843695568	0.004459363	0.099739258	TPM2	7169

Supplementary Table 3: HPV DNA Integration Site Genome Coordinates with HC+NGS Sequence Junction Read Counts.

			Left Junction		Left Junction			Right Junction		Right Junction		
Tumor	HPV	Chr.	Human Genome	Strand	HPV Genome	Strand	Counts	Human Genome	Strand	HPV Genome	Strand	Counts
TUM2	HPV16	Chr2	183,028,932	(-)	5588	(+)	471	183,028,924	(-)	3582	(+)	395
TUM4	HPV16	Chr5	56,541,344	(-)	4178	(+)	86	56,541,431	(-)	4162	(+)	1169
TUM5	HPV16	Chr2	141,034,300	(-)	1133	(+)	2901	141,072,813	(-)	2115	(+)	71
